# Impairment of Adolescent Hippocampal Plasticity in a Mouse Model for Alzheimer's Disease Precedes Disease Phenotype

**DOI:** 10.1371/journal.pone.0002759

**Published:** 2008-07-23

**Authors:** Daniela Hartl, Michael Rohe, Lei Mao, Matthias Staufenbiel, Claus Zabel, Joachim Klose

**Affiliations:** 1 Institute for Human Genetics, Charité -University Medicine, Berlin, Germany; 2 Max-Delbrueck-Center for Molecular Medicine, Berlin, Germany; 3 Novartis Institutes for Biomedical Research, Basel, Switzerland; Mental Health Research Institute of Victoria, Australia

## Abstract

The amyloid precursor protein (APP) was assumed to be an important neuron-morphoregulatory protein and plays a central role in Alzheimer's disease (AD) pathology. In the study presented here, we analyzed the APP-transgenic mouse model APP23 using 2-dimensional gel electrophoresis technology in combination with DIGE and mass spectrometry. We investigated cortex and hippocampus of transgenic and *wildtype* mice at 1, 2, 7 and 15 months of age. Furthermore, cortices of 16 days old embryos were analyzed. When comparing the protein patterns of APP23 with *wildtype* mice, we detected a relatively large number of altered protein spots at all age stages and brain regions examined which largely preceded the occurrence of amyloid plaques. Interestingly, in hippocampus of adolescent, two-month old mice, a considerable peak in the number of protein changes was observed. Moreover, when protein patterns were compared longitudinally between age stages, we found that a large number of proteins were altered in *wildtype* mice. Those alterations were largely absent in hippocampus of APP23 mice at two months of age although not in other stages compared. Apparently, the large difference in the hippocampal protein patterns between two-month old APP23 and *wildtype* mice was caused by the absence of distinct developmental changes in the hippocampal proteome of APP23 mice. In summary, the absence of developmental proteome alterations as well as a down-regulation of proteins related to plasticity suggest the disturption of a normally occurring peak of hippocampal plasticity during adolescence in APP23 mice. Our findings are in line with the observation that AD is preceded by a clinically silent period of several years to decades. We also demonstrate that it is of utmost importance to analyze different brain regions and different age stages to obtain information about disease-causing mechanisms.

## Introduction

The *Amyloid precursor protein* (APP) plays a central role in Alzheimer's disease (AD) pathology. It was implicated in a variety of cellular processes such as axonal transport, cell adhesion, cholesterol metabolism or gene transcription and assumed to be an important neuro-morphoregulatory protein [1]. Furthermore, APP is already expressed at high levels in the developing nervous system where it is localized at regions of neuronal motility and synapse formation [2]–[4]. In addition, APP is also considered to act as a “molecular hub” protein in the cellular protein network [5]. According to scale-free interaction network theory, the disruption of a hub which possesses many connections will have a more drastic impact on the entire network than disruptions at sites with few connections. In line with this, mutations in APP or the APP-cleaving enzymes presenilin 1 and 2 are implicated in early-onset familial AD cases, whereas the numerous risk factors identified for non-familial AD cases characterize late onset disease.

In order to study AD, numerous mouse models are available. In these mice, a gene of particular interest such as APP is knocked out, mutated and/or overexpressed. When the effect of genome modifications is subsequently screened at the molecular level, usually a large number of mRNA and protein changes are observed [6]–[8]. The cellular proteome is a highly interconnected protein network that is among other restrictions dependent on resources such as space, metabolites and unbound water to allow protein diffusion. If the concentration of one protein or a larger number of proteins is altered, this affects functionally linked proteins by altering relative concentrations of those proteins to avoid macromolecular crowding [9], [10].

When analyzing the proteome of human patients or animal models for neurodegenerative diseases, the specificity of a disease (i.e. Alzheimer's, Huntington's or Parkinson's disease) is more likely determined by the affected brain region and not by the identity of altered proteins in the brains of patients or genetically modified mice [11]. Moreover, massive proteome alterations occur during normal development and aging in the animal model studied. Therefore, the impact of mutations on the proteome might be strongly age-dependent. Disease models are conventionally investigated at age stages where the disease phenotype is prominent. However, these analyses may be already biased by secondary effects of pathogenesis and may therefore obscure the causative process for disease occurring earlier in development.

In the study presented here we investigated APP-transgenic mice (APP23) expressing human APP^751^ which contains the Swedish double mutation [12]. In APP23 mice, transgene expression is sevenfold higher than endogenous APP. APP23 mice develop an AD-like pathology (ß-amyloid depositions) at 6 months of age. Plaques increase dramatically in size and number at older stages, occupying a substantial area of the cerebral cortex and hippocampus at 24 months of age [13], [14]. Furthermore, region-specific neuronal loss [7] and progressive age-related impairment of cognition [15]–[18] were observed with increasing age.

To analyze the age-specific impact of transgenic APP on the brain proteome, we designed a time course starting at very early stages where no phenotypes were reported so far. We investigated age-stages spanning adolescence (1 and 2 months of age) and adulthood (7 and 15 months of age) of mice [19]. Furthermore, the embryonic stage at day 16 *post coitum* (ED16) which represents late neurogenic phase of mouse brain development [20], was investigated. To analyze the tissue-specificity of transgenic APP in different brain regions, hippocampal (H) as well as cortical (C) proteomes of APP23 mice were investigated.

Our results show a large number of protein changes in the proteomes of APP23 mice at prenatal stages. However, the largest number of alterations was observed during adolescence in the hippocampal region, where brain plasticity is predominant. Together, our results indicate a perturbance of hippocampal plasticity in adolescent APP23 mice which may result in the development of memory deficits later during disease progression.

## Results

In the study presented here, we analyzed the cortical and hippocampal proteomes of the AD mouse model APP23 using a 2-dimensional gel-electrophoresis (2-DE) based proteomics approach. Protein spot patterns of cortex and hippocampus obtained from transgenic and *wildtype* mice at 1, 2, 7 and 15 months of age as well as cortices of 16 days old embryos were investigated (n = 6).

**Figure 1:**
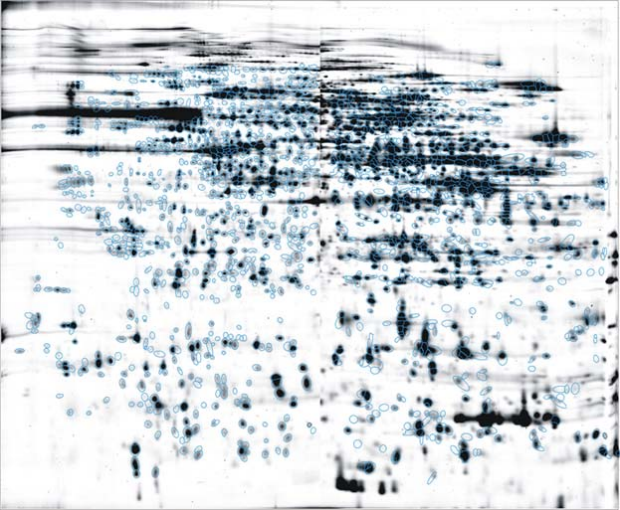
Standard pattern of protein spots analyzed. A protein spot pattern comprising 1769 spots (indicated with blue circles on a hippocampus spot pattern of a 7 months old *wildtype* mouse) was analyzed on all gels within this study.

We used our highly reproducible and extensively validated large-gel 2-DE technology [21] in combination with 2-D fluorescence difference gel electrophoresis (DIGE). *Wildtype* and transgenic samples were always labeled with the same dye (Cy3) to avoid dye-specific spot abundance variations (false positives). To allow data comparison among groups, we predefined a fixed group of 1769 spots (figure 1) that was analyzed at all age stages and brain regions. Thus, every protein spot had the same spot identification number (ID) on spot patterns of all stages and brain regions. Only 2-D patterns of embryonic day 16 brains were analyzed separately due to major differences of the embryonic compared to adult spot patterns.

### Alterations in protein abundance

**Figure 2:**
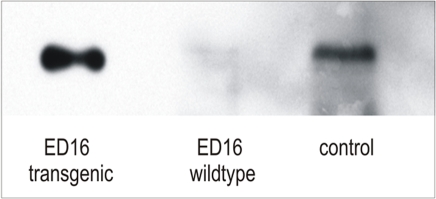
Trangenic APP expression at ED16. Immunoblot of human APP (antibody clone 6E10) with APP23 (Trans) and *wildtype* (WT) cortex tissue of 16 days old mouse embryos. A strong signal of human APP is seen on the left lane. This signal is absent in *wildtype* tissue (middle lane). The right lane shows the human APP-signal of a positive control sample (cortex tissue, 7 months, APP23-mouse).

**Figure 3:**
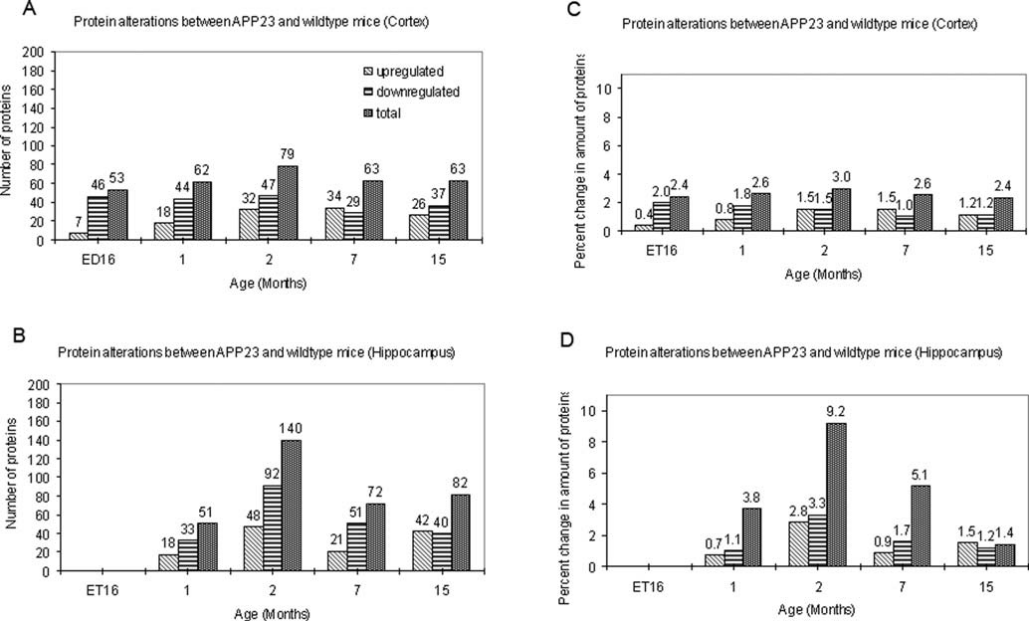
Alteration in protein number and concentration during disease progression in APP23 mice. Numbers (A and B) or volumes (corresponding to relative protein amounts; C and D) of protein spots significantly altered in APP23 mice are shown for different ages (x-axis) and brain regions (cortex: A and C; hippocampus: B and D) investigated. The values supplied represent the numbers of significantly changed spots (A and B) or a percentage of the spotvolume for 1769 spots (C and D). Upregulated spots are shown in cross striated bars, downregulated spots are shown in horizontally striated bars and the sum of both is shown by dotted bars. Many spots were altered at all stages but 2 months of age, where a peak in alteration was observed in hippocampus tissue.

When comparing 2-DE spot patterns of APP23 versus *wildtype* brain tissues, we found that many protein spots were significantly (p≤0.05) altered in abundance in APP23 brain tissue even as early as in ED16. At this stage, expression of transgenic APP was already present (figure 2, figure 3A and B).

When comparing the total number of variant proteins at each stage, we made an interesting observation. A considerable peak in protein alterations was detected in hippocampus of 2 months old APP23 mice. At this stage, 140 protein spots (7.9%) were altered in contrast to 51 spots (2.9%) at 1 month, 72 spots (4.1%) at 7 months and 82 spots (4.6%) at 15 months (figure 3B). In cortex, a smaller peak of alterations was detected at the same age. 79 spots (4.5%) were altered at 2 months in contrast to 62 spots (3.5%) at 1 month and 63 spots (3.6%) at 7 and 15 months (figure 3A). Similar results were obtained when the relative protein concentration instead of the number of variant proteins was analyzed (figure 3C, 3D). Total protein amounts changed at each stage were calculated as sums of the spot volumes of all significantly altered spots. This sum corresponds to the total change in protein concentration.

When comparing the numbers of up- versus down-regulated protein spots (figure 3A and B) as well as their protein concentrations (figure 3C and D), we observed that down-regulation predominated up-regulation at early stages. In hippocampus, this was observed in 1, 2 and 7 months old APP23 mice. In cortex, predominant down-regulation was observed in ED16 and in 1 month old mice. In older stages, up- and downregulation was more balanced.

In order to monitor proteome alterations related to development, age stages 1 versus 2, 2 versus 7 and 7 versus 15 months were compared. Comparisons were made within *wildtype*, transgenic, cortex and hippocampus groups.

**Figure 4:**
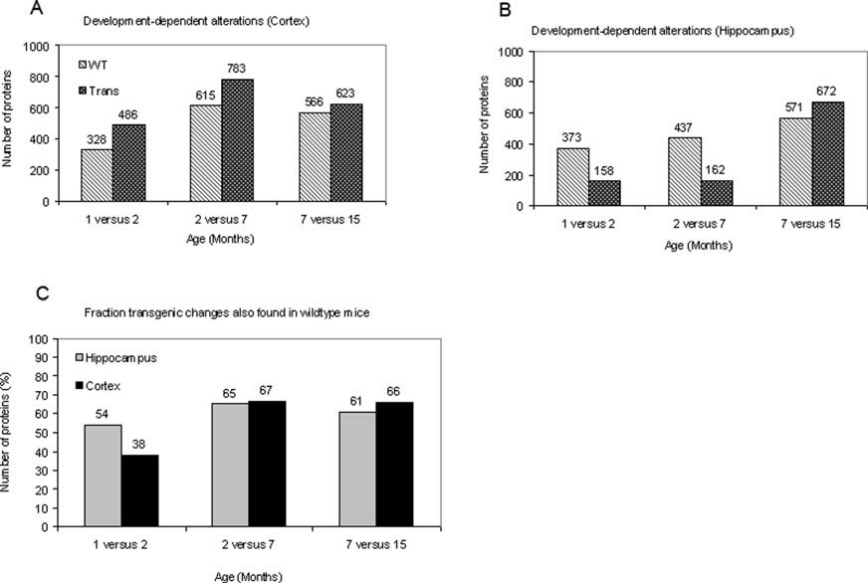
Developmental changes in APP23 and *wildtype* mice. Numbers of (y-axis) protein spot alterations associated to development are shown for *wildtype* (light grey bars) and transgenic (dark grey bars) cortex (A) and hippocampus (B) 2-D spot patterns. In (C), the fraction of proteins altered in transgenic mice that were also found in *wildtype* mice is shown. The x-axis indicated the age stages which were compared.

As shown in figure 4A, a higher number of proteins was generally altered within transgenic mice when compared to *wildtype* mice. However, in hippocampus, both stage comparisons including the 2 months-stage showed a remarkable exception to this rule. There the number of altered protein spots was considerably lower within the transgenic group (figure 4B). Only about 9% of investigated proteins were altered in APP23 mice whereas 23% of proteins were altered in *wildtypes* during the same period. In detail, 328 (*wildtype* mice) and 486 (APP23 mice) protein spots were altered in cortex between 1 and 2 months of age, respectively. During the same time period, 373 spots were altered in hippocampus of *wildtype* mice, but only 158 spots were altered in APP23 mice. Between 2 and 7 months of age, 615 (*wildtype* mice, cortex), 783 (APP23 mice, cortex), 437 (*wildtype* mice, hippocampus) and only 162 (APP23 mice, hippocampus) spots were altered. In the later time points studied (7 to 15 months), fewer proteins were altered in comparison to the younger stages. In cortex, 566 (*wildtype* mice) and 623 (APP23 mice) spots were altered and in hippocampus, 571 (*wildtype* mice) and 672 spots (APP23 mice) were altered, respectively.

Of the proteome alterations during development, 38% to 67% of proteins that were altered in APP23 mice during aging were also altered in *wildtype* mice (figure 4 C).

To exclude the possibility of systematic bias introduced by differences in spot pattern quality, mean standard deviations of spot volumes were compared between all groups. However, no significant differences were detected (data not shown).

In summary, when comparing cortex spot patterns of APP23 versus *wildtype* mice, we found that a fraction of 3.2% to 4.5% of investigated protein spots was significantly altered at all stages. In hippocampus patterns, 2.9% to 7.9% of investigated protein spots were altered. Interestingly, in hippocampus of 2 months old mice, a significant peak in variant proteins was observed. When investigating proteome alterations related to development, we found that the observed peak in hippocampus of 2 months old mice was mainly caused by alterations in the proteome of *wildtype* mice during adolescence. These alterations were largely absent in APP23 mice.

### Functional analysis of altered proteins

65% of the protein spots altered significantly between transgenic and *wildtype* mice were identified by mass spectrometry which amounts to 293 non-redundant proteins as determined by their different gene names (details in supplementary table S1).

About 90% of the identified proteins were subsequently grouped into seven functional categories. The categories were then hierarchically listed according to the percentage of altered proteins they include. This was possible since the distribution of proteins over categories was relatively similar among adult age stages. With the exception of ED 16, the most abundant category was “metabolism”, followed by “cytoskeleton”, “signal transduction”, “transcription, translation and nucleotide metabolism”, “degradation” and “folding, sorting”. The last category was “cell growth and death” (table 1). Within proteins altered in ED16, the category “transcription, translation and nucleotide metabolism” accounted for the most pronounced protein group and no protein was included in the categories “cytoskeleton” and “cell growth and death”.

**Table 1:** Percentages of proteins altered in hippocampus (H) and cortex (C) of ED16 or 1, 2, 7 and 15 months old transgenic mice, grouped into functional categories.

Functional category	1H	2H	7H	15H	ED16	1C	2C	7C	15C
**1. Metabolism**	**34**	**25**	**33**	**35**	**16**	**34**	**23**	**30**	**40**
**1.1 CH Metabolism**	13	9	15	19	10	16	7	10	13
**1.2 Energy Metabolism**	10	4	5	2	3	9	3	7	6
**1.3 AA Metabolism**	5	2	7	6	3	5	5	9	4
**1.4 Lipid Metabolism**	7	9	5	8	0	5	8	5	17
**2. Cytoskeleton**	**10**	**10**	**14**	**19**	**0**	**8**	**13**	**10**	**13**
**3. Signal Transduction**	**7**	**9**	**13**	**15**	**13**	**6**	**8**	**13**	**2**
**4. Folding, Sorting**	**7**	**11**	**7**	**8**	**3**	**9**	**12**	**9**	**8**
**5. Transcription, Translation, Nucleotide Metabolism**	**3**	**8**	**10**	**2**	**39**	**14**	**13**	**9**	**4**
**6. Degradation**	**5**	**8**	**8**	**6**	**19**	**8**	**0**	**10**	**10**
**7. Cell Growth and Death**	**2**	**2**	**3**	**4**	**0**	**6**	**2**	**2**	**6**

Although the functional distribution was similar in different age stages and brain regions, most proteins (126 proteins) were altered only at a single stage and brain region, that is, they were stage specific. Only 80 proteins were altered in two conditions (stages and/or brain regions), 46 proteins were altered in three and 28 proteins were altered in four conditions. Only thirteen proteins were found to be altered in five or more conditions (table 2).

**Table 2:** Proteins altered in five or more conditions (time points and tissues): cortex (C) or hippocampus (H) of ED16, 1, 2, 7 and 15 months old APP23 mice.

Protein name	Gene name	1H	2H	7H	15H	ED16	1C	2C	7C	15C
Apolipoprotein E precursor (Apo-E)	Apoe	▴		▴	▴			▴	▴	▴
ATP synthase subunit beta, mitochondrial [Precursor]	Atp5b	▾		▪	▾				▪	▾
ATP synthase D chain, mitochondrial	Atp5h			▴		▴	▴	▴	▴	
diazepam binding inhibitor isoform 2	Dbi	▴	▾		▾				▾	▴
Dihydropyrimidinase-related protein 2	Dpysl2	▴	▴	▴				▴		▾
enolase 2, gamma neuronal	Eno2	▾	▴	▪	▴		▴		▴	▴
Guanine nucleotide-binding protein G(I)/G(S)/G(T) subunit beta 1	Gnb1	▾	▾	▾	▾			▾	▾	
L-lactate dehydrogenase B chain	Ldhb			▾	▾		▾		▾	▾
Phosphoglycerate kinase 1	Pgk1		▾	▾			▾	▾	▾	
protein (peptidyl-prolyl cis/trans isomerase) NIMA-interacting 1	Pin1		▴	▴	▾		▾			▴
Transcriptional activator protein Pur-alpha	Pura	▴		▴	▴		▴		▴	▴
Septin-7		▾	▾	▾	▴		▾		▴	
triosephosphate isomerase	Tpi1	▴	▪		▴		▴		▾	▾

▴ upregulated.

▾ downregulated.

▪ up- and downregulated.

To test the impact of our results on human AD, we compared our data to three 2-DE-based proteomic studies of human AD. All three studies yielded a total of 30 disease-related proteins in human brain tissue of AD patients. We found 22 of the 30 proteins in our study (table 3).

**Table 3:** Proteins altered in APP23 mice (cortex (C) or hippocampus (H) of 1, 2, 7 or 15 months old mice) and human *post mortem* AD brains.

Reference	Protein name	Gene name	1H	2H	7H	15H	1C	2C	7C	15C
[35]	Gamma-actin	Actg1		▾	▪	▾			▴	
[34]	Adenylate kinase 1	Ak1			▾					
[34]	Aldolase 1, A isoform	Aldoa	▴	▴	▴	▴				
[34]	Aldolase 3, C isoform	Aldoc			▾					
[33]	ATP synthase, H+ transporting, mitochondrial F1 complex, alpha subunit, isoform 1	Atp5a1					▪			
[33]	ATP synthase subunit beta, mitochondrial	Atp5b	▾		▪	▾			▪	▾
[35]	NG,NG-dimethylarginine dimethylaminohydrolase 1	Ddah1		▾	▾	▾				
[34]	Dihydropyrimidinase-related protein 2	Dpysl2	▴	▴	▴			▴		▾
[34]	Enolase 1, alpha non-neuron	Eno1	▴	▴	▴	▴	▴			
[35]	Enolase 2, gamma neuronal	Eno2	▾	▴	▪	▴	▴		▴	▴
[33]	Fatty acid-binding protein, heart	Fabp3		▾		▾				▴
[34]	Glyceraldehyde-3-phosphate dehydrogenase	Gapdh	▴		▾		▾		▾	
[33]	Glial fibrillary acidic protein	Gfap	▾			▴	▾			▴
[33]	Guanine nucleotide-binding protein G(I)/G(S)/G(T) subunit beta 1	Gnb1	▾	▾	▾	▾		▾	▾	
[34]	Heat shock protein 8	Hspa8		▴			▴			▴
[33]	Heat shock protein 65	Hspd1		▴	▴			▴	▴	
[33]	Alpha-Internexin	Ina							▾	
[34]	Pgam1 protein	Pgam1		▾						
[34]	Protein (peptidyl-prolyl cis/trans isomerase) NIMA-interacting 1	Pin1		▴	▴	▴	▾			▴
[34]	Peroxiredoxin-2	Prdx2							▾	▴
[34]	Triosephosphate isomerase	Tpi1	▴	▪		▾	▴		▾	▾
[34], [35]	Ubiquitin carboxy-terminal hydrolase L1	Uchl1		▪	▾				▾	

▴ up-regulated.

▾ down-regulated.

▪ up- and down-regulated.

Furthermore, since APP is thought to be involved in neuronal plasticity, we determined all proteins which were altered in APP23 mice and might indicate changes in neuronal plasticity. Proteins were selected if they are structural components of synapses or if they are implicated in the dynamics of neurogenesis and synaptogenesis (table 4). Those proteins were termed neuron-specific because they have neuron-specific functions in the brain and may thus help to identify the role of mutated APP towards neuronal plasticity.

**Table 4:** Neuron-specific proteins altered in cortex (C) or hippocampus (H) of 1, 2, 7 or 15 months old APP23 mice.

Protein name	Gene name	Protein function in neurons	1H	2H	7H	15H	1C	2C	7C	15C
Brain abundant, membrane attached signal protein 1	Basp1	Regulation of the synaptic cytosceleton [42]						▾		▾
Complexin-1	Cplx1	Modulation of neurotransmitter release, more abundant in inhibitory synapses [43], [44], [45,46,47]		▾				▾		
Complexin-2	Cplx2	Modulation of neurotransmitter release, more abundant in excitatory synapses [43], [44], [45,46,47]						▴	▴	
Diazepam binding inhibitor isoform 2	Dbi	Modulation of the GABA(A) receptor, overexpression in mice is associated to deficits in hippocampal learning [48,49]	▴	▾		▴			▾	▴
Postsynaptic density protein 95	Dlg4	Structural component of the postsynaptic compartment [50]		▾			▾	▾		
Dihydropyrimidinase-related protein 2	Dpysl2	Regulation of microtubule assembly in neurons [51]	▴	▴	▴			▴		▾
Neuromodulin	Gap43	Regulation of the cytosceleton, marker for neurogenesis and synaptic plasticity [27]	▾	▾			▾	▾		
Beta-soluble NSF attachment protein	Napb	Component of the SNARE complex[52]			▾					
Gamma-soluble NSF attachment protein	Napg	Component of the SNARE complex[53]		▾		▾			▾	
Neuron derived neurotrophic factor	Nenf	Role in cell proliferation and differentiation during neurogenesis [54]		▾						
Protein kinase C and casein kinase substrate in neurons 1	Pacsin1	Role in endocytosis of synaptic vesicles [55]		▾	▾		▾			
Septin-7	Sept7	Structural component of dendritic spines [56,57]	▾	▾	▾	▾	▾		▴	
Synaptosomal-associated protein 25	Snap25	Component of the SNARE complex [58]			▾					
Syntaxin-binding protein 1	Stxbp1	Regulation of the SNARE complex [59]		▴		▾				▾
Synapsin I	Syn1	Synaptic protein, involved in synaptogenesis and neurotransmitter release [60]						▴		
Synapsin-2	Syn2		▴	▾			▴			
Synapsin Ib	SynI			▪				▪		▾

▴ up-regulated.

▾ down-regulated.

▪ up- and down-regulated.

Within neuron-specific proteins, twelve proteins were altered in hippocampus of two-month old APP23 mice. In cortex of two-month old APP23 mice, eight neuron-specific proteins were altered. In the cortex and hippocampus of the other age-stages, always four or five neuron-specific proteins were altered, respectively. In brain regions of all age-stages, down-regulation of neuron-specific proteins was predominant. In hippocampus of two-month old APP23 mice for example, nine proteins were down-regulated and only two proteins were up-regulated. One protein, *Synapsin Ib*, which occurred as more than one spot on the 2-DE pattern was up- and down- regulated depending on the protein isoform.

**Figure 5:**
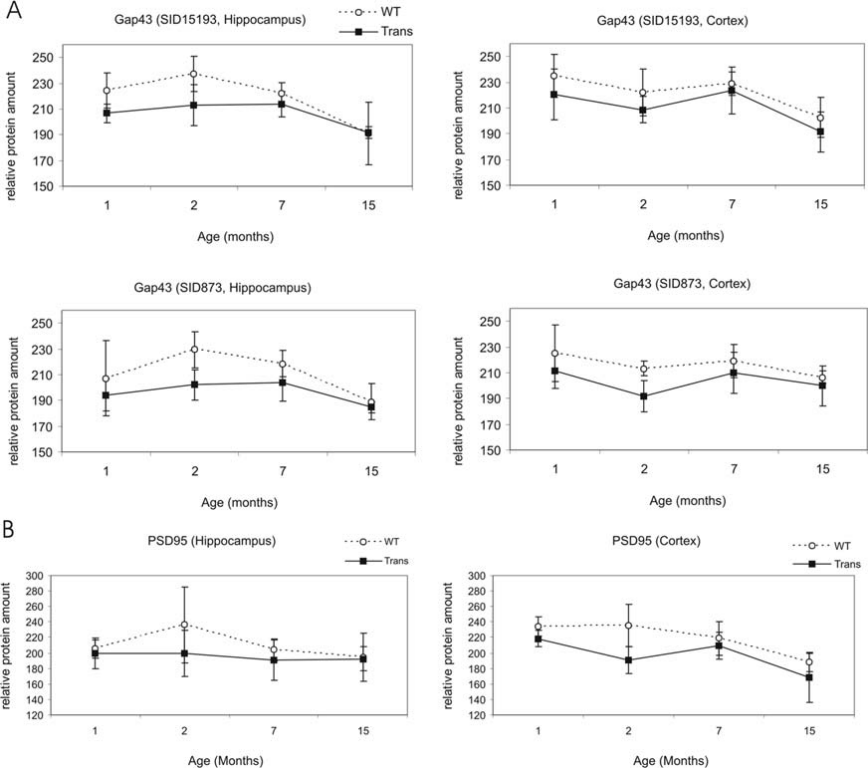
Expression levels of Neuromodulin (Gap43) and Post-synaptic density protein 95 (PSD95) during disease progression. Expression for APP23 (solid squares) and *wildtype* mice (open circles) is shown. A: Two Gap43 spots (Spots SID15193 and SID873) in hippocampus (left) and cortex (right) are shown. Significant differences (p≤0.05; Student's t-test) in spot abundance were observed in the hippocampus and cortex between 2 and 7 months of age (only *wildtype* mice) and between 7 and 15 months of age (*wildtype* and APP23 mice) as well as in the cortex between 1 and 2 months of age (only APP23 mice). Between *wildtype* and APP23 mice, significant differences in Gap43-expression were observed at 1 and 2 months of age in both brain regions. In general, expression of Gap43 was higher in younger *wildtype* as compared to APP23 mice. During aging, expression of Gap43 decreased in both, APP23 and *wildtype* mice and differences disappeared. B: Expression of PSD95 in hippocampus (left) and cortex (right). Significant differences in spot abundance were observed in the cortex between 1 and 2 (only APP23 mice) as well as between 7 and 15 months of age (*wildtype* and APP23 mice). Between *wildtype* and APP23 mice, significant differences in PSD95-expression were observed at 1 (only cortex) and 2 months (hippocampus and cortex) of age.

We analyzed the expression profiles of the two very important neuron-specific proteins *Neuromodulin* (*Gap43*) and *Post-synaptic density protein 95* (PSD95, *Dlg4*) in more detail. As shown in figure 5, the expression of Gap43 was significantly down-regulated during aging in hippocampus and cortex of *wildtype* mice from 2 to 7 (p_Hip_ = 0.04) as well as from 7 to 15 months of age (p_Hip_ = 0.002; p_Cor_ = 0.005). In APP23 mice, Gap43-expression was also down-regulated during aging (7 to 15 months) in both brain regions (p_Hip_ = 0.001; p_Cor_ = 0.008). It was also down-regulated between 1 and 2 months of age in cortex (p = 0.025) but not hippocampus. Moreover, Gap43 was down-regulated in hippocampus of one (p = 0.03) and two (p = 0.004) months old APP23 when compared to *wildtype* mice. Figure 5B shows the expression profile of PSD95 during disease progression. This protein was significantly down-regulated in the cortex of APP23 mice when comparing 1 and 2 months old mice (p = 0,007) and in the cortex of APP23 as well as *wildtype* mice when comparing 7 and 15 months old mice (p_APP23_ = 0.032; p_wt_ = 0.017). Furthermore, expression of PSD95 was significantly down-regulated in APP23 mice as compared to *wildtype* mice at 1 month in cortex (p = 0.049) and at 2 months of age in hippocampus (p = 0,019) and cortex (p = 0.043).

## Discussion

In this study, the APP23 mouse model for AD was investigated using a 2-DE proteomics approach. We analyzed the neocortex and hippocampus of 1, 2, 7 and 15 months old mice. In addition, the neocortex of 16 days old mouse embryos was investigated.

When comparing the 2-DE protein patterns of APP23 mice against those of *wildtype* mice, we detected that about 4% (70 protein spots) of all protein spots were altered in abundance. This large number of protein expression changes was observed at all age stages and brain regions except for hippocampus of two-month old mice. Here, twice as many (8%) protein spots were altered. To elucidate this unexpected observation we compared protein patterns of APP23 or *wildtype* mice longitudinally between all ages investigated. Interestingly, we found a large number of proteome alterations related to development including the two-month age stage in the *wildtype* hippocampus but not in the hippocampus of APP23 mice. This may indicate that impairment of brain maturation precedes the intrinsic disease process.

Beta-amyloid deposits first appear when APP23 mice are six months old. Deposits occupy a substantial area of the cerebral cortex and hippocampus at 21 months of age. In those old-aged animals, a very large number of protein expression differences can be observed (data not shown) but they might rather be the consequence of secondary alterations due to inflammatory reactions as well as neuritic and synaptic degeneration [22]. According to our results, brain maturation might be impaired at much younger age stages preceding beta-amyloid deposition. This is in line with findings that synaptic dysfunction, synaptic loss and learning deficits in transgenic mouse models of AD appear prior to amyloid plaque deposition [23]–[26].

The developmental period around two months of age represents adolescence in mice. During that age, mesocorticolimbic brain regions are exceedingly plastic in terms of synaptic reorganization and adult neurogenesis [27]–[29]. With the transition to adulthood and during subsequent aging, brain plasticity is gradually reduced. APP was assumed to be a neuron morphoregulatory protein and is therefore involved in plasticity associated dynamics [5], [30]. This would imply that when APP function is disturbed, this might predominantly affect the brain during adolescence - the age phase of enhanced plasticity. Accordingly we observed a significant reduction in proteome alterations related to development which resulted in a large difference between the proteomes of APP23 and *wildtype* mice. Importantly, this was observed in adolescent but not in adult mice.

Processes that contribute to brain plasticity are the formation and degradation of synapses, modulation of synaptic strength as well as neurogenesis. Of all proteins changed in APP23 mice, those which are most likely involved in neuronal plasticity due to their selective expression at synapses or their up-regulation during neurogenesis were analyzed in more detail. Interestingly, the majority of these proteins were altered in hippocampus of two-month old APP23 mice. Furthermore, those proteins were predominantly down-regulated in APP23 mice. For example, *neuromodulin* (gene name *Gap43*), which is widely used as marker protein for neurogenesis and synaptic plasticity [31], was down-regulated in hippocampus of one and two-month old APP23 versus *wildtype* mice. During aging (7 to 15 months of age), neuromodulin expression was down-regulated to the same expression level in both, APP23 and *wildtype* mice. Another synaptic protein, PSD95 was down-regulated in hippocampus and cortex of APP23 mice during adolescence. In addition, this protein was later down-regulated after 7 months in cortex of both, APP23 and *wildtype* mice and was expressed on the same level in both mice. Therefore differential expression of PSD95 and Gap43 between APP23 and *wildtype* mice was specific to adolescence.

Taken together, the absence of developmental proteome alterations as well as the predominant down-regulation of neuron-specific proteins in APP23 mice indicate an interference of transgenic APP with mechanisms that generate the naturally occurring peak in hippocampal plasticity during adolescence in *wildtype* mice.

Recently, it was reported that low concentrations of natural, soluble Aß (which is enhanced in many mouse models for AD, such as APP23) can alter dendritic spine number, morphology and dynamics in hippocampal neurons [32], [33]. Accordingly *Lanz et al.* made an interesting observation when counting dendritic spines of hippocampal CA1-neurons in two different mouse models for AD. They observed the greatest loss of dendritic spines in adolescent transgenic mice. Differences in the number of dendritic spines then disappeared in older, plaque-bearing transgenic mice [25]. When the behavior of APP23 mice was investigated, major learning and memory deficits were found as early as 3 months [17]. Those results are quite compatible with our observations on the proteome level.

In the neocortex, we did not detect a general decrease in proteome alterations related to development in APP23 mice. In this brain region, progression of brain maturation during adolescence appears to be very region-specific. Moreover, differing types of neurons and synapses show differences in vulnerability to Aß-induced degeneration [34]. In line with this, a decrease in the total neocortical synapse number has not been detected in APP23 mice [35]. In contrast, only in neocortical pyramidal neurons has a decrease in spine density been demonstrated in mice carrying human APP bearing the Swedish mutation [36]. We therefore speculate, that since the neocortex is a very heterogenous brain region, observations concerning a disturbed plasticity might be hard to detect when the entire cortex is analyzed.

We identified 293 proteins altered in the APP23 mouse model for AD. Comparing our data to proteomic studies performed with *post mortem* human brain tissue of AD patients [37]–[39] we found that 22 out of 30 published proteins were altered in both, human AD patients and in our study of APP23 mice. In addition, we identified many proteins such as *Apolipoprotein E* (gene name *Apoe*) [40], *Peptidyl-prolyl cis/trans isomerase NIMA-interacting 1* (gene name *Pin1*) [41], and numerous other proteins that have already been implicated in AD. Although the distribution of altered proteins over functional categories was similar among all postnatal stages, most protein alterations were stage and/or brain region specific. The latter fact also demonstrates that the effect of a mutation on the proteome is highly age- and tissue- dependent.

In conclusion, we found a large number of protein expression differences throughout the entire lifespan of APP23 mice, beginning at ED16, a phase where neurogenesis is predominant in the developing mouse brain suggesting an early impact of transgenic APP. This finding correlates with the observation that APP has an important role during embryonic neurogenesis [30]. Interestingly, during adolescence, rather specific proteome alterations were observed in the hippocampus. Based on the cellular localization of the proteins altered we conclude that a naturally occurring peak in hippocampal plasticity was absent in APP23 mice. This might be a transient effect of mutated APP on adolescent plasticity. Still, the deficiency may cause a longterm perturbance of the neuronal network finally resulting in memory impairment in aging APP23 mice.

Our findings illuminate the process during the clinically silent period of several years or decades in AD. Synaptic degeneration, which is the major structural correlate to cognitive dysfunction is a slow process initiated by a failure of local regulatory mechanisms of synaptic plasticity [42] which we demonstrated in adolescent APP23 mice. Nevertheless, we need more information to elucidate how exactly the changes found on the proteome level translate to alterations in cellular morphology and phenotype which finally lead to AD.

## Materials and Methods

### Mouse models and tissues

We investigated the APP23 mouse model for AD with a 7 fold over-expression of hAPP751 carrying the Swedish double-mutation [12]. These mice have been backcrossed to the C57Bl/6 strain for over 20 generations. We investigated cortices of mouse embryos (E16) as well as cortices and hippocampi of 1, 2, 7 and 15 months old male APP23 mice as well as *wildtype* littermates. Sample size was n = 6 (biological replicates) for all groups within the study.

### Protein Extraction and Separation Procedure

Each protein extract was prepared from individual brain regions of single mice according to our updated protein extraction protocol [10]. Briefly, frozen tissue samples together with sample buffer (50 mM TRIZMA Base (Sigma-Aldrich, Steinheim, Germany), 50 mM KCl and 20% w/v glycerol at pH 7.5) as well as a proteinase inhibitor cocktail (Complete, Roche Diagnostics) were ground to fine powder in liquid nitrogen and subsequently sonicated on ice (0°C). Afterwards, DNAse and urea were added to the samples. Individual transgenic and *wildtype* tissue samples were then labeled by Cy3 minimal dye (GE Healthcare, Munich, Germany). A pooled *wildtype* tissue sample of the relevant age and tissue was used as internal standard and labeled by Cy5 minimal dye (GE Healthcare). Labeling was carried out according to manufacturer's instructions (400 pmol fluorescent dye per 50 µg of protein). Each Cy3-labeled sample was mixed with the same amount of internal standard. The protein extracts were then supplied with 70 mM dithiothreitol (Biorad, Munich, Germany), 2% v/w of ampholyte mixture Servalyte pH 2–4 (Serva, Heidelberg, Germany) and stored at −80°C.

### Two-Dimensional Gel Electrophoresis (2-DE)

Protein samples were separated by the large-gel 2-DE technique developed in our laboratory as described previously [43]. The gel format was 40 cm (isoelectric focusing)×30 cm (SDS-PAGE)×1.0 mm (gel width). Two dimensional fluorescent protein patterns were obtained by fluorescent image acquisition at a resolution of 100 µm (laser scanner Typhoon 9400, GE Healthcare).

### Spot evaluation procedure

Protein spot patterns were evaluated by Delta2D imaging software (version 3.4 Decodon, Greifswald, Germany). Briefly, protein patterns of internal standards were matched to each other using “exact” mode of Delta2D. Subsequently, a fusion image was generated employing “union” mode, creating a protein pattern containing all spots from all 2D gels (cortex as well as hippocampus at all age stages except ED16, internal standard gels were not included) within the project. Digital spot detection was carried out on the fusion image, followed by manual spot editing. The spot pattern containing 1769 protein spots was then transferred from the fusion image to all other 2-DE images. In this way, each spot on every gel of the project had the same spot identification number.

Percent volume of spot pixel intensities was used for quantitative analysis of protein expression. Normalized values (after background extraction and normalization to internal standard) were exported from Delta2D in spreadsheet format for statistical analysis. Data sets were analyzed applying paired students t-test (*wildtype* and transgenic samples were handled in pairs from protein extraction to 2-D gel runs) when transgenic groups were compared to *wildtype* groups (n = 6). Unpaired students t-test was performed when *wildtype* or transgenic groups of different age stages were compared (n = 6). Only fold changes over 10% were considered for graphs shown but results were similar when all significantly altered spots were included. All significantly altered proteins that were identified by mass spectrometry are listed in supplementary table S1.

### Protein Identification

For protein identification by mass spectrometry, 640 µg protein extract was separated on 2-D gels and stained with a mass spectrometry-compatible silver staining protocol [44]. In order to assign corresponding protein spots between analytical fluorescent and quantitative silver stained 2-D gels reliably, spot patterns of silver stained gels were matched to CyDye stained gels using Delta2D. Protein spots of interest were excised from 2-D gels and subjected to in-gel tryptic digestion. Peptides were analyzed by a Reflex 4 MALDI-TOF mass spectrometer (Bruker Daltonics, Bremen Germany) as described previously [44]. Alternatively, ESI-tandem -MS/MS on a LCQ Deca XP ion trap instrument (Thermo Finnigan, Waltham, MA, USA) was applied. Mass spectra were analyzed using our in-house MASCOT software package (version 2.1) automatically searching NCBI databases.

MALDIM-MS ion search was performed with this set of parameters: (I) taxonomy: Mus musculus, (II) proteolytic enzyme: trypsin, (III) maximum of accepted missed cleavages: 1, (IV) mass value: monoisotopic, (V) peptide mass tolerance 0.8 Da, (VI) fragment mass tolerance: 0.8 Da, and (VII) variable modifications: oxidation of methionine and acrylamide adducts (propionamide) on cysteine. Only proteins with scores corresponding to p<0.05, with at least two peptides identified by two independent identifications each were considered. Furthermore, the molecular weight and pI of each protein identified by database search was compared to values obtained from our 2-D patterns.

### Analysis of biological functions

Gene symbols and SwissProt accession numbers were used to investigate proteins with altered expression profile in this study. Furthermore, proteins were grouped according to functional categories using parameters like GO and KEGG terms (retrieved by WEBGESTALT [45]) and by literature search.

### Immunoblotting

Protein concentration was determined using a Roti-Nanoquant assay (Carl Roth, Karlsruhe, Germany). Brain protein extracts were separated using 12% SDS-PAGE gels, blotted to PVDF membranes and probed with human Aß-antibody (clone 6E10) (Abcam, Cambridge, UK) according to standard immunoblotting procedures.

## Supporting Information

Table S1 Proteins significantly altered in transgenic mouse brain regions hippocampus (H) and cortex (C) of different ages (1, 2, 7 and 15 months) as well as in cortex of 16 days old APP23 mouse embryos (ED16).
(2.33 MB DOC)
