# Supplementary material for: Impairment of Adolescent Hippocampal Plasticity in a Mouse Model for Alzheimer's Disease Precedes Disease Phenotype
Source: PLoS One. 2008 Jul 23;3(7):e2759. doi: 10.1371/journal.pone.0002759 (PMC2447155; doi:10.1371/journal.pone.0002759)
Supplement: Table S1 — Proteins significantly altered in transgenic mouse brain regions hippocampus (H) and cortex (C) of different ages (1, 2, 7 and 15 months) as well as in cortex of 16 days old APP23 mouse embryos (ED16). (2.33 MB DOC) [file pone.0002759.s001.doc]

| **Supplementary table S1: Proteins significantly altered in transgenic mouse brain regions hippocampus (H) and cortex (C) of different ages (1, 2, 7 and 15 months) as well as in cortex of 16 days old APP23 mouse embryos (ED16).** | | | | | | | | | | | | | | |
| --- | --- | --- | --- | --- | --- | --- | --- | --- | --- | --- | --- | --- | --- | --- |
| **Stage** | **Spot ID** | **p-value (t-Test, Trans/WT)** | **Ratio (Trans/WT)** | **Protein Name** | **gi-Number** | **SwissProt Accession** | **Gene Name** | **Functional Category** | **MS- Technique^a)^** | **Sequence coverage (%)** | **No. of matched peptides ^b)^** | **Mowse Score** | **Mass (Da)** | **pI** |
| **ED16** | ESID845 | 0.050 | 0.902 | Chain , Adenosine Deaminase (E.C.3.5.4.4) Complexed With 1-Deaza-Adenosine (Daa) | gi|493801 | P03958 | Ada | Nucleotide metabolism | M | 44 | 17 | 162 | 39636 | 5.48 |
| **ED16** | ESID1471 | 0.009 | 1.048 | ATP synthase D chain, mitochondrial | gi|51980458 | Q9DCX2 | Atp5h | Energy Metabolism | M | 68 | 12 | 129 | 19000 | 5.52 |
| **ED16** | EBID777 | 0.027 | 0.946 | Calcyclin-binding protein | gi|3142331 | Q9CXW3 | Cacybp | Signal transduction | M | 71 | 20 | 205 | 28301 | 7.78 |
| **ED16** | EBID2576 | 0.047 | 0.914 | catalase | gi|74223714 | Q3UF58 | Cat | AS Metabolism | M | 39 | 18 | 211 | 59661 | 7.72 |
| **ED16** | ESID1453 | 0.009 | 0.889 | Chromobox protein homolog 1 | gi|5803076 | P83917 | Cbx1 | Transcription | M | 48 | 14 | 137 | 21000 | 4.85 |
| **ED16** | ESID1555 | 0.028 | 0.928 | chromobox homolog 3 (Drosophila HP1 gamma), | gi|6680860 | Q9DCC5 | Cbx3 | Transcription | M | 45 | 10 | 80 | 20000 | 4.96 |
| **ED16** | ESID1307 | 0.036 | 0.930 | Chloride intracellular channel protein 4 | gi|7304963 | Q9QYB1 | Clic4 |  | M | 26 | 6 | 80 | 28711 | 5.44 |
| **ED16** | ESID942 | 0.022 | 0.875 | Proto-oncogene C-crk | gi|31559995 | Q64010 | Crk | Signal transduction | M | 50 | 14 | 179 | 33794 | 5.38 |
| **ED16** | ESID933 | 0.008 | 0.916 | Eukaryotic translation initiation factor 3 subunit 2 | gi|9055370 | Q9QZD9 | Eif3s2 | Translation | M | 33 | 10 | 122 | 36438 | 5.38 |
| **ED16** | EBID1940 | 0.005 | 0.943 | Far upstream element-binding protein 1 | gi|84662730 | Q91WJ8 | Fubp1 | Transcription | M | 44 | 28 | 238 | 67401 | 7.18 |
| **ED16** | ESID430 | 0.035 | 0.824 | GMP synthase [glutamine-hydrolyzing] | gi|85861218 | Q3THK7 | Gmps | Nucleotide metabolism | M | 40 | 27 | 234 | 76675 | 6.29 |
| **ED16** | EBID1609 | 0.019 | 0.759 | HnRNP A1 [Fragment] | gi|85060507 | P70370 | Hnrpa1 | Translation | M | 34 | 10 | 139 | 38810 | 9.17 |
| **ED16** | ESID878 | 0.039 | 0.860 | Heterogeneous nuclear ribonucleoprotein A/B | gi|6754222 | Q99020 | Hnrpab | Transcription | M | 34 | 13 | 151 | 30812 | 7.68 |
| **ED16** | ESID471 | 0.014 | 0.945 | heterogeneous nuclear ribonucleoprotein K | gi|13384620 | P61979 | Hnrpk | Transcription | M | 37 | 12 | 131 | 50944 | 5.39 |
| **ED16** | ESID1822 | 0.013 | 0.816 | Huntingtin-interacting protein HYPK | gi|27229055 | Q9CR41 | Hypk | Folding | M | 39 | 7 | 77 | 14670 | 4.91 |
| **ED16** | ESID316 | 0.045 | 0.864 | KH-type splicing regulatory protein | gi|74224349 | Q3U0V1 | Khsrp | Translation | M | 35 | 24 | 229 | 76762 | 6.9 |
| **ED16** | ESID157 | 0.041 | 0.943 | Pyruvate carboxylase, mitochondrial precursor | gi|6679237 | Q05920 | Pc | CH Metabolism | M | 36 | 34 | 285 | 129602 | 6.25 |
| **ED16** | EBID2180 | 0.044 | 1.208 | Phosphoglycerate mutase family member 5 | gi|30704499 | Q8BX10 | Pgam5 | CH Metabolism | M | 37 | 11 | 150 | 31270 | 8.98 |
| **ED16** | EBID641 | 0.029 | 0.960 | Prohibitin-2 | gi|6005854 | O35129 | Phb2 | Transcription | M | 49 | 15 | 149 | 33276 | 9.83 |
| **ED16** | ESID1521 | 0.046 | 0.835 | Peroxiredoxin-2 | gi|3603241 | Q61171 | Prdx2 | Degradation | M | 37 | 7 | 115 | 21778 | 5.2 |
| **ED16** | ESID1351 | 0.007 | 0.798 | Proteasome subunit alpha type 5 | gi|7106387 | Q9Z2U1 | Psma5 | Degradation | M | 34 | 9 | 104 | 26394 | 4.74 |
| **ED16** | EBID946 | 0.026 | 0.754 | Proteasome subunit beta type 5 [Precursor] | gi|3914434 | O55234 | Psmb5 | Degradation | M | 57 | 11 | 178 | 22952 | 8.65 |
| **ED16** | ESID733 | 0.015 | 0.772 | proteasome (prosome, macropain) 26S subunit, ATPase, 4 | gi|74195574 | Q3TJ97 | Psmc4 | Degradation | M | 29 | 13 | 161 | 47319 | 5.14 |
| **ED16** | ESID499 | 0.028 | 0.755 | UV excision repair protein RAD23 homolog B | gi|45829444 | P54728 | Rad23b | Degradation | M | 17 | 7 | 84 | 43389 | 4.77 |
| **ED16** | EBID859 | 0.006 | 0.884 | RAN, member RAS oncogene family | gi|74208259 | Q3ULW0 | Ran | Signal transduction | M | 40 | 9 | 90 | 24336 | 7.71 |
| **ED16** | ESID1535 | 0.040 | 0.842 | Retinoblastoma-binding protein 9 | gi|86439977 | O88851 | Rbbp9 |  | M | 45 | 6 | 74 | 20898 | 5.64 |
| **ED16** | EBID768 | 0.032 | 1.214 | Rpp30 protein [Fragment] | gi|21706657 | Q8K238 | Rpp30 | Translation | M | 43 | 7 | 124 | 16062 | 9.03 |
| **ED16** | ESID963 | 0.029 | 0.917 | Serine-threonine kinase receptor-associated protein | gi|6755682 | Q9Z1Z2 | Strap | Signal transduction | M | 63 | 21 | 178 | 38000 | 4.99 |
| **ED16** | ESID970 | 0.022 | 0.885 | Suppressor of G2 allele of SKP1 homolog | gi|23956176 | Q9CX34 | Sugt1 | Degradation | M | 34 | 9 | 85 | 38135 | 5.32 |
| **ED16** | ESID1031 | 0.048 | 0.871 | Transaldolase | gi|33859640 | Q93092 | Taldo1 | CH Metabolism | M | 41 | 17 | 175 | 37363 | 6.57 |
| **1C** | BID571 | 0.041 | 0.885 | 3-ketoacyl-CoA thiolase, mitochondrial | gi|29126205 | Q8BWT1 | Acaa2 | Lipid metabolism | E | 12 | 4 | 216 | 43015 | 8.3 |
| **1C** | SID15201 | 0.015 | 1.057 | Actin, alpha cardiac muscle 1 | gi|387090 | P68033 | Actc1 | Cytoskeleton | E | 5 | 2 | 113 | 41758 | 5.23 |
| **1C** | BID1686 | 0.005 | 0.915 | acylphosphatase 1, erythrocyte (common) type | gi|13384810 | P56376 | Acyp1 | CH Metabolism | E | 22 | 2 | 120 | 11234 | 9 |
| **1C** | BID1142 | 0.010 | 0.933 | adenylate kinase isozyme 3 | gi|4760600 | Q9WTP7 | Ak3 | Nucleotide metabolism | E | 45 | 9 | 493 | 24625 | 8.57 |
| **1C** | SID5676 | 0.024 | 1.197 | Serum albumin precursor | gi|74137565 | P07724 | Alb | Cell growth and death | E | 42 | 25 | 1377 | 68688 | 5.78 |
| **1C** | BID442 | 0.017 | 0.952 | ATP synthase, H+ transporting, mitochondrial F1 complex, alpha subunit, isoform 1 | gi|6680748 | Q03265 | Atp5a1 | Energy Metabolism | E | 37 | 21 | 1269 | 59716 | 9.22 |
| **1C** | SID15830 | 0.045 | 1.467 | ATP synthase, H+ transporting, mitochondrial F1 complex, alpha subunit, isoform 1 | gi|6680748 | Q03265 | Atp5a1 | Energy Metabolism | E | 10 | 4 | 264 | 59716 | 9.22 |
| **1C** | SID1468 | 0.043 | 1.168 | ATP synthase D chain, mitochondrial | gi|21313679 | Q9DCX2 | Atp5h | Energy Metabolism | E | 82 | 12 | 642 | 18738 | 5.52 |
| **1C** | SID495 | 0.010 | 1.049 | ATPase, H+ transporting, V1 subunit A, isoform 1 | gi|31560731 | P50516 | Atp6v1a | Energy Metabolism | E | 34 | 16 | 1122 | 68283 | 5.42 |
| **1C** | BID403 | 0.047 | 0.945 | chaperonin subunit 4 (delta) | gi|6753322 | P80315 | Cct4 | Folding | E | 21 | 11 | 591 | 58030 | 8.24 |
| **1C** | SID15241 | 0.018 | 0.817 | Clathrin light chain B (Lcb) | gi|30794164 | Q6IRU5 | Cltb | Sorting | E | 13 | 3 | 167 | 23160 | 4.63 |
| **1C** | SID2329 | 0.023 | 0.905 | UMP-CMP kinase | gi|12832572 | Q9DCS7 | Cmpk | Nucleotide metabolism | E | 33 | 6 | 387 | 25698 | 8.13 |
| **1C** | SID5896 | 0.045 | 0.895 | copine VI | gi|6753510 | Q80W08 | Cpne6 |  | M | 15 | 20 | 83 | 61742 | 5.37 |
| **1C** | SID1419 | 0.015 | 0.851 | Proto-oncogene C-crk | gi|423455 | Q64010 | Crk | Signal transduction | M | 50 | 14 | 179 | 33794 | 5.38 |
| **1C** | BID730 | 0.004 | 0.574 | Quinone oxidoreductase | gi|13277837 | P47199 | Cryz | Energy Metabolism | E | 17 | 5 | 286 | 35276 | 8.18 |
| **1C** | SID449 | 0.049 | 0.934 | postsynaptic density protein 95 | gi|6681195 | Q62108 | Dlg4 | Neuron | E | 2 | 1 | 52 | 26390 | 5.56 |
| **1C** | BID492 | 0.010 | 0.940 | eukaryotic translation elongation factor 1 alpha 2 | gi|6681273 | P10126 | Eef1a1 | Translation | E | 6 | 3 | 159 | 50018 | 9.16 |
| **1C** | SID1251 | 0.046 | 0.844 | elongation factor 1-beta homolog | GI:5902663 | O70251 | Eef1b | Translation | E | 13 | 2 | 273 | 24693 | 4.47 |
| **1C** | BID538 | 0.042 | 1.122 | enolase 1, alpha non-neuron | gi|53734652 | Q5FW97 | Eno1 | CH Metabolism | E | 29 | 13 | 807 | 50209 | 8.01 |
| **1C** | SID824 | 0.019 | 1.060 | enolase 2, gamma neuronal | gi|7305027 | P17183 | Eno2 | CH Metabolism | E | 49 | 14 | 995 | 47267 | 4.99 |
| **1C** | BID1543 | 0.007 | 1.161 | FK506 binding protein 2 | gi|6679805 | P45878 | Fkbp2 | Folding | E | 10 | 1 | 47 | 15334 | 9.05 |
| **1C** | SID720 | 0.007 | 0.930 | FK506-binding protein 4 | gi|6753882 | P30416 | Fkbp4 | Folding | E | 26 | 10 | 557 | 51540 | 5.54 |
| **1C** | SID15193 | 0.044 | 0.939 | Neuromodulin | gi|20271449 | P06837 | Gap43 | Neuron | E | 50 | 8 | 582 | 23618 | 4.63 |
| **1C** | BID790 | 0.025 | 0.889 | similar to glyceraldehyde-3-phosphate dehydrogenase | gi|6679937 | P16858 | Gapdh | CH Metabolism | E | 6 | 2 | 106 | 35787 | 8.44 |
| **1C** | SID5893 | 0.017 | 0.815 | glial fibrillary acidic protein | gi|51066 | P03995 | Gfap | Cytoskeleton | E | 7 | 3 | 161 | 48437 | 5.29 |
| **1C** | BID530 | 0.028 | 1.053 | glutamine synthetase | gi|31982332 | P15105 | Glul | AS Metabolism | E | 35 | 12 | 677 | 42092 | 6.64 |
| **1C** | SID5890 | 0.040 | 0.898 | glutathione synthetase | gi|6680117 | P51855 | Gss | AS Metabolism | E | 5 | 4 | 241 | 52214 | 5.56 |
| **1C** | BID900 | 0.003 | 1.211 | heterogeneous nuclear ribonucleoprotein A0 isoform 2 | gi|82950644 | Q9CX86 | Hnrpa0 | Translation | E | 31 | 5 | 373 | 30512 | 9.35 |
| **1C** | SID633 | 0.015 | 0.953 | heterogeneous nuclear ribonucleoprotein K | gi|74225344 | Q3U6X2 | Hnrpk | Transcription | M | 28 | 28 | 91 | 51031 | 5.7 |
| **1C** | SID5651 | 0.009 | 1.405 | Heat shock cognate 71 kDa protein | gi|309319 | P63017 | Hspa8 | Folding | E | 33 | 17 | 1072 | 70793 | 5.37 |
| **1C** | SID631 | 0.004 | 0.823 | Heat shock protein 9A | gi|903309 | P38647 | Hspa9a | Folding | E | 7 | 4 | 294 | 73416 | 5.81 |
| **1C** | BID1869 | 0.009 | 0.870 | inositol 1,4,5-trisphosphate 3-kinase A | gi|22122643 | Q8R071 | Itpka | Signal transduction | E | 26 | 11 | 647 | 50903 | 7.64 |
| **1C** | BID775 | 0.032 | 0.583 | Voltage-gated potassium channel subunit beta-2 | gi|975314 | P62482 | Kcnab2 |  | E | 3 | 1 | 78 | 41154 | 9.19 |
| **1C** | BID853 | 0.018 | 1.042 | L-lactate dehydrogenase | gi|74217959 | Q3TCI7 | Ldha | CH Metabolism | E | 34 | 10 | 641 | 36476 | 7.62 |
| **1C** | SID1096 | 0.014 | 0.839 | lactate dehydrogenase 2, B chain | gi|6678674 | P16125 | Ldhb | CH Metabolism | E | 23 | 7 | 446 | 36549 | 5.7 |
| **1C** | SID1107 | 0.044 | 0.875 | lactate dehydrogenase 2, B chain | gi|6678674 | P16125 | Ldhb | CH Metabolism | E | 18 | 5 | 277 | 36549 | 5.7 |
| **1C** | BID363 | 0.022 | 0.854 | Lamin-A/C | gi|52865 | P48678 | Lmna |  | E | 20 | 10 | 759 | 65367 | 6.05 |
| **1C** | SID15230 | 0.028 | 0.627 | Nascent polypeptide-associated complex subunit alpha | gi|41350312 | Q60817 | Naca | Transcription | E | 6 | 1 | 94 | 23370 | 4.52 |
| **1C** | BID1234 | 0.012 | 0.927 | NADH dehydrogenase (ubiquinone) 1 beta subcomplex, 9 | gi|29789148 | Q9CQJ8 | Ndufb9 | Energy Metabolism | E | 19 | 3 | 171 | 21970 | 7.67 |
| **1C** | SID885 | 0.049 | 0.875 | NSFL1 cofactor p47 (p97 cofactor) | gi|38198665 | Q9CZ44 | Nsfl1c | Lipid metabolism | E | 44 | 14 | 836 | 40928 | 5.09 |
| **1C** | SID712 | 0.003 | 0.842 | protein kinase C and casein kinase substrate in neurons 1 | gi|6754974 | Q61644 | Pacsin1 | Cytoskeleton | E | 34 | 11 | 675 | 50544 | 5.15 |
| **1C** | SID718 | 0.049 | 0.945 | protein kinase C and casein kinase substrate in neurons 1 | gi|6754974 | Q61644 | Pacsin1 | Cytoskeleton | E | 43 | 19 | 1136 | 50544 | 5.15 |
| **1C** | SID744 | 0.014 | 0.778 | protein kinase C and casein kinase substrate in neurons 1 | gi|6754974 | Q61644 | Pacsin1 | Cytoskeleton | M | 36 | 23 | 171 | 50544 | 5.15 |
| **1C** | SID1316 | 0.021 | 0.920 | Platelet-activating factor acetylhydrolase IB beta subunit (PAF acetylhydrolase 30 kDa subunit) (PA | gi|3024348 | Q61206 | Pafah1b2 | Lipid metabolism | E | 5 | 2 | 124 | 25476 | 5.78 |
| **1C** | SID1118 | 0.003 | 0.851 | pyruvate dehydrogenase (lipoamide) beta | gi|18152793 | Q9D051 | Pdhb | CH Metabolism | E | 6 | 2 | 124 | 38912 | 6.41 |
| **1C** | SID15722 | 0.010 | 0.726 | profilin 2 | gi|9506971 | Q9JJV2 | Pfn2 | Cytoskeleton | E | 10 | 1 | 63 | 15022 | 6.55 |
| **1C** | SID15766 | 0.010 | 0.901 | profilin 2 | gi|9506971 | Q9JJV2 | Pfn2 | Cytoskeleton | E | 20 | 2 | 137 | 15022 | 6.55 |
| **1C** | SID15780 | 0.050 | 0.940 | profilin 2 | gi|9506971 | Q9JJV2 | Pfn2 | Cytoskeleton | E | 32 | 4 | 211 | 15022 | 6,55 |
| **1C** | BID1029 | 0.005 | 1.142 | phosphoglycerate mutase family member 5 | gi|12848731 | Q8BX10 | Pgam5 | CH Metabolism | E | 13 | 3 | 187 | 28678 | 9.85 |
| **1C** | BID604 | 0.030 | 0.903 | Phosphoglycerate kinase 1 | gi|129903 | P09411 | Pgk1 | CH Metabolism | E | 37 | 14 | 888 | 44508 | 7.53 |
| **1C** | BID1453 | 0.016 | 0.885 | protein (peptidyl-prolyl cis/trans isomerase) NIMA-interacting 1 | gi|12963653 | Q9QUR7 | Pin1 | Cell growth and death | E | 21 | 2 | 165 | 18359 | 8.93 |
| **1C** | BID835 | 0.019 | 0.868 | inorganic pyrophosphatase 2 | gi|22203753 | Q91VM9 | Ppa2 | Energy Metabolism | E | 5 | 2 | 105 | 38090 | 6.51 |
| **1C** | SID874 | 0.010 | 0.905 | Protein phosphatase 1 regulatory subunit 7 | gi|12963569 | Q3UM45 | Ppp1r7 | Signal transduction | E | 28 | 9 | 559 | 41266 | 4.85 |
| **1C** | BID642 | 0.040 | 0.876 | phosphoserine aminotransferase 1 | GI:26354941 | Q8BTJ1 | Psat1 | Cofactor Metabolism | E | 12 | 4 | 257 | 40448 | 7.01 |
| **1C** | SID734 | 0.021 | 0.958 | 26S proteasome non-ATPase regulatory subunit 4 | gi|6679505 | O35226 | Psmd4 | Degradation | E | 23 | 7 | 455 | 40678 | 4.67 |
| **1C** | SID964 | 0.001 | 1.098 | Transcriptional activator protein Pur-alpha | gi|34878862 | P42669 | Pura | Cell growth and death | M | 11 | 4 | 205 | 34862 | 6.07 |
| **1C** | SID15215 | 0.024 | 0.926 | Transcriptional activator protein Pur-beta | gi|6755252 | O35295 | Purb | Transcription | E | 12 | 2 | 153 | 33881 | 5.35 |
| **1C** | SID15225 | 0.005 | 1.052 | Transcriptional activator protein Pur-beta | gi|6755252 | O35295 | Purb | Transcription | E | 12 | 3 | 164 | 33881 | 5.35 |
| **1C** | SID15796 | 0.007 | 0.924 | Parvalbumin alpha | gi|53819 | P32848 | Pvalb | Signal transduction | E | 52 | 5 | 279 | 11937 | 5.02 |
| **1C** | BID1099 | 0.023 | 1.048 | quininoid dihydropteridine reductase | gi|21312520 | Q8BVI4 | Qdpr | Cofactor Metabolism | E | 28 | 5 | 310 | 25554 | 7.67 |
| **1C** | BID523 | 0.047 | 0.942 | Septin-7 | gi|28173550 | O55131 | Sept7 | Cytoskeleton | E | 36 | 14 | 882 | 50617 | 8.73 |
| **1C** | BID408 | 0.026 | 1.115 | Plasminogen activator inhibitor 1 RNA-binding protein | gi|12846595 | Q9CY58 | Serbp1 | Translation | E | 15 | 4 | 230 | 42918 | 8.42 |
| **1C** | SID1013 | 0.027 | 0.908 | Serine (Or cysteine) proteinase inhibitor, clade B (Ovalbumin), member 1a | gi|56206897 | Q5SUV7 | Serpinb1a |  | E | 5 | 2 | 116 | 42573 | 6.22 |
| **1C** | BID764 | 0.046 | 1.164 | sideroflexin 5 | gi|13785620 | Q925N0 | Sfxn5 |  | E | 10 | 2 | 113 | 19955 | 9.67 |
| **1C** | SID15198 | 0.004 | 1.050 | SH3 domain protein 2A | gi|31560792 | Q8VBV1 | Sh3gl2 |  | E | 39 | 12 | 682 | 39930 | 5.26 |
| **1C** | SID678 | 0.037 | 0.889 | SH3 domain GRB2-like protein B2 | gi|21314838 | Q8R3V5 | Sh3glb2 |  | E | 39 | 14 | 784 | 44089 | 5.73 |
| **1C** | SID15696 | 0.014 | 1.075 | synuclein, alpha | gi|6678047 | O55042 | Snca |  | M | 45 | 8 | 99 | 14476 | 4.74 |
| **1C** | SID15685 | 0.024 | 1.044 | synuclein, alpha | gi|6678047 | O55042 | Snca |  | M | 45 | 8 | 99 | 14476 | 4.74 |
| **1C** | BID1056 | 0.044 | 0.842 | U2 small nuclear ribonucleoprotein A' | gi|7021537 | P57784 | Snrpa1 |  | E | 10 | 2 | 137 | 28211 | 8.73 |
| **1C** | SID1052 | 0.023 | 0.797 | Serine racemase | gi|26389300 | Q9QZX7 | Srr | AS Metabolism | E | 19 | 4 | 242 | 36340 | 5.68 |
| **1C** | BID229 | 0.016 | 1.242 | Synapsin-2 | gi|42406392 | Q64332 | Syn2 | Neuron | E | 13 | 6 | 347 | 63202 | 8.59 |
| **1C** | BID1567 | 0.050 | 0.725 | thioesterase superfamily member 2 | gi|13385260 | Q9CQR4 | Them2 |  | E | 15 | 2 | 136 | 15173 | 8.95 |
| **1C** | BID1193 | 0.033 | 1.110 | triosephosphate isomerase | gi|1864018 | Q64513 | Tpi1 | CH Metabolism | E | 33 | 6 | 357 | 22492 | 5.62 |
| **1C** | SID4172 | 0.028 | 0.918 | Ubiquilin-2 | gi|34328236 | Q9QZM0 | Ubqln2 | Degradation | E | 12 | 5 | 280 | 67308 | 5.16 |
| **1C** | SID446 | 0.030 | 0.921 | Ubiquilin-2 | gi|6014493 | Q9QZM0 | Ubqln2 | Degradation | E | 12 | 5 | 280 | 67308 | 5.16 |
| **1C** | SID4796 | 0.049 | 1.214 | ubiquitin carboxyl-terminal esterase L3 | gi|7710106 | Q9JKB1 | Uchl3 | Degradation | E | 36 | 6 | 415 | 26162 | 5.08 |
| **1C** | SID632 | 0.006 | 0.867 | Transitional endoplasmic reticulum ATPase | gi|400712 | Q01853 | Vcp | Degradation | E | 1 | 1 | 52 | 89252 | 5.14 |
| **1C** | SID1310 | 0.038 | 0.858 | WW domain binding protein 2 | gi|8394539 | P97765 | Wbp2 |  | E | 5 | 1 | 70 | 28013 | 5.94 |
| **1C** | SID1294 | 0.010 | 1.050 | 14-3-3 protein gamma | gi|3065929 | P61982 | Ywhag | Cell growth and death | E | 48 | 20 | 114 | 28285 | 4.8 |
| **1H** | BID1104 | 0.005 | 0.917 | adenylate kinase 2 isoform b | gi|34328230 | Q8C7I9 | Ak2 | Nucleotide metabolism | E | 4 | 1 | 42 | 25589 | 6.97 |
| **1H** | BID660 | 0.050 | 1.087 | aldolase 1, A isoform | gi|6671539 | Q5FWB7 | Aldoa | CH Metabolism | E | 16 | 7 | 444 | 39331 | 8.31 |
| **1H** | BID690 | 0.017 | 1.085 | aldolase 1, A isoform | gi|6671539 | Q5FWB7 | Aldoa | CH Metabolism | E | 23 | 8 | 464 | 39331 | 8.31 |
| **1H** | SID2679 | 0.027 | 1.067 | Apolipoprotein E | gi|74178217 | P08226 | Apoe | Lipid metabolism | E | 26 | 9 | 515 | 35884 | 5.56 |
| **1H** | SID2064 | 0.039 | 0.871 | ATP synthase subunit beta, mitochondrial [Precursor] | gi|2623222 | P56480 | Atp5b | Energy Metabolism | E | 17 | 6 | 337 | 56632 | 5.24 |
| **1H** | SID15757 | 0.027 | 1.102 | ATP synthase delta chain, mitochondrial [Precursor] | gi|12847456 | Q9D3D9 | Atp5d | Energy Metabolism | E | 17 | 4 | 236 | 17619 | 5.03 |
| **1H** | SID663 | 0.001 | 1.028 | chaperonin subunit 5 (epsilon) | gi|6671702 | P80316 | Cct5 | Folding | E | 38 | 16 | 1116 | 59586 | 5.72 |
| **1H** | SID15241 | 0.027 | 0.859 | Clathrin light chain B (Lcb) | gi|30794164 | Q6IRU5 | Cltb | Sorting | E | 13 | 3 | 167 | 23160 | 4.63 |
| **1H** | BID1723 | 0.014 | 1.055 | diazepam binding inhibitor isoform 2 | gi|6681137 | P31786 | Dbi | Lipid metabolism | E | 32 | 2 | 77 | 9994 | 8.78 |
| **1H** | SID507 | 0.002 | 1.089 | Dihydropyrimidinase-related protein 2 | gi|1915913 | O08553 | Dpysl2 | Neuron | E | 7 | 3 | 183 | 62132 | 5.95 |
| **1H** | SID5670 | 0.007 | 1.085 | Dihydropyrimidinase-related protein 2 | gi|40254595 | O08553 | Dpysl2 | Neuron | M | 23 | 17 | 142 | 62239 | 5.95 |
| **1H** | BID529 | 0.020 | 1.078 | enolase 1, alpha non-neuron | gi|12963491 | Q5FW97 | Eno1 | CH Metabolism | E | 14 | 6 | 320 | 47095 | 6.37 |
| **1H** | BID525 | 0.014 | 1.062 | enolase 1, alpha non-neuron | gi|53734652 | Q5XJG8 | Eno1 | CH Metabolism | E | 19 | 7 | 421 | 50209 | 8.01 |
| **1H** | SID1383 | 0.006 | 0.893 | enolase 2, gamma neuronal | gi|7305027 | Q922A0 | Eno2 | CH Metabolism | E | 4 | 2 | 91 | 47267 | 4.99 |
| **1H** | SID1724 | 0.018 | 0.902 | Fatty acid-binding protein, epidermal | gi|6754450 | Q05816 | Fabp5 | Lipid metabolism | E | 32 | 4 | 236 | 15127 | 6.14 |
| **1H** | BID412 | 0.015 | 0.865 | Fascin (Singed-like protein) | gi|2498358 | Q61553 | Fscn1 | Cytoskeleton | E | 12 | 6 | 300 | 54371 | 6.21 |
| **1H** | SID15193 | 0.033 | 0.921 | Neuromodulin | gi|20271449 | P06837 | Gap43 | Neuron | E | 50 | 8 | 582 | 23618 | 4.63 |
| **1H** | BID783 | 0.018 | 1.096 | similar to glyceraldehyde-3-phosphate dehydrogenase | gi|6679937 | P16858 | Gapdh | CH Metabolism | E | 21 | 5 | 355 | 35787 | 8.44 |
| **1H** | BID784 | 0.015 | 1.081 | similar to glyceraldehyde-3-phosphate dehydrogenase | gi|6679937 | P16858 | Gapdh | CH Metabolism | E | 31 | 9 | 626 | 35787 | 8.44 |
| **1H** | SID576 | 0.013 | 0.935 | guanosine diphosphate dissociation inhibitor 1 | gi|18139889 | P50396 | Gdi1 | Signal transduction | E | 35 | 12 | 786 | 50479 | 4.96 |
| **1H** | SID5893 | 0.028 | 0.642 | glial fibrillary acidic protein | gi|51066 | P03995 | Gfap | Cytoskeleton | E | 7 | 3 | 161 | 48437 | 5.29 |
| **1H** | SID1256 | 0.048 | 0.942 | Glod4 protein | gi|12840311 | Q9CPV4 | Glod4 | Metabolism | E | 45 | 12 | 776 | 33296 | 5.28 |
| **1H** | SID15240 | 0.004 | 0.891 | guanine nucleotide-binding protein, beta-1 subunit | gi|6680045 | P62874 | Gnb1 | Signal transduction | E | 9 | 3 | 175 | 37353 | 5.96 |
| **1H** | BID721 | 0.025 | 0.914 | mitochondrial aspartate aminotransferase | gi|6754036 | P05202 | Got2 | AS Metabolism | E | 37 | 17 | 1047 | 47381 | 9.13 |
| **1H** | BID1132 | 0.048 | 1.137 | glutathione S-transferase, mu 1 | gi|6754084 | Q58ET5 | Gstm1 | AS Metabolism | E | 55 | 13 | 724 | 25953 | 7.71 |
| **1H** | BID1632 | 0.026 | 1.304 | alpha-globin | gi|193761 | Q61649 | Hba-a1 |  | E | 44 | 2 | 87 | 6212 | 6.82 |
| **1H** | BID206 | 0.003 | 0.895 | hydroxysteroid (17-beta) dehydrogenase 4 | gi|31982273 | P51660 | Hsd17b4 | Lipid metabolism | E | 6 | 4 | 245 | 79432 | 8.76 |
| **1H** | SID530 | 0.041 | 1.106 | heat shock protein 2 [Mus musculus] | gi|31560686 | P17156 | Hspa9 | Folding | E | 45 | 28 | 1661 | 69599 | 5.51 |
| **1H** | BID549 | 0.045 | 1.028 | mitogen activated protein kinase kinase 1 | gi|6678794 | P31938 | Map2k1 | Signal transduction | E | 10 | 4 | 233 | 43446 | 6.24 |
| **1H** | SID1242 | 0.005 | 0.932 | Microtubule-associated protein RP/EB family member 1 | gi|7106301 | Q61166 | Mapre1 | Cytoskeleton | E | 14 | 2 | 135 | 29997 | 5.12 |
| **1H** | SID1186 | 0.023 | 0.941 | Malate dehydrogenase, cytoplasmic | gi|387129 | P14152 | Mdh1 | CH Metabolism | E | 15 | 5 | 279 | 36454 | 6.16 |
| **1H** | SID482 | 0.021 | 1.478 | NADH dehydrogenase (ubiquinone) Fe-S protein 1 | gi|21704020 | Q91VD9 | Ndufs1 | Energy Metabolism | E | 5 | 3 | 184 | 79698 | 5.51 |
| **1H** | BID1667 | 0.009 | 0.828 | NADH dehydrogenase (ubiquinone) Fe-S protein 6 | gi|56711244 | P52503 | Ndufs6 | Energy Metabolism | E | 33 | 4 | 272 | 13012 | 8.87 |
| **1H** | BID1180 | 0.032 | 0.952 | Protein-L-isoaspartate(D-aspartate) O-methyltransferase | gi|417489 | P23506 | Pcmt1 | Protein modification | E | 9 | 2 | 130 | 24619 | 7.1 |
| **1H** | SID1118 | 0.043 | 0.800 | pyruvate dehydrogenase (lipoamide) beta | gi|18152793 | Q9D051 | Pdhb | CH Metabolism | E | 6 | 2 | 124 | 38912 | 6.41 |
| **1H** | BID197 | 0.022 | 0.906 | 6-phosphofructokinase, muscle type (Phosphofructokinase 1) (Phosphohexokinase) (Phosphofructo-1-kin | gi|13638207 | P47857 | Pfkm | CH Metabolism | E | 18 | 13 | 735 | 85215 | 8.24 |
| **1H** | BID1439 | 0.029 | 1.050 | peptidylprolyl isomerase A | gi|6679439 | P17742 | Ppia | Folding | E | 37 | 7 | 384 | 17960 | 7.74 |
| **1H** | BID1458 | 0.035 | 1.075 | Peptidylprolyl isomerase A | gi|12846244 | P17742 | Ppia | Folding | E | 28 | 4 | 266 | 17960 | 7.74 |
| **1H** | BID1274 | 0.019 | 0.933 | peroxiredoxin 1 | gi|6754976 | P35700 | Prdx1 | Degradation | E | 20 | 5 | 224 | 22162 | 8.26 |
| **1H** | BID1260 | 0.026 | 1.339 | prion interactor PINT1 | gi|14581470 | Q923K5 | Prnpip1 |  | E | 7 | 1 | 46 | 14033 | 5.11 |
| **1H** | SID1301 | 0.026 | 0.952 | Proteasome subunit alpha type 3 | gi|31981534 | O70435 | Psma3 | Degradation | E | 25 | 7 | 350 | 28387 | 5.29 |
| **1H** | SID1375 | 0.030 | 0.969 | proteasome (prosome, macropain) subunit, alpha type 5 | gi|7106387 | Q5E987 | Psma5 | Degradation | E | 17 | 3 | 216 | 26394 | 4.74 |
| **1H** | BID644 | 0.033 | 1.051 | Transcriptional activator protein Pur-alpha | gi|6679573 | P42669 | Pura | Cell growth and death | E | 11 | 4 | 205 | 34862 | 6.07 |
| **1H** | BID1828 | 0.031 | 0.806 | Septin-11 | gi|26324430 | Q8C1B7 | Sept11 | Cytoskeleton | E | 17 | 6 | 388 | 49649 | 8.73 |
| **1H** | BID678 | 0.000 | 1.061 | Neuronal-specific septin-3 | gi|13124538 | Q9Z1S5 | Sept3 | Cytoskeleton | E | 4 | 2 | 99 | 52756 | 7.45 |
| **1H** | BID516 | 0.028 | 0.856 | Septin-7 | gi|28173550 | O55131 | Sept7 | Cytoskeleton | E | 25 | 11 | 639 | 50617 | 8.73 |
| **1H** | BID515 | 0.034 | 0.865 | splicing factor 3b, subunit 4 | gi|23346437 | Q8QZY9 | Sf3b4 | Translation | E | 3 | 1 | 71 | 44327 | 8.54 |
| **1H** | SID15685 | 0.048 | 1.119 | synuclein, alpha | gi|6678047 | O55042 | Snca |  | M | 45 | 8 | 99 | 14476 | 4.74 |
| **1H** | SID15696 | 0.047 | 1.159 | synuclein, alpha | gi|6678047 | O55042 | Snca |  | M | 45 | 8 | 99 | 14476 | 4.74 |
| **1H** | SID1158 | 0.044 | 0.871 | spermidine synthase | gi|6678131 | Q64674 | Srm | AS Metabolism | E | 9 | 2 | 144 | 33973 | 5.31 |
| **1H** | BID221 | 0.016 | 2.190 | Synapsin-2 | gi|42406392 | Q64332 | Syn2 | Neuron | E | 10 | 5 | 332 | 63202 | 8.59 |
| **1H** | BID229 | 0.008 | 1.370 | Synapsin-2 | gi|42406392 | Q64332 | Syn2 | Neuron | E | 13 | 6 | 347 | 63202 | 8.59 |
| **1H** | BID1158 | 0.035 | 1.053 | triosephosphate isomerase | gi|1864018 | P17751 | Tpi1 | CH Metabolism | E | 41 | 9 | 534 | 22492 | 5.62 |
| **1H** | BID1193 | 0.009 | 1.142 | triosephosphate isomerase | gi|1864018 | Q64513 | Tpi1 | CH Metabolism | E | 33 | 6 | 357 | 22492 | 5.62 |
| **1H** | SID1391 | 0.042 | 0.889 | Translationally-controlled tumor protein | gi|6678437 | P63028 | Tpt1 | Signal transduction | E | 26 | 5 | 274 | 19450 | 5.62 |
| **1H** | SID1395 | 0.025 | 0.947 | Translationally-controlled tumor protein | gi|6678437 | P63028 | Tpt1 | Signal transduction | E | 33 | 6 | 307 | 19450 | 5.62 |
| **1H** | SID15814 | 0.006 | 0.925 | Thioredoxin | gi|14789654 | P10639 | Txn | Energy Metabolism | E | 38 | 4 | 251 | 11668 | 4.8 |
| **1H** | SID1049 | 0.021 | 0.921 | Thioredoxin-like protein 1 | gi|31543902 | Q8CDN6 | Txnl1 | Energy Metabolism | M | 41 | 8 | 143 | 32217 | 4.84 |
| **2C** | BID1875 | 0.019 | 0.923 | 4-aminobutyrate aminotransferase | gi|37202121 | P61922 | Abat | AS Metabolism | E | 22 | 9 | 586 | 50205 | 8.79 |
| **2C** | BID709 | 0.021 | 1.154 | Acetyl-CoA acetyltransferase, cytosolic | gi|13435978 | Q8CAY6 | Acat2 | AS Metabolism | E | 7 | 2 | 117 | 38123 | 7.63 |
| **2C** | SID15765 | 0.049 | 0.786 | alpha-cardiac actin | gi|387090 | P68033 | Actc1 | Cytoskeleton | E | 5 | 2 | 113 | 41758 | 5.23 |
| **2C** | SID2679 | 0.014 | 1.261 | Apolipoprotein E | gi|74178217 | P08226 | Apoe | Lipid metabolism | E | 26 | 9 | 515 | 35884 | 5.56 |
| **2C** | SID1437 | 0.049 | 1.137 | ATP synthase D chain, mitochondrial | gi|21313679 | Q9DCX2 | Atp5h | Energy Metabolism | E | 40 | 6 | 363 | 18738 | 5.52 |
| **2C** | SID15879 | 0.023 | 1.118 | ATP synthase coupling factor 6, mitochondrial [Precursor] | gi|7949005 | P97450 | Atp5j | Energy Metabolism | E | 46 | 4 | 220 | 12489 | 9.36 |
| **2C** | BID1084 | 0.035 | 0.933 | Methylglutaconyl-CoA hydratase, mitochondrial [Precursor] | gi|20072952 | Q9JLZ3 | Auh | AS Metabolism | E | 34 | 10 | 539 | 32621 | 9.57 |
| **2C** | SID792 | 0.019 | 0.920 | brain abundant, membrane attached signal protein 1 | gi|45598372 | Q91XV3 | Basp1 | Neuron | E | 51 | 7 | 494 | 22074 | 4.5 |
| **2C** | SID451 | 0.003 | 1.104 | Myc box-dependent-interacting protein 1 | gi|6753050 | O08539 | Bin1 | Cell growth and death | E | 8 | 3 | 181 | 64430 | 4.95 |
| **2C** | SID457 | 0.022 | 1.048 | Myc box-dependent-interacting protein 1 | gi|6753050 | O08539 | Bin1 | Cell growth and death | E | 10 | 4 | 233 | 64430 | 4.95 |
| **2C** | BID1383 | 0.012 | 0.868 | cofilin 1, non-muscle | gi|6680924 | P18760 | Cfl1 | Cytoskeleton | E | 33 | 4 | 270 | 18548 | 8.22 |
| **2C** | BID1868 | 0.012 | 1.278 | suppressor of K+ transport defect 3 | gi|6677983 | Q60649 | Clpb | Transcription | E | 15 | 8 | 474 | 75956 | 8.68 |
| **2C** | SID1207 | 0.022 | 0.826 | Clathrin light chain B (Lcb) | gi|26337755 | Q6IRU5 | Cltb | Sorting | E | 12 | 2 | 87 | 16824 | 4.37 |
| **2C** | SID15750 | 0.038 | 0.778 | Complexin-1 | gi|62471462 | P63040 | Cplx1 | Sorting | E | 38 | 4 | 323 | 15112 | 4.93 |
| **2C** | SID15715 | 0.030 | 1.264 | Complexin-2 | gi|5729783 | P84086 | Cplx2 | Sorting | E | 11 | 1 | 59 | 15385 | 5.06 |
| **2C** | BID190 | 0.025 | 0.851 | ATP-dependent RNA helicase DDX3X | gi|6753620 | Q62167 | Ddx3x | Translation | E | 17 | 11 | 608 | 73056 | 6.73 |
| **2C** | BID311 | 0.022 | 0.866 | Probable ATP-dependent RNA helicase DDX5 (DEAD box protein 5) (RNA helicase p68) (DEAD box RNA heli | gi|2500527 | Q61656 | Ddx5 | Transcription | E | 5 | 3 | 158 | 69277 | 9.06 |
| **2C** | SID449 | 0.043 | 0.812 | postsynaptic density protein 95 | gi|6681195 | Q62108 | Dlg4 | Neuron | E | 2 | 1 | 52 | 26390 | 5.56 |
| **2C** | SID371 | 0.009 | 1.179 | Dynamin-1 | gi|32172431 | P39053 | Dnm1 | Cytoskeleton | E | 4 | 4 | 217 | 97741 | 7.61 |
| **2C** | SID378 | 0.015 | 1.120 | Dynamin-1 | gi|32172431 | P39053 | Dnm1 | Cytoskeleton | E | 14 | 11 | 689 | 97741 | 5.97 |
| **2C** | SID507 | 0.045 | 1.206 | Dihydropyrimidinase-related protein 2 | gi|1915913 | O08553 | Dpysl2 | Neuron | E | 7 | 3 | 183 | 62132 | 5.95 |
| **2C** | SID536 | 0.021 | 1.077 | Dihydropyrimidinase-related protein 2 | gi|1915913 | O08553 | Dpysl2 | Neuron | E | 9 | 3 | 219 | 62132 | 5.95 |
| **2C** | SID5670 | 0.041 | 1.087 | Dihydropyrimidinase-related protein 2 | gi|40254595 | O08553 | Dpysl2 | Neuron | M | 23 | 17 | 142 | 62239 | 5.95 |
| **2C** | SID2333 | 0.010 | 0.932 | Dual specificity protein phosphatase 3 | gi|21312314 | Q9D7X3 | Dusp3 | Signal transduction | E | 13 | 2 | 116 | 20459 | 6.07 |
| **2C** | SID439 | 0.007 | 1.160 | cytoplasmic dynein intermediate chain 1B | gi|3493589 | O88485 | Dync1i1 | Sorting | E | 3 | 1 | 96 | 68422 | 5.13 |
| **2C** | SID15301 | 0.001 | 1.215 | EF-hand domain-containing protein 2 | gi|12847201 | Q9D8Y0 | Efhd2 | Translation | E | 4 | 1 | 52 | 25084 | 4.98 |
| **2C** | SID15303 | 0.015 | 0.771 | EF-hand domain-containing protein 2 | gi|12847201 | Q9D8Y0 | Efhd2 | Translation | E | 16 | 3 | 191 | 25084 | 4.98 |
| **2C** | SID15307 | 0.013 | 1.575 | EF-hand domain-containing protein 2 | gi|12847201 | Q9D8Y0 | Efhd2 | Translation | E | 5 | 1 | 58 | 25084 | 4.98 |
| **2C** | SID15308 | 0.024 | 0.771 | EF-hand domain-containing protein 2 | gi|12847201 | Q9D8Y0 | Efhd2 | Translation | E | 39 | 8 | 500 | 25084 | 4.98 |
| **2C** | SID406 | 0.014 | 0.421 | epsin 1 | gi|46195711 | Q80VP1 | Epn1 | Lipid metabolism | E | 7 | 3 | 194 | 60175 | 4.69 |
| **2C** | SID424 | 0.006 | 0.491 | epsin 1 | gi|46195711 | Q80VP1 | Epn1 | Lipid metabolism | E | 17 | 7 | 413 | 60175 | 4.69 |
| **2C** | SID873 | 0.021 | 0.900 | Neuromodulin | gi|6679935 | P06837 | Gap43 | Neuron | E | 50 | 8 | 582 | 23618 | 4.63 |
| **2C** | SID1255 | 0.034 | 0.814 | Glod4 protein | gi|12840311 | Q9CPV4 | Glod4 | Metabolism | E | 7 | 2 | 105 | 30765 | 4.8 |
| **2C** | SID1062 | 0.009 | 0.947 | guanine nucleotide-binding protein, beta-1 subunit | gi|6680045 | P62874 | Gnb1 | Signal transduction | E | 25 | 8 | 532 | 37353 | 5.6 |
| **2C** | SID5633 | 0.046 | 0.835 | Gelsolin [Precursor] | gi|18606238 | P13020 | Gsn | Cytoskeleton | E | 20 | 12 | 774 | 80712 | 5.52 |
| **2C** | BID230 | 0.006 | 0.901 | Hydroxyacyl-Coenzyme A dehydrogenase/3-ketoacyl-Coenzyme A thiolase/enoyl-Coenzyme A hydratase (Trifunctional protein), alpha subunit | gi|33859811 | Q5U5Y5 | Hadha | Lipid metabolism | E | 13 | 7 | 483 | 82618 | 9.24 |
| **2C** | BID234 | 0.020 | 0.865 | Hydroxyacyl-Coenzyme A dehydrogenase/3-ketoacyl-Coenzyme A thiolase/enoyl-Coenzyme A hydratase (Trifunctional protein), alpha subunit | gi|33859811 | Q5U5Y5 | Hadha | Lipid metabolism | E | 19 | 12 | 770 | 82618 | 9.24 |
| **2C** | SID1216 | 0.033 | 0.889 | haloacid dehalogenase-like hydrolase domain containing 2 isoform 1 | gi|12833114 | Q3UGR5 | Hdhd2 | Metabolism | E | 18 | 3 | 322 | 26635 | 5.81 |
| **2C** | BID532 | 0.020 | 0.933 | Heterogeneous nuclear ribonucleoprotein A3 | gi|94388210 | Q8BG05 | Hnrnpa3 | Translation | E | 17 | 4 | 263 | 37185 | 8.46 |
| **2C** | SID680 | 0.045 | 1.081 | heat shock protein 65 | gi|51455 | P63038 | Hspd1 | Folding | E | 41 | 19 | 1434 | 60903 | 5.91 |
| **2C** | SID1063 | 0.042 | 1.078 | isocitrate dehydrogenase 3 (NAD+) alpha | gi|18250284 | Q9D6R2 | Idh3a | CH Metabolism | E | 7 | 3 | 177 | 39613 | 6.27 |
| **2C** | SID420 | 0.002 | 1.069 | Mitochondrial inner membrane protein | gi|26339872 | Q8CAQ8 | Immt |  | E | 21 | 14 | 757 | 82878 | 6.18 |
| **2C** | BID208 | 0.006 | 1.725 | KH domain-containing, RNA-binding, signal transduction-associated protein 1 | gi|602251 | Q60749 | Khdrbs1 | Transcription | E | 2 | 1 | 43 | 48456 | 8.92 |
| **2C** | BID219 | 0.009 | 1.436 | KH domain-containing, RNA-binding, signal transduction-associated protein 1 | gi|602251 | Q60749 | Khdrbs1 | Transcription | E | 11 | 4 | 227 | 48455 | 8.92 |
| **2C** | BID871 | 0.033 | 0.907 | Malate dehydrogenase, mitochondrial precursor | gi|126897 | P08249 | Mdh2 | CH Metabolism | E | 11 | 3 | 150 | 35574 | 8.83 |
| **2C** | SID15643 | 0.000 | 0.757 | 39S ribosomal protein L12, mitochondrial [Precursor] | gi|12837801 | Q9DB15 | Mrpl12 | Translation | E | 30 | 7 | 348 | 21695 | 9.34 |
| **2C** | SID15744 | 0.002 | 1.134 | Myosin light polypeptide 6 | gi|17986258 | Q60605 | Myl6 | Cytoskeleton | E | 31 | 4 | 277 | 16919 | 4.56 |
| **2C** | BID738 | 0.015 | 0.891 | Poly(rC)-binding protein 2 | gi|495128 | Q61990 | Pcbp2 | Transcription | E | 20 | 5 | 294 | 34994 | 8.48 |
| **2C** | BID1832 | 0.013 | 0.853 | pyruvate dehydrogenase E1 alpha 1 | gi|6679261 | P35486 | Pdha1 | CH Metabolism | M | 48 | 23 | 130 | 43204 | 8.49 |
| **2C** | SID15784 | 0.024 | 0.762 | Astrocytic phosphoprotein PEA-15 | gi|21426847 | Q62048 | Pea15 | Signal transduction | E | 7 | 1 | 58 | 15045 | 4.94 |
| **2C** | SID15785 | 0.024 | 0.785 | Astrocytic phosphoprotein PEA-15 | gi|23398567 | Q62048 | Pea15 | Signal transduction | E | 7 | 1 | 58 | 15045 | 4.94 |
| **2C** | SID15777 | 0.003 | 1.941 | Astrocytic phosphoprotein PEA-15 | gi|21426847 | Q62048 | Pea15 | Signal transduction | E | 7 | 1 | 89 | 15045 | 4.94 |
| **2C** | SID15624 | 0.049 | 1.222 | Phosphatidylethanolamine-binding protein 1 | gi|1517864 | P70296 | Pebp1 | Lipid metabolism | E | 6 | 1 | 75 | 20847 | 5.19 |
| **2C** | BID595 | 0.005 | 0.930 | Phosphoglycerate kinase 1 | gi|129903 | P09411 | Pgk1 | CH Metabolism | E | 34 | 10 | 681 | 44508 | 7.53 |
| **2C** | BID601 | 0.003 | 0.630 | guanine nucleotide exchange factor for ADP ribosylation factor 6 isoform 1 | gi|83921581 | Q8CEA6 | Psd3 | Signal transduction | E | 15 | 8 | 425 | 58430 | 8.92 |
| **2C** | BID591 | 0.050 | 0.784 | SEC14-like protein 2 | gi|21362309 | Q99J08 | Sec14l2 | Lipid metabolism | E | 13 | 4 | 201 | 46271 | 6.69 |
| **2C** | BID1828 | 0.004 | 0.845 | Septin-11 | gi|26324430 | Q8C1B7 | Sept11 | Cytoskeleton | E | 17 | 6 | 388 | 49649 | 6.14 |
| **2C** | SID1013 | 0.047 | 0.877 | Serine (Or cysteine) proteinase inhibitor, clade B (Ovalbumin), member 1a | gi|56206897 | Q5SUV7 | Serpinb1a |  | E | 5 | 2 | 116 | 42573 | 6.22 |
| **2C** | BID414 | 0.010 | 0.662 | Signal recognition particle 54 kDa protein | gi|54194 | P14576 | Srp54 | Sorting | E | 22 | 9 | 643 | 55709 | 8.96 |
| **2C** | SID15699 | 0.006 | 0.794 | Stathmin | gi|9789995 | P54227 | Stmn1 | Signal transduction | E | 29 | 5 | 234 | 17264 | 5.76 |
| **2C** | BID253 | 0.010 | 1.095 | synapsin I | gi|1041085 | O88935 | Syn1 | Neuron | E | 18 | 1 | 104 | 12232 | 8.31 |
| **2C** | BID200 | 0.005 | 1.728 | synapsin Ib | gi|3599473 | O88935 | SynI | Neuron | E | 11 | 6 | 402 | 69992 | 9.84 |
| **2C** | BID201 | 0.013 | 1.419 | synapsin Ib | gi|3599473 | O88935 | SynI | Neuron | E | 9 | 4 | 273 | 69992 | 9.84 |
| **2C** | BID211 | 0.002 | 1.487 | synapsin Ib | gi|3599473 | O88935 | SynI | Neuron | E | 9 | 4 | 269 | 69991 | 9.84 |
| **2C** | BID215 | 0.007 | 0.763 | synapsin Ib | gi|3599473 | O88935 | SynI | Neuron | E | 25 | 12 | 781 | 69991 | 9.84 |
| **2C** | BID222 | 0.024 | 0.814 | synapsin Ib | gi|3599473 | O88935 | SynI | Neuron | E | 25 | 12 | 701 | 69992 | 9.84 |
| **2C** | SID15214 | 0.046 | 0.963 | Tropomodulin-2 | gi|6934242 | Q9JKK7 | Tmod2 | Cytoskeleton | E | 29 | 9 | 474 | 39547 | 5.28 |
| **2C** | BID919 | 0.042 | 0.885 | Mitochondrial import receptor subunit TOM34 (Translocase of outer membrane 34 kDa subunit) | gi|24212072 | Q9CYG7 | Tomm34 | Folding | E | 22 | 6 | 340 | 34257 | 9.24 |
| **2C** | SID15319 | 0.020 | 0.953 | Tropomyosin 1 alpha chain (Alpha-tropomyosin) | gi|20522240 | Q564G1 | Tpm1 | Cytoskeleton | E | 25 | 8 | 383 | 32689 | 4.71 |
| **2C** | BID1889 | 0.017 | 0.812 | UBX domain-containing protein 1 | gi|13277354 | Q99PL6 | Ubxd1 |  | E | 13 | 4 | 258 | 49765 | 8.7 |
| **2H** | BID1875 | 0.048 | 0.870 | 4-aminobutyrate aminotransferase | gi|37202121 | P61922 | Abat | AS Metabolism | E | 22 | 9 | 586 | 50205 | 8.79 |
| **2H** | BID573 | 0.016 | 0.828 | 3-ketoacyl-CoA thiolase, mitochondrial | gi|29126205 | Q8BWT1 | Acaa2 | Lipid metabolism | E | 14 | 4 | 232 | 41803 | 8.33 |
| **2H** | SID927 | 0.000 | 0.922 | Actin, cytoplasmic 1 | gi|49868 | P60710 | Actb | Cytoskeleton | E | 14 | 4 | 251 | 39161 | 5.78 |
| **2H** | SID15765 | 0.017 | 0.847 | alpha-cardiac actin | gi|387090 | P68033 | Actc1 | Cytoskeleton | E | 5 | 2 | 113 | 41758 | 5.23 |
| **2H** | SID15213 | 0.007 | 0.959 | gamma-actin | gi|809561 | P63260 | Actg1 | Cytoskeleton | E | 22 | 8 | 462 | 40992 | 5.56 |
| **2H** | SID897 | 0.028 | 0.944 | gamma-actin | gi|809561 | P63260 | Actg1 | Cytoskeleton | E | 38 | 12 | 708 | 40992 | 5.56 |
| **2H** | SID910 | 0.036 | 0.938 | gamma-actin | gi|809561 | P63260 | Actg1 | Cytoskeleton | E | 27 | 9 | 487 | 40992 | 5.56 |
| **2H** | BID419 | 0.038 | 0.967 | aldehyde dehydrogenase family 6, subfamily A1 | gi|19527258 | Q1LZK2 | Aldh6a1 | AS Metabolism | E | 22 | 9 | 591 | 57878 | 8.29 |
| **2H** | BID650 | 0.036 | 1.115 | aldolase 1, A isoform | gi|6671539 | Q5FWB7 | Aldoa | CH Metabolism | E | 52 | 19 | 1148 | 39331 | 8.31 |
| **2H** | SID15202 | 0.001 | 0.858 | apolipoprotein A-IV precursor - mouse | gi|91885 | Q01488 | Apoa4 | Lipid metabolism | E | 15 | 4 | 274 | 45455 | 5.77 |
| **2H** | SID985 | 0.016 | 0.905 | ADP-ribosylation factor interacting protein 2 | gi|31981363 | Q8K221 | Arfip2 | Signal transduction | E | 12 | 3 | 233 | 37776 | 5.72 |
| **2H** | SID5668 | 0.003 | 1.136 | ATPase, H+ transporting, V1 subunit A, isoform 1 | gi|31560731 | P50516 | Atp6v1a | Energy Metabolism | E | 34 | 16 | 1122 | 68283 | 5.42 |
| **2H** | SID729 | 0.001 | 1.103 | vacuolar H+ATPase B2 | gi|17105370 | P62814 | Atp6v1b2 | Energy Metabolism | E | 36 | 18 | 1112 | 56515 | 5.57 |
| **2H** | SID746 | 0.014 | 0.860 | vacuolar H+ATPase B2 | gi|17105370 | P62814 | Atp6v1b2 | Energy Metabolism | E | 11 | 5 | 314 | 56515 | 5.57 |
| **2H** | SID15671 | 0.041 | 3.282 | hypothetical protein LOC68165 | gi|12845489 | Q9CV00 | B230118G17Rik | Energy Metabolism | E | 14 | 2 | 128 | 18107 | 5.51 |
| **2H** | SID918 | 0.014 | 0.915 | bisphosphate 3'-nucleotidase 1 | gi|15029655 | Q3U449 | Bpnt1 | Energy Metabolism | M | 28 | 7 | 76 | 33000 | 5.58 |
| **2H** | SID1358 | 0.046 | 0.861 | calbindin 2 | gi|34098931 | Q08331 | Calb2 | Signal transduction | E | 14 | 3 | 155 | 28105 | 4.92 |
| **2H** | SID4795 | 0.047 | 0.920 | calbindin 2 | gi|34098931 | Q8CCS7 | Calb2 | Signal transduction | E | 14 | 3 | 155 | 28105 | 4.92 |
| **2H** | SID663 | 0.014 | 1.068 | chaperonin subunit 5 (epsilon) | gi|6671702 | P80316 | Cct5 | Folding | E | 38 | 16 | 1116 | 59586 | 5.72 |
| **2H** | BID1868 | 0.005 | 1.268 | suppressor of K+ transport defect 3 | gi|6677983 | Q60649 | Clpb | Transcription | E | 15 | 8 | 474 | 75956 | 8.68 |
| **2H** | SID15251 | 0.035 | 0.911 | Clathrin light chain B (Lcb) | gi|62510439 | Q6IRU5 | Cltb | Sorting | E | 34 | 10 | 520 | 25156 | 4.56 |
| **2H** | SID15750 | 0.001 | 0.896 | Complexin-1 | gi|62471462 | P63040 | Cplx1 | Sorting | E | 38 | 4 | 323 | 15112 | 4.93 |
| **2H** | BID1723 | 0.049 | 0.969 | diazepam binding inhibitor isoform 2 | gi|6681137 | P31786 | Dbi | Lipid metabolism | E | 32 | 2 | 77 | 9994 | 8.78 |
| **2H** | SID1027 | 0.000 | 0.865 | NG,NG-dimethylarginine dimethylaminohydrolase 1 | gi|38371755 | Q9CWS0 | Ddah1 | Signal transduction | E | 41 | 12 | 602 | 31361 | 5.64 |
| **2H** | SID449 | 0.019 | 0.972 | postsynaptic density protein 95 | gi|6681195 | Q62108 | Dlg4 | Neuron | E | 2 | 1 | 52 | 26390 | 5.56 |
| **2H** | SID507 | 0.000 | 1.248 | Dihydropyrimidinase-related protein 2 | gi|1915913 | O08553 | Dpysl2 | Neuron | E | 7 | 3 | 183 | 62132 | 5.95 |
| **2H** | SID536 | 0.006 | 1.079 | Dihydropyrimidinase-related protein 2 | gi|1915913 | O08553 | Dpysl2 | Neuron | E | 9 | 3 | 219 | 62132 | 5.95 |
| **2H** | SID5670 | 0.026 | 1.056 | Dihydropyrimidinase-related protein 2 | gi|40254595 | O08553 | Dpysl2 | Neuron | M | 23 | 17 | 142 | 62239 | 5.95 |
| **2H** | SID569 | 0.014 | 0.905 | Dihydropyrimidinase-related protein 3 | gi|6681219 | Q62188 | Dpysl3 |  | E | 7 | 3 | 182 | 61897 | 6.04 |
| **2H** | SID15301 | 0.024 | 1.381 | EF-hand domain-containing protein 2 | gi|12847201 | Q9D8Y0 | Efhd2 | Translation | E | 4 | 1 | 52 | 25084 | 4.98 |
| **2H** | SID15303 | 0.012 | 0.734 | EF-hand domain-containing protein 2 | gi|12847201 | Q9D8Y0 | Efhd2 | Translation | E | 16 | 3 | 191 | 25084 | 4.98 |
| **2H** | SID15308 | 0.030 | 0.815 | EF-hand domain-containing protein 2 | gi|12847201 | Q9D8Y0 | Efhd2 | Translation | E | 39 | 8 | 500 | 25084 | 4.98 |
| **2H** | BID301 | 0.015 | 0.704 | eukaryotic translation initiation factor 2A | gi|26347343 | Q96EW9 | Eif2a | Translation | E | 5 | 2 | 152 | 64996 | 9.08 |
| **2H** | BID689 | 0.034 | 0.892 | eukaryotic translation initiation factor 3, subunit 3 (gamma) | gi|18079341 | Q91WK2 | Eif3s3 | Translation | E | 19 | 7 | 485 | 39807 | 6.2 |
| **2H** | BID538 | 0.003 | 1.059 | enolase 1, alpha non-neuron | gi|53734652 | Q5FW97 | Eno1 | CH Metabolism | E | 29 | 13 | 807 | 50209 | 8.01 |
| **2H** | SID829 | 0.024 | 1.203 | enolase 2, gamma neuronal | gi|7305027 | P17183 | Eno2 | CH Metabolism | E | 12 | 4 | 268 | 47267 | 4.99 |
| **2H** | SID406 | 0.012 | 0.735 | epsin 1 | gi|46195711 | Q80VP1 | Epn1 | Lipid metabolism | E | 7 | 3 | 194 | 60175 | 4.69 |
| **2H** | SID424 | 0.007 | 0.488 | epsin 1 | gi|46195711 | Q80VP1 | Epn1 | Lipid metabolism | E | 17 | 7 | 413 | 60175 | 4.69 |
| **2H** | SID998 | 0.003 | 0.879 | SPFH domain family, member 2 | gi|23956396 | Q8BFZ9 | Erlin2 |  | E | 13 | 5 | 366 | 37849 | 5.37 |
| **2H** | SID15823 | 0.033 | 0.921 | Fatty acid-binding protein, heart | gi|387397 | Q91W23 | Fabp3 | Lipid metabolism | E | 35 | 4 | 205 | 14810 | 6.11 |
| **2H** | SID1724 | 0.029 | 0.895 | Fatty acid-binding protein, epidermal | gi|6754450 | Q05816 | Fabp5 | Lipid metabolism | E | 32 | 4 | 236 | 15127 | 6.14 |
| **2H** | BID412 | 0.043 | 0.880 | Fascin (Singed-like protein) | gi|2498358 | Q61553 | Fscn1 | Cytoskeleton | E | 12 | 6 | 300 | 54371 | 6.21 |
| **2H** | BID1527 | 0.048 | 1.075 | Gamma-aminobutyric acid receptor-associated protein-like 2 | gi|6005768 | P60521 | Gabarapl2 | Sorting | E | 29 | 3 | 240 | 13658 | 7.82 |
| **2H** | SID15193 | 0.037 | 0.898 | Neuromodulin | gi|20271449 | P06837 | Gap43 | Neuron | E | 50 | 8 | 582 | 23618 | 4.63 |
| **2H** | SID873 | 0.004 | 0.881 | Neuromodulin | gi|6679935 | P06837 | Gap43 | Neuron | E | 50 | 8 | 582 | 23618 | 4.63 |
| **2H** | SID788 | 0.004 | 0.884 | guanine deaminase | gi|6753960 | Q9R111 | Gda | Nucleotide metabolism | E | 29 | 11 | 720 | 50981 | 5.36 |
| **2H** | SID1255 | 0.020 | 0.779 | Glod4 protein | gi|12840311 | Q9CPV4 | Glod4 | Metabolism | E | 7 | 2 | 105 | 30765 | 4.8 |
| **2H** | SID1256 | 0.001 | 0.873 | Glod4 protein | gi|12840311 | Q9CPV4 | Glod4 | Metabolism | E | 45 | 12 | 776 | 33296 | 5.28 |
| **2H** | SID1005 | 0.014 | 0.897 | Glutaredoxin-3 | gi|6840949 | Q9CQM9 | Glrx3 | Folding | E | 29 | 10 | 599 | 37758 | 5.42 |
| **2H** | SID1062 | 0.002 | 0.909 | guanine nucleotide-binding protein, beta-1 subunit | gi|6680045 | P62874 | Gnb1 | Signal transduction | E | 25 | 8 | 532 | 37353 | 5.6 |
| **2H** | SID15240 | 0.043 | 0.828 | guanine nucleotide-binding protein, beta-1 subunit | gi|6680045 | P62874 | Gnb1 | Signal transduction | E | 9 | 3 | 175 | 37353 | 5.6 |
| **2H** | SID5633 | 0.029 | 0.848 | Gelsolin [Precursor] | gi|18606238 | P13020 | Gsn | Cytoskeleton | E | 20 | 12 | 774 | 80712 | 5.52 |
| **2H** | BID1632 | 0.012 | 1.535 | alpha-globin | gi|193761 | Q61649 | Hba-a1 |  | E | 44 | 2 | 87 | 6212 | 6.82 |
| **2H** | SID1216 | 0.029 | 0.911 | haloacid dehalogenase-like hydrolase domain containing 2 isoform 1 | gi|12833114 | Q3UGR5 | Hdhd2 | Metabolism | E | 18 | 3 | 322 | 26635 | 5.81 |
| **2H** | SID15669 | 0.007 | 0.807 | haloacid dehalogenase-like hydrolase domain containing 2 isoform 1 | gi|21313310 | Q3UGR5 | Hdhd2 | Metabolism | E | 38 | 5 | 397 | 28712 | 5.7 |
| **2H** | SID545 | 0.017 | 0.916 | heterogeneous nuclear ribonucleoprotein K | gi|13384620 | P61979 | Hnrpk | Transcription | E | 28 | 12 | 634 | 50944 | 5.39 |
| **2H** | SID806 | 0.017 | 0.498 | Homer protein homolog 1 (VASP/Ena-related gene up-regulated during seizure and LTP) (Vesl-1) | gi|38605093 | Q9Z2Y3 | Homer1 | Signal transduction | E | 26 | 8 | 538 | 41388 | 5.39 |
| **2H** | SID480 | 0.041 | 1.084 | 78 kDa glucose-regulated protein [Precursor], Bip | gi|2598562 | P20029 | Hspa5 | Folding | E | 30 | 16 | 1072 | 72378 | 5.07 |
| **2H** | SID527 | 0.022 | 1.181 | Heat shock protein 8 | gi|42542422 | Q6NZD0 | Hspa8 | Folding | E | 40 | 20 | 1361 | 70648 | 5.22 |
| **2H** | SID530 | 0.042 | 1.044 | heat shock protein 2 [Mus musculus] | gi|31560686 | P17156 | Hspa9 | Folding | E | 45 | 28 | 1661 | 69599 | 5.51 |
| **2H** | SID496 | 0.048 | 1.040 | Heat shock protein 9A | gi|74219319 | Q7TSZ0 | Hspa9a | Folding | E | 1578 | 24 | 42 | 73416 | 5.81 |
| **2H** | SID684 | 0.029 | 1.028 | heat shock protein 65 | gi|51455 | P63038 | Hspd1 | Folding | E | 42 | 21 | 1380 | 60903 | 5.91 |
| **2H** | BID208 | 0.000 | 1.662 | KH domain-containing, RNA-binding, signal transduction-associated protein 1 | gi|602251 | Q60749 | Khdrbs1 | Transcription | E | 2 | 1 | 43 | 48456 | 8.92 |
| **2H** | BID219 | 0.000 | 1.352 | KH domain-containing, RNA-binding, signal transduction-associated protein 1 | gi|602251 | Q60749 | Khdrbs1 | Transcription | E | 11 | 4 | 227 | 48455 | 8.92 |
| **2H** | BID549 | 0.019 | 0.836 | mitogen activated protein kinase kinase 1 | gi|6678794 | P31938 | Map2k1 | Signal transduction | E | 10 | 4 | 233 | 43446 | 6.24 |
| **2H** | SID1242 | 0.001 | 0.923 | Microtubule-associated protein RP/EB family member 1 | gi|7106301 | Q61166 | Mapre1 | Cytoskeleton | E | 14 | 2 | 135 | 29997 | 5.12 |
| **2H** | SID1186 | 0.003 | 0.939 | Malate dehydrogenase, cytoplasmic | gi|387129 | P14152 | Mdh1 | CH Metabolism | E | 15 | 5 | 279 | 36454 | 6.16 |
| **2H** | BID818 | 0.013 | 1.093 | malate dehydrogenase 2, NAD (mitochondrial) | gi|31982186 | P08249 | Mdh2 | CH Metabolism | E | 51 | 15 | 944 | 35589 | 8.93 |
| **2H** | BID872 | 0.023 | 1.120 | malate dehydrogenase 2, NAD (mitochondrial) | gi|31982186 | P08249 | Mdh2 | CH Metabolism | E | 51 | 12 | 947 | 35589 | 8.93 |
| **2H** | SID15853 | 0.018 | 0.932 | Myotrophin | gi|6679961 | P62774 | Mtpn |  | E | 27 | 2 | 127 | 12853 | 5.27 |
| **2H** | SID1033 | 0.002 | 0.839 | Gamma-soluble NSF attachment protein | gi|26355647 | Q8C1T5 | Napg | Sorting | E | 34 | 11 | 667 | 34710 | 5.31 |
| **2H** | SID473 | 0.041 | 0.929 | NADH dehydrogenase (ubiquinone) Fe-S protein 1 | gi|21704020 | Q91VD9 | Ndufs1 | Energy Metabolism | E | 14 | 8 | 443 | 79698 | 5.51 |
| **2H** | SID5655 | 0.013 | 0.925 | NADH dehydrogenase (ubiquinone) Fe-S protein 1 | gi|21704020 | Q91VD9 | Ndufs1 | Energy Metabolism | E | 18 | 10 | 672 | 79698 | 5.51 |
| **2H** | SID15714 | 0.036 | 0.561 | neuron derived neurotrophic factor | gi|13384818 | Q9CQ45 | Nenf | Signal transduction | E | 30 | 4 | 271 | 18893 | 5.13 |
| **2H** | SID876 | 0.004 | 0.796 | NSFL1 cofactor p47 (p97 cofactor) | gi|38198665 | Q9CZ44 | Nsfl1c | Lipid metabolism | E | 44 | 14 | 836 | 40928 | 5.09 |
| **2H** | SID715 | 0.030 | 0.957 | prolyl 4-hydroxylase, beta polypeptide | gi|42415475 | P09103 | P4hb | Folding | E | 42 | 10 | 516 | 57023 | 4.77 |
| **2H** | SID755 | 0.017 | 0.860 | protein kinase C and casein kinase substrate in neurons 1 | gi|6754974 | Q61644 | Pacsin1 | Cytoskeleton | E | 32 | 13 | 744 | 50544 | 5.15 |
| **2H** | SID749 | 0.035 | 0.913 | protein kinase C and casein kinase substrate in neurons 1 | gi|46559412 | Q61644 | Pacsin1 | Cytoskeleton | E | 43 | 19 | 1136 | 50544 | 5.15 |
| **2H** | SID1316 | 0.006 | 0.939 | Platelet-activating factor acetylhydrolase IB beta subunit (PAF acetylhydrolase 30 kDa subunit) (PA | gi|3024348 | Q61206 | Pafah1b2 | Lipid metabolism | E | 5 | 2 | 124 | 25476 | 5.78 |
| **2H** | BID1178 | 0.010 | 0.895 | Protein-L-isoaspartate(D-aspartate) O-methyltransferase | gi|417489 | P23506 | Pcmt1 | Protein modification | E | 47 | 11 | 734 | 24619 | 7.1 |
| **2H** | SID15784 | 0.036 | 0.792 | Astrocytic phosphoprotein PEA-15 | gi|21426847 | Q62048 | Pea15 | Signal transduction | E | 7 | 1 | 58 | 15045 | 4.94 |
| **2H** | SID15785 | 0.012 | 0.825 | Astrocytic phosphoprotein PEA-15 | gi|23398567 | Q62048 | Pea15 | Signal transduction | E | 7 | 1 | 58 | 15045 | 4.94 |
| **2H** | SID1445 | 0.046 | 0.918 | Phosphatidylethanolamine-binding protein 1 | gi|74222953 | Q5EBQ2 | Pebp1 | Lipid metabolism | E | 6 | 1 | 75 | 20847 | 5.19 |
| **2H** | BID214 | 0.000 | 1.601 | 6-phosphofructokinase, muscle type (Phosphofructokinase 1) (Phosphohexokinase) (Phosphofructo-1-kin | gi|13638207 | P47857 | Pfkm | CH Metabolism | E | 14 | 8 | 498 | 85215 | 8.24 |
| **2H** | SID15780 | 0.028 | 1.058 | profilin 2 | gi|9506971 | Q9JJV2 | Pfn2 | Cytoskeleton | E | 32 | 4 | 211 | 15022 | 6,55 |
| **2H** | BID1073 | 0.050 | 0.953 | Phosphoglycerate mutase 1 | gi|53236962 | Q9DBJ1 | Pgam1 | CH Metabolism | E | 48 | 12 | 761 | 28685 | 6.67 |
| **2H** | BID595 | 0.035 | 0.957 | Phosphoglycerate kinase 1 | gi|129903 | P09411 | Pgk1 | CH Metabolism | E | 34 | 10 | 681 | 44508 | 7.53 |
| **2H** | BID603 | 0.034 | 0.895 | Phosphoglycerate kinase 1 | gi|129903 | P09411 | Pgk1 | CH Metabolism | E | 33 | 11 | 672 | 44522 | 7.53 |
| **2H** | BID604 | 0.013 | 0.880 | Phosphoglycerate kinase 1 | gi|129903 | P09411 | Pgk1 | CH Metabolism | E | 37 | 14 | 888 | 44508 | 7.53 |
| **2H** | BID1444 | 0.026 | 1.152 | protein (peptidyl-prolyl cis/trans isomerase) NIMA-interacting 1 | gi|12963653 | Q9QUR7 | Pin1 | Cell growth and death | E | 36 | 4 | 273 | 18359 | 8.93 |
| **2H** | BID374 | 0.031 | 1.113 | pyruvate kinase M | gi|74212815 | P52480 | Pkm2 | CH Metabolism | E | 18 | 9 | 493 | 43138 | 5.88 |
| **2H** | SID739 | 0.035 | 0.925 | protein kinase, cAMP dependent regulatory, type I, alpha | gi|26350511 | Q8C3Z4 | Prkar1a | Endocrine system | E | 14 | 5 | 338 | 45498 | 5.68 |
| **2H** | BID378 | 0.033 | 0.946 | nuclear matrix protein SNEV | gi|19527358 | Q99KP6 | Prpf19 | Degradation | E | 8 | 4 | 229 | 55204 | 6.14 |
| **2H** | BID601 | 0.050 | 0.853 | guanine nucleotide exchange factor for ADP ribosylation factor 6 isoform 1 | gi|83921581 | Q8CEA6 | Psd3 | Signal transduction | E | 15 | 8 | 425 | 58430 | 8.92 |
| **2H** | SID15620 | 0.006 | 0.953 | Proteasome subunit beta type 6 [Precursor] | gi|984938 | Q60692 | Psmb6 | Degradation | E | 19 | 4 | 274 | 21668 | 4.99 |
| **2H** | SID734 | 0.031 | 0.902 | 26S proteasome non-ATPase regulatory subunit 4 | gi|6679505 | O35226 | Psmd4 | Degradation | E | 23 | 7 | 455 | 40678 | 4.67 |
| **2H** | BID406 | 0.016 | 1.187 | Polypyrimidine tract binding protein 2 | gi|16307418 | Q91Z31 | Ptbp2 | Translation | E | 8 | 6 | 264 | 57525 | 8.53 |
| **2H** | SID1513 | 0.028 | 1.248 | prostaglandin E synthase 3 (cytosolic) | gi|26370131 | Q9CRH1 | Ptges3 | Lipid metabolism | E | 40 | 13 | 65 | 16561 | 4.81 |
| **2H** | SID612 | 0.046 | 0.923 | RAD23b homolog | gi|6679607 | P54728 | Rad23b | Degradation | E | 24 | 10 | 524 | 43490 | 4.77 |
| **2H** | SID1351 | 0.017 | 0.914 | Ran-specific GTPase-activating protein | gi|739241 | P34022 | Ranbp1 | Signal transduction | E | 17 | 4 | 249 | 5.15 | 23568 |
| **2H** | SID2339 | 0.008 | 0.918 | retinoblastoma binding protein 9 | gi|86439977 | Q80YU9 | Rbbp9 |  | E | 56 | 26 | 107 | 20898 | 5.64 |
| **2H** | SID763 | 0.009 | 0.918 | reticulocalbin 2 | gi|74146742 | Q3TE95 | Rcn2 |  | E | 36 | 9 | 639 | 41014 | 4.46 |
| **2H** | SID905 | 0.032 | 0.919 | protein 40kD | gi|226005 | P14206 | Rpsa | Translation | E | 20 | 5 | 298 | 32732 | 4.8 |
| **2H** | SID774 | 0.012 | 0.886 | Secernin-1 | gi|26328385 | Q9CZC8 | Scrn1 | Sorting | E | 19 | 8 | 448 | 46296 | 4.72 |
| **2H** | BID591 | 0.012 | 0.808 | SEC14-like protein 2 | gi|21362309 | Q99J08 | Sec14l2 | Lipid metabolism | E | 13 | 4 | 201 | 46271 | 6.69 |
| **2H** | BID631 | 0.012 | 0.924 | Septin-5 | gi|83305642 | Q9Z2Q6 | Sept5 | Cytoskeleton | E | 26 | 9 | 474 | 42721 | 6.21 |
| **2H** | BID1876 | 0.009 | 0.711 | Septin-7 | gi|9789726 | O55131 | Sept7 | Cytoskeleton | E | 10 | 4 | 251 | 50518 | 8.73 |
| **2H** | BID555 | 0.006 | 0.832 | Septin-7 | gi|9789726 | O55131 | Sept7 | Cytoskeleton | E | 2 | 1 | 57 | 50518 | 8.73 |
| **2H** | BID516 | 0.016 | 0.839 | Septin-7 | gi|28173550 | O55131 | Sept7 | Cytoskeleton | E | 25 | 11 | 639 | 50617 | 8.73 |
| **2H** | SID740 | 0.032 | 0.916 | Septin-8 | gi|29612695 | Q8CHH9 | Sept8 | Cytoskeleton | E | 11 | 5 | 308 | 52647 | 5.96 |
| **2H** | SID585 | 0.035 | 0.682 | Serine protease inhibitor A3K [Precursor] | gi|54173 | Q62257 | Serpina3k |  | E | 22 | 5 | 410 | 46643 | 5.04 |
| **2H** | SID1013 | 0.046 | 0.848 | Serine (Or cysteine) proteinase inhibitor, clade B (Ovalbumin), member 1a | gi|56206897 | Q5SUV7 | Serpinb1a |  | E | 5 | 2 | 116 | 42573 | 6.22 |
| **2H** | BID858 | 0.018 | 1.098 | sideroflexin 3 | gi|16716499 | Q91V61 | Sfxn3 |  | E | 32 | 9 | 568 | 35384 | 9.58 |
| **2H** | BID278 | 0.023 | 1.036 | Syntaxin-binding protein 1 | gi|6981602 | O08599 | Stxbp1 | Sorting | E | 13 | 8 | 453 | 67526 | 6.49 |
| **2H** | SID1203 | 0.027 | 0.725 | succinate-Coenzyme A ligase, ADP-forming, beta subunit | gi|46849708 | Q9Z2I9 | Sucla2 | CH Metabolism | E | 27 | 12 | 109 | 50082 | 6.57 |
| **2H** | BID225 | 0.012 | 0.852 | Synapsin-2 | gi|42406392 | Q64332 | Syn2 | Neuron | E | 22 | 10 | 690 | 63202 | 8.59 |
| **2H** | BID229 | 0.016 | 0.864 | Synapsin-2 | gi|42406392 | Q64332 | Syn2 | Neuron | E | 13 | 6 | 347 | 63202 | 8.59 |
| **2H** | BID231 | 0.020 | 0.838 | Synapsin-2 | gi|42406392 | Q64332 | Syn2 | Neuron | E | 10 | 5 | 341 | 63202 | 8.59 |
| **2H** | BID232 | 0.014 | 0.810 | Synapsin-2 | gi|42406392 | Q64332 | Syn2 | Neuron | E | 10 | 5 | 350 | 63202 | 8.59 |
| **2H** | BID200 | 0.002 | 1.479 | synapsin Ib | gi|3599473 | O88935 | SynI | Neuron | E | 11 | 6 | 402 | 69992 | 9.84 |
| **2H** | BID201 | 0.012 | 1.315 | synapsin Ib | gi|3599473 | O88935 | SynI | Neuron | E | 9 | 4 | 273 | 69992 | 9.84 |
| **2H** | BID202 | 0.000 | 0.719 | synapsin Ib | gi|3599473 | O88935 | SynI | Neuron | E | 13 | 7 | 407 | 69991 | 9.84 |
| **2H** | BID211 | 0.012 | 1.454 | synapsin Ib | gi|3599473 | O88935 | SynI | Neuron | E | 9 | 4 | 269 | 69991 | 9.84 |
| **2H** | BID215 | 0.015 | 0.809 | synapsin Ib | gi|3599473 | O88935 | SynI | Neuron | E | 25 | 12 | 781 | 69991 | 9.84 |
| **2H** | BID222 | 0.021 | 0.881 | synapsin Ib | gi|3599473 | O88935 | SynI | Neuron | E | 25 | 12 | 701 | 69992 | 9.84 |
| **2H** | BID227 | 0.015 | 1.165 | synapsin Ib | gi|3599473 | O88935 | SynI | Neuron | E | 24 | 13 | 774 | 69992 | 9.84 |
| **2H** | BID1280 | 0.019 | 1.243 | carboxyl-terminal modulator protein | gi|110626050 | Q3UUI3 | Them4 |  | M | ka | 3 | 221 | 26015 | 9.67 |
| **2H** | SID1266 | 0.017 | 0.921 | thiamine triphosphatase | gi|23346499 | Q8JZL3 | Thtpa | Cofactor Metabolism | E | 22 | 4 | 258 | 24249 | 4.67 |
| **2H** | SID15312 | 0.010 | 0.873 | toll interacting protein | gi|13591860 | Q9QZ06 | Tollip | Immune system | E | 18 | 5 | 302 | 30325 | 5.04 |
| **2H** | BID1160 | 0.018 | 1.032 | triosephosphate isomerase | gi|1864018 | P17751 | Tpi1 | CH Metabolism | E | 54 | 10 | 639 | 22492 | 5.62 |
| **2H** | BID1185 | 0.010 | 0.936 | triosephosphate isomerase | gi|1864018 | Q64513 | Tpi1 | CH Metabolism | E | 26 | 4 | 271 | 22492 | 5.62 |
| **2H** | SID15319 | 0.015 | 0.957 | Tropomyosin 1 alpha chain (Alpha-tropomyosin) | gi|20522240 | Q564G1 | Tpm1 | Cytoskeleton | E | 25 | 8 | 383 | 32689 | 4.71 |
| **2H** | SID15313 | 0.004 | 0.923 | Tropomyosin alpha-3 chain | gi|54912 | P21107 | Tpm3 | Cytoskeleton | E | 37 | 11 | 668 | 29003 | 4.75 |
| **2H** | SID15315 | 0.024 | 0.900 | Tropomyosin alpha-3 chain | gi|54912 | P21107 | Tpm3 | Cytoskeleton | E | 37 | 11 | 668 | 29003 | 4.75 |
| **2H** | SID15316 | 0.025 | 0.966 | Tropomyosin alpha-3 chain | gi|54912 | P21107 | Tpm3 | Cytoskeleton | E | 37 | 11 | 668 | 29003 | 4.75 |
| **2H** | SID15318 | 0.030 | 0.879 | Tropomyosin alpha-3 chain | gi|54912 | P21107 | Tpm3 | Cytoskeleton | E | 27 | 8 | 456 | 29003 | 4.57 |
| **2H** | BID589 | 0.003 | 0.770 | Tu translation elongation factor, mitochondrial | gi|27370092 | Q8BFR5 | Tufm | Translation | E | 13 | 6 | 419 | 49477 | 7.23 |
| **2H** | SID15753 | 0.018 | 0.937 | Ubiquitin-conjugating enzyme E2 N | gi|12838544 | P61089 | Ube2n | Degradation | E | 22 | 3 | 150 | 6.13 | 17127 |
| **2H** | SID15276 | 0.038 | 0.894 | ubiquitin-conjugating enzyme E2R 2 | gi|13385778 | Q6ZWZ2 | Ube2r2 | Degradation | E | 22 | 12 | 56 | 27149 | 4.26 |
| **2H** | SID515 | 0.013 | 0.918 | Ubiquilin-1 | gi|16307349 | Q8R317 | Ubqln1 | Degradation | E | 10 | 3 | 199 | 4.86 | 61937 |
| **2H** | SID516 | 0.026 | 0.899 | Ubiquilin-1 | gi|16307349 | Q8R317 | Ubqln1 | Degradation | E | 10 | 3 | 199 | 4.86 | 61937 |
| **2H** | SID446 | 0.025 | 0.900 | Ubiquilin-2 | gi|6014493 | Q9QZM0 | Ubqln2 | Degradation | E | 12 | 5 | 280 | 67308 | 5.16 |
| **2H** | SID1377 | 0.001 | 0.940 | ubiquitin carboxy-terminal hydrolase L1 | gi|61098212 | Q00981 | Uchl1 | Degradation | E | 38 | 6 | 378 | 24822 | 5.14 |
| **2H** | SID1385 | 0.027 | 1.300 | ubiquitin carboxy-terminal hydrolase L1 | gi|61098212 | Q9R0P9 | Uchl1 | Degradation | E | 38 | 6 | 378 | 24822 | 5.14 |
| **2H** | SID851 | 0.033 | 1.444 | ubiquitin carboxyl-terminal esterase L3 | gi|7710106 | Q9JKB1 | Uchl3 | Degradation | E | 22 | 4 | 300 | 26162 | 5.08 |
| **2H** | BID1039 | 0.016 | 0.741 | Voltage-dependent anion channel 3 | gi|59807690 | Q5EBQ0 | Vdac3 | Signal transduction | E | 31 | 7 | 472 | 30864 | 8.96 |
| **2H** | SID1310 | 0.031 | 1.197 | WW domain binding protein 2 | gi|8394539 | P97765 | Wbp2 |  | E | 5 | 1 | 70 | 28013 | 5.94 |
| **2H** | SID1305 | 0.024 | 0.954 | 14-3-3 protein gamma | gi|9507245 | P61982 | Ywhag | Cell growth and death | E | 34 | 9 | 501 | 28285 | 4.8 |
| **7C** | BID1829 | 0.045 | 0.670 | 4-aminobutyrate aminotransferase | gi|26331164 | P61922 | Abat | AS Metabolism | E | 10 | 4 | 209 | 50205 | 8.79 |
| **7C** | BID709 | 0.045 | 1.347 | Acetyl-CoA acetyltransferase, cytosolic | gi|13435978 | Q8CAY6 | Acat2 | AS Metabolism | E | 7 | 2 | 117 | 38123 | 8.62 |
| **7C** | BID539 | 0.002 | 1.144 | Acyl-coenzyme A thioesterase 9, mitochondrial [Precursor] | gi|5103680 | Q9R0X4 | Acot9 | Xenobiotics | E | 13 | 6 | 295 | 50458 | 8.73 |
| **7C** | SID927 | 0.048 | 0.953 | Actin, cytoplasmic 1 | gi|49868 | P60710 | Actb | Cytoskeleton | E | 14 | 4 | 251 | 39161 | 8.16 |
| **7C** | SID15761 | 0.049 | 0.711 | Actin, cytoplasmic 1 | gi|74190672 | P60710 | Actb | Cytoskeleton | E | 5 | 2 | 101 | 41738 | 5.78 |
| **7C** | SID15200 | 0.001 | 1.100 | gamma-actin | gi|809561 | P63260 | Actg1 | Cytoskeleton | E | 10 | 3 | 150 | 40992 | 5.02 |
| **7C** | BID780 | 0.026 | 0.882 | annexin A2 | gi|6996913 | P07356 | Anxa2 | Cytoskeleton | E | 16 | 5 | 273 | 38585 | 4.95 |
| **7C** | SID2679 | 0.026 | 1.093 | Apolipoprotein E | gi|114041 | P08226 | Apoe | Lipid metabolism | E | 31 | 10 | 620 | 35844 | 5.29 |
| **7C** | SID985 | 0.029 | 0.947 | ADP-ribosylation factor interacting protein 3 | gi|31981363 | Q8K221 | Arfip2 | Signal transduction | E | 12 | 3 | 233 | 37776 | 7.71 |
| **7C** | SID775 | 0.013 | 0.915 | ATP synthase subunit beta, mitochondrial [Precursor] | gi|23272966 | P56480 | Atp5b | Energy Metabolism | E | 19 | 7 | 624 | 56632 | 5.90 |
| **7C** | SID772 | 0.040 | 1.053 | ATP synthase subunit beta, mitochondrial [Precursor] | gi|23272966 | P56480 | Atp5b | Energy Metabolism | E | 37 | 15 | 960 | 56632 | 5.7 |
| **7C** | SID15757 | 0.001 | 1.151 | ATP synthase delta chain, mitochondrial [Precursor] | gi|12847456 | Q9D3D9 | Atp5d | Energy Metabolism | E | 17 | 4 | 236 | 17619 | 8.42 |
| **7C** | SID15618 | 0.044 | 1.052 | ATP synthase D chain, mitochondrial | gi|21313679 | Q9DCX2 | Atp5h | Energy Metabolism | E | 17 | 3 | 157 | 18738 | 7.62 |
| **7C** | SID1468 | 0.002 | 1.237 | ATP synthase D chain, mitochondrial | gi|21313679 | Q9DCX2 | Atp5h | Energy Metabolism | E | 82 | 12 | 642 | 18738 | 5.33 |
| **7C** | SID15879 | 0.007 | 1.079 | ATP synthase coupling factor 6, mitochondrial [Precursor] | gi|7949005 | P97450 | Atp5j | Energy Metabolism | E | 46 | 4 | 220 | 12489 | 5.36 |
| **7C** | SID5669 | 0.003 | 1.087 | ATPase, H+ transporting, V1 subunit A, isoform 1 | gi|1718086 | P50516 | Atp6v1a | Energy Metabolism | E | 21 | 10 | 651 | 68224 | 8.26 |
| **7C** | BID1084 | 0.005 | 0.973 | Methylglutaconyl-CoA hydratase, mitochondrial [Precursor] | gi|20072952 | Q9JLZ3 | Auh | AS Metabolism | E | 34 | 10 | 539 | 32621 | 5.39 |
| **7C** | SID389 | 0.036 | 1.069 | Myc box-dependent-interacting protein 1 | gi|6753050 | O08539 | Bin1 | Cell growth and death | E | 20 | 8 | 471 | 64430 | 5.31 |
| **7C** | SID403 | 0.040 | 1.072 | Myc box-dependent-interacting protein 1 | gi|6753050 | O08539 | Bin1 | Cell growth and death | E | 12 | 5 | 364 | 64430 | 7.63 |
| **7C** | SID958 | 0.047 | 1.170 | bisphosphate 3'-nucleotidase 1 | gi|39652626 | Q3U449 | Bpnt1 | Energy Metabolism | M | 28 | 7 | 76 | 33000 | 8.96 |
| **7C** | SID4797 | 0.006 | 1.211 | calbindin 2 | gi|34098931 | Q8CCS7 | Calb2 | Signal transduction | E | 14 | 3 | 155 | 28105 | 5.62 |
| **7C** | SID15715 | 0.012 | 1.129 | Complexin-2 | gi|5729783 | P84086 | Cplx2 | Sorting | E | 11 | 1 | 59 | 15385 | 6.14 |
| **7C** | BID622 | 0.039 | 1.084 | citrate synthase | gi|13385942 | Q9CZU6 | Cs | CH Metabolism | E | 6 | 3 | 160 | 51703 | 9.25 |
| **7C** | BID1205 | 0.008 | 1.050 | es1 protein | gi|20070420 | Q9D172 | D10Jhu81e |  | E | 15 | 4 | 205 | 28073 | 7.64 |
| **7C** | BID1723 | 0.023 | 0.932 | diazepam binding inhibitor isoform 2 | gi|6681137 | P31786 | Dbi | Lipid metabolism | E | 32 | 2 | 111 | 9994 | 5.6 |
| **7C** | BID302 | 0.039 | 0.726 | eukaryotic translation initiation factor 2A | gi|26347343 | Q8BJW6 | Eif2a | Translation | E | 2 | 1 | 57 | 64996 | 5.37 |
| **7C** | SID832 | 0.003 | 1.091 | enolase 2, gamma neuronal | gi|7305027 | P17183 | Eno2 | CH Metabolism | E | 39 | 13 | 874 | 47267 | 7.71 |
| **7C** | SID1324 | 0.002 | 1.095 | Endoplasmic reticulum protein ERp29 | gi|19526463 | P57759 | Erp29 | Sorting | E | 25 | 6 | 357 | 28805 | 4.99 |
| **7C** | BID768 | 0.043 | 0.943 | similar to glyceraldehyde-3-phosphate dehydrogenase | gi|6679937 | P16858 | Gapdh | CH Metabolism | E | 14 | 4 | 249 | 35787 | 9.05 |
| **7C** | BID1181 | 0.020 | 1.099 | Protein NipSnap2 (Glioblastoma amplified sequence) | gi|2769258 | O55126 | Gbas |  | E | 23 | 6 | 382 | 32912 | 6.07 |
| **7C** | SID1005 | 0.015 | 0.918 | Glutaredoxin-3 | gi|6840949 | Q9CQM9 | Glrx3 | Folding | E | 29 | 10 | 599 | 37758 | 9.31 |
| **7C** | BID330 | 0.007 | 0.864 | MKIAA0838 protein | gi|28478945 | Q69ZX9 | Gls | AS Metabolism | E | 8 | 3 | 160 | 73916 | 9.36 |
| **7C** | SID1058 | 0.006 | 0.898 | guanine nucleotide-binding protein, beta-1 subunit | gi|6680045 | P62874 | Gnb1 | Signal transduction | E | 25 | 8 | 532 | 37353 | 5.58 |
| **7C** | SID15240 | 0.000 | 0.904 | guanine nucleotide-binding protein, beta-1 subunit | gi|6680045 | P62874 | Gnb1 | Signal transduction | E | 9 | 3 | 175 | 37353 | 9 |
| **7C** | SID1062 | 0.034 | 0.930 | Guanine nucleotide-binding protein G(I)/G(S)/G(T) subunit beta 2 | gi|984551 | P62880 | Gnb2 | Signal transduction | E | 7 | 3 | 146 | 37309 | 5.6 |
| **7C** | SID1104 | 0.047 | 0.943 | Guanine nucleotide-binding protein G(I)/G(S)/G(T) subunit beta 2 | gi|984551 | P62880 | Gnb2 | Signal transduction | E | 36 | 9 | 597 | 37309 | 5.60 |
| **7C** | BID715 | 0.025 | 1.047 | mitochondrial aspartate aminotransferase | gi|192050 | P05202 | Got2 | AS Metabolism | E | 19 | 8 | 520 | 47723 | 5.35 |
| **7C** | BID723 | 0.007 | 1.054 | mitochondrial aspartate aminotransferase | gi|192050 | P05202 | Got2 | AS Metabolism | E | 28 | 12 | 732 | 47723 | 5.12 |
| **7C** | BID1139 | 0.022 | 0.775 | glutathione S-transferase, mu 1 | gi|6754084 | P10649 | Gstm1 | AS Metabolism | E | 30 | 7 | 408 | 25953 | 5.56 |
| **7C** | BID551 | 0.037 | 0.947 | mitochondrial trifunctional protein, beta subunit | gi|21704100 | Q99JY0 | Hadhb | Lipid metabolism | E | 33 | 16 | 813 | 51353 | 9.05 |
| **7C** | BID752 | 0.026 | 0.836 | 3-hydroxyisobutyryl-Coenzyme A hydrolase | gi|22122625 | Q8QZS1 | Hibch | AS Metabolism | E | 16 | 6 | 351 | 43010 | 5.52 |
| **7C** | SID15209 | 0.025 | 1.130 | heterogeneous nuclear ribonucleoprotein C | gi|8393544 | Q9Z204 | Hnrnpc | Translation | E | 18 | 6 | 358 | 34364 | 6.1 |
| **7C** | BID746 | 0.016 | 0.759 | PREDICTED: similar to heterogeneous nuclear ribonucleoprotein H3 isoform a isoform 6 | gi|94388237 |  | Hnrph3 | Translation | E | 9 | 2 | 104 | 35159 | 5.56 |
| **7C** | SID806 | 0.014 | 0.549 | Homer protein homolog 1 (VASP/Ena-related gene up-regulated during seizure and LTP) (Vesl-1) | gi|38605093 | Q9Z2Y3 | Homer1 | Signal transduction | E | 26 | 8 | 538 | 41388 | 7.63 |
| **7C** | SID480 | 0.025 | 1.087 | 78 kDa glucose-regulated protein [Precursor] | gi|2506545 | P20029 | Hspa5 | Folding | E | 30 | 16 | 1072 | 72378 | 4.56 |
| **7C** | SID680 | 0.050 | 1.072 | heat shock protein 65 | gi|51455 | P63038 | Hspd1 | Folding | E | 41 | 19 | 1434 | 60903 | 5.31 |
| **7C** | SID693 | 0.002 | 1.094 | heat shock protein 65 | gi|51452 | P63038 | Hspd1 | Folding | E | 24 | 12 | 755 | 60903 | 8.29 |
| **7C** | SID655 | 0.015 | 0.905 | Alpha-Internexin | gi|17390900 | P46660 | Ina | Cytoskeleton | E | 50 | 27 | 1724 | 55349 | 8.78 |
| **7C** | BID1869 | 0.033 | 0.788 | inositol 1,4,5-trisphosphate 3-kinase A | gi|22122643 | Q8R071 | Itpka | Signal transduction | E | 26 | 11 | 647 | 50903 | 5.72 |
| **7C** | BID853 | 0.018 | 1.057 | L-lactate dehydrogenase | gi|74217959 | Q3TCI7 | Ldha | CH Metabolism | E | 34 | 10 | 641 | 36476 | 5.27 |
| **7C** | SID1088 | 0.047 | 0.958 | lactate dehydrogenase 2, B chain | gi|6678674 | P16125 | Ldhb | CH Metabolism | E | 40 | 14 | 929 | 36549 | 4.92 |
| **7C** | SID15239 | 0.002 | 0.877 | leucine zipper transcription factor-like 1 | gi|15277319 | Q3UHL8 | Lztfl1 |  | E | 19 | 2 | 80 | 34752 | 9.57 |
| **7C** | BID979 | 0.021 | 0.629 | mitochondrial ribosomal protein L45 | gi|12847837 | Q9CSZ8 | Mrpl45 | Translation | E | 6 | 2 | 107 | 32812 | 8.85 |
| **7C** | SID15853 | 0.035 | 0.928 | Myotrophin | gi|6679961 | P62774 | Mtpn |  | E | 27 | 2 | 127 | 12853 | 8.23 |
| **7C** | SID15744 | 0.002 | 1.184 | Myosin light polypeptide 6 | gi|17986258 | Q60605 | Myl6 | Cytoskeleton | E | 31 | 4 | 277 | 16919 | 5.76 |
| **7C** | SID1033 | 0.035 | 0.960 | Gamma-soluble NSF attachment protein | gi|110625902 | Q8C1T5 | Napg | Sorting | E | 34 | 11 | 667 | 34710 | 6.36 |
| **7C** | SID1433 | 0.022 | 0.823 | 5'(3')-deoxyribonucleotidase, cytosolic type | gi|7657031 | Q9JM14 | Nt5c | Cofactor Metabolism | E | 15 | 2 | 119 | 23062 | 5.03 |
| **7C** | SID765 | 0.050 | 1.047 | Pyruvate dehydrogenase protein X component, mitochondrial precursor | gi|28201978 | Q8BKZ9 | Pdhx | CH Metabolism | M | 27 | 28 | 86 | 53965 | 5.91 |
| **7C** | SID15619 | 0.040 | 1.065 | Phosphatidylethanolamine-binding protein 1 | gi|74222953 | Q3TGC5 | Pebp1 | Lipid metabolism | E | 56 | 6 | 398 | 20876 | 4.56 |
| **7C** | BID1840 | 0.002 | 0.885 | Phosphoglycerate kinase 1 | gi|129903 | P09411 | Pgk1 | CH Metabolism | E | 43 | 16 | 909 | 44508 | 4.95 |
| **7C** | BID395 | 0.017 | 0.873 | pleiotropic regulator 1 | gi|2832298 | Q922V4 | Plrg1 | Translation | E | 5 | 2 | 89 | 56928 | 5.62 |
| **7C** | SID15297 | 0.021 | 0.837 | protein phosphatase 1, regulatory (inhibitor) subunit 2 | gi|12835461 | Q9CT86 | Ppp1r2 | Signal transduction | E | 6 | 1 | 74 | 18817 | 5.52 |
| **7C** | BID1274 | 0.030 | 0.900 | peroxiredoxin 1 | gi|6754976 | P35700 | Prdx1 | Degradation | E | 20 | 5 | 224 | 22162 | 4.92 |
| **7C** | SID15634 | 0.021 | 0.901 | Peroxiredoxin-2 | gi|3603241 | Q61171 | Prdx2 | Degradation | M | 37 | 7 | 115 | 21778 | 8.72 |
| **7C** | BID1476 | 0.030 | 0.952 | peroxiredoxin 5 | gi|10129957 | P99029 | Prdx5 | Degradation | E | 48 | 8 | 494 | 17004 | 9.43 |
| **7C** | SID1314 | 0.048 | 0.900 | Proteasome subunit alpha type 3 (Proteasome component C8) (Macropain subunit C8) (Multicatalytic en | gi|3914438 | O70435 | Psma3 | Degradation | E | 25 | 7 | 350 | 28387 | 5.06 |
| **7C** | BID1213 | 0.046 | 1.088 | Proteasome subunit beta type 1 [Precursor] | gi|1165123 | O09061 | Psmb1 | Degradation | E | 36 | 7 | 407 | 24664 | 5.2 |
| **7C** | SID15620 | 0.005 | 1.158 | Proteasome subunit beta type 6 [Precursor] | gi|984938 | Q60692 | Psmb6 | Degradation | E | 19 | 4 | 274 | 21668 | 5.44 |
| **7C** | SID964 | 0.021 | 1.136 | Transcriptional activator protein Pur-alpha | gi|34878862 | P42669 | Pura | Cell growth and death | E | 11 | 4 | 205 | 34862 | 8.73 |
| **7C** | SID15796 | 0.015 | 0.905 | Parvalbumin alpha | gi|53819 | P32848 | Pvalb | Signal transduction | E | 52 | 5 | 279 | 11937 | 9.08 |
| **7C** | BID1828 | 0.009 | 0.908 | Septin-11 | gi|26324430 | Q8C1B7 | Sept11 | Cytoskeleton | E | 17 | 6 | 388 | 49649 | 4.99 |
| **7C** | BID625 | 0.028 | 1.054 | Septin-5 | gi|3986409 | Q9Z2Q6 | Sept5 | Cytoskeleton | E | 10 | 3 | 154 | 40215 | 9.53 |
| **7C** | BID546 | 0.042 | 1.082 | Septin-7 | gi|9789726 | O55131 | Sept7 | Cytoskeleton | E | 19 | 8 | 438 | 50518 | 7.53 |
| **7C** | BID1876 | 0.032 | 1.205 | Septin-7 | gi|9789726 | O55131 | Sept7 | Cytoskeleton | E | 10 | 4 | 251 | 50518 | 5.76 |
| **7C** | BID408 | 0.015 | 1.231 | Plasminogen activator inhibitor 1 RNA-binding protein | gi|12846595 | Q9CY58 | Serbp1 | Translation | E | 15 | 4 | 230 | 42918 | 4.76 |
| **7C** | SID736 | 0.037 | 1.042 | serine (or cysteine) proteinase inhibitor, clade A, member 1e | gi|6678087 | Q00898 | Serpina1e | Degradation | B | 24 | 9 | 75 | 45862 | 5.91 |
| **7C** | BID194 | 0.003 | 0.822 | Zfp162 protein | gi|33286894 | Q64213 | Sf1 | Transcription | M | 34 | 14 | 83 | 59651 | 5.24 |
| **7C** | BID414 | 0.001 | 1.169 | Signal recognition particle 54 kDa protein | gi|54194 | P14576 | Srp54 | Sorting | E | 22 | 9 | 643 | 55709 | 9.53 |
| **7C** | SID15699 | 0.000 | 0.762 | Stathmin | gi|9789995 | P54227 | Stmn1 | Signal transduction | E | 29 | 5 | 234 | 17264 | 7.55 |
| **7C** | SID15700 | 0.029 | 1.040 | Stathmin | gi|9789995 | P54227 | Stmn1 | Signal transduction | E | 26 | 5 | 233 | 17264 | 5.07 |
| **7C** | BID1186 | 0.006 | 0.916 | triosephosphate isomerase | gi|1864018 | Q64513 | Tpi1 | CH Metabolism | E | 40 | 7 | 419 | 22492 | 8.44 |
| **7C** | SID1395 | 0.001 | 0.930 | Translationally-controlled tumor protein | gi|6678437 | P63028 | Tpt1 | Signal transduction | E | 33 | 6 | 307 | 19450 | 5.60 |
| **7C** | SID15639 | 0.042 | 0.816 | ubiquitin carboxy-terminal hydrolase L1 | gi|6755929 | Q00981 | Uchl1 | Degradation | E | 17 | 5 | 245 | 24822 | 5.24 |
| **7C** | BID894 | 0.040 | 0.920 | Voltage-dependent anion-selective channel protein 1 | gi|6755963 | Q60932 | Vdac1 | Signal transduction | E | 33 | 8 | 521 | 30737 | 5.42 |
| **7H** | BID831 | 0.039 | 1.240 | 2310005E10Rik protein | gi|20073097 | Q8R318 | 2310005E10Rik |  | E | 32 | 3 | 166 | 13402 | 5.65 |
| **7H** | SID1278 | 0.024 | 0.812 | Actin, cytoplasmic 1 | gi|49868 | P60710 | Actb | Cytoskeleton | E | 13 | 4 | 232 | 39161 | 5.78 |
| **7H** | SID920 | 0.039 | 0.877 | gamma-actin | gi|809561 | P63260 | Actg1 | Cytoskeleton | E | 32 | 10 | 522 | 40992 | 5.56 |
| **7H** | SID15200 | 0.004 | 1.120 | gamma-actin | gi|809561 | P63260 | Actg1 | Cytoskeleton | E | 10 | 3 | 150 | 40992 | 5.56 |
| **7H** | BID1704 | 0.049 | 1.118 | Acylphosphatase-2 (Acylphosphate phosphohydrolase 2) (Acylphosphatase, muscle type isozyme) | gi|3023245 | P56375 | Acyp2 | Xenobiotics | E | 23 | 3 | 173 | 10888 | 8.71 |
| **7H** | SID15623 | 0.002 | 0.942 | adenylate kinase 1 | gi|10946936 | Q9R0Y5 | Ak1 | Nucleotide metabolism | E | 38 | 10 | 619 | 23102 | 5.7 |
| **7H** | BID654 | 0.023 | 1.061 | aldolase 1, A isoform | gi|6671539 | Q5FWB7 | Aldoa | CH Metabolism | E | 48 | 15 | 1017 | 39331 | 8.31 |
| **7H** | BID657 | 0.010 | 1.034 | aldolase 1, A isoform | gi|6671539 | Q5FWB7 | Aldoa | CH Metabolism | E | 49 | 15 | 1021 | 39331 | 8.31 |
| **7H** | BID677 | 0.046 | 0.943 | aldolase 3, C isoform | gi|60687506 | P05063 | Aldoc | CH Metabolism | E | 39 | 11 | 684 | 39370 | 6.67 |
| **7H** | SID2679 | 0.043 | 1.091 | Apolipoprotein E | gi|74178217 | P08226 | Apoe | Lipid metabolism | E | 26 | 9 | 515 | 35884 | 5.56 |
| **7H** | BID1338 | 0.012 | 0.848 | actin related protein 2/3 complex, subunit 3 | gi|9790141 | Q9JM76 | Arpc3 | Cytoskeleton | E | 19 | 3 | 125 | 20511 | 8.78 |
| **7H** | SID2062 | 0.026 | 0.774 | ATP synthase subunit beta, mitochondrial [Precursor] | gi|23272966 | Q8CI65 | Atp5b | Energy Metabolism | E | 17 | 6 | 337 | 56632 | 5.24 |
| **7H** | SID772 | 0.021 | 1.059 | ATP synthase subunit beta, mitochondrial [Precursor] | gi|23272966 | P56480 | Atp5b | Energy Metabolism | E | 37 | 15 | 960 | 56632 | 5.24 |
| **7H** | SID15757 | 0.047 | 1.113 | ATP synthase delta chain, mitochondrial [Precursor] | gi|12847456 | Q9D3D9 | Atp5d | Energy Metabolism | E | 17 | 4 | 236 | 17619 | 5.03 |
| **7H** | SID1468 | 0.019 | 1.244 | ATP synthase D chain, mitochondrial | gi|21313679 | Q9DCX2 | Atp5h | Energy Metabolism | E | 82 | 12 | 642 | 18738 | 5.52 |
| **7H** | SID15883 | 0.008 | 1.080 | ATP synthase coupling factor 6, mitochondrial [Precursor] | gi|7949005 | P97450 | Atp5j | Energy Metabolism | E | 39 | 3 | 163 | 12489 | 9.36 |
| **7H** | SID451 | 0.017 | 1.078 | Myc box-dependent-interacting protein 1 | gi|6753050 | O08539 | Bin1 | Cell growth and death | E | 8 | 3 | 181 | 64430 | 4.95 |
| **7H** | SID457 | 0.039 | 1.072 | Myc box-dependent-interacting protein 1 | gi|6753050 | O08539 | Bin1 | Cell growth and death | E | 10 | 4 | 233 | 64430 | 4.95 |
| **7H** | BID1378 | 0.032 | 0.949 | cofilin 2, muscle [Mus musculus] | gi|6671746 | P45591 | Cfl2 | Cytoskeleton | M | ka | 2 | 107 | 18698 | 7.66 |
| **7H** | BID911 | 0.043 | 0.657 | Coiled-coil-helix-coiled-coil-helix domain-containing protein 6 | gi|27754146 | Q91VN4 | Chchd6 |  | E | 45 | 11 | 601 | 29780 | 8.41 |
| **7H** | SID906 | 0.049 | 0.917 | creatine kinase, brain | gi|10946574 | Q04447 | Ckb | AS Metabolism | E | 9 | 3 | 164 | 42686 | 5.4 |
| **7H** | BID597 | 0.030 | 1.086 | Creatine kinase, ubiquitous mitochondrial precursor | gi|6753428 | P30275 | Ckmt1 | AS Metabolism | E | 33 | 15 | 833 | 46974 | 8.39 |
| **7H** | SID1348 | 0.037 | 1.076 | Putative ATP-dependent Clp protease proteolytic subunit, mitochondrial [Precursor] | gi|12805083 | O88696 | Clpp | Degradation | E | 15 | 8 | 474 | 75956 | 8.68 |
| **7H** | SID15831 | 0.021 | 1.064 | Cytochrome c oxidase subunit 5A, mitochondrial [Precursor] | gi|117099 | P12787 | Cox5a | Energy Metabolism | E | 11 | 3 | 161 | 16020 | 6.08 |
| **7H** | SID581 | 0.000 | 1.193 | copine VI | gi|6753510 | Q80W08 | Cpne6 |  | E | 13 | 7 | 372 | 61742 | 5.37 |
| **7H** | BID1138 | 0.034 | 0.910 | es1 protein | gi|20070420 | Q9D172 | D10Jhu81e |  | E | 13 | 4 | 178 | 28073 | 9 |
| **7H** | BID1091 | 0.024 | 0.797 | 3,2-trans-enoyl-CoA isomerase, mitochondrial precursor (Dodecenoyl-CoA isomerase) (Delta(3),Delta(2 | gi|1169205 | P42125 | Dci | Lipid metabolism | E | 21 | 5 | 282 | 32058 | 8.87 |
| **7H** | SID1027 | 0.028 | 0.832 | NG,NG-dimethylarginine dimethylaminohydrolase 1 | gi|38371755 | Q9CWS0 | Ddah1 | Signal transduction | E | 41 | 12 | 602 | 31361 | 5.64 |
| **7H** | SID5680 | 0.004 | 1.078 | Dihydrolipoamide S-acetyltransferase | gi|20071885 | Q8R339 | Dlat | CH Metabolism | E | 17 | 7 | 476 | 59047 | 5.71 |
| **7H** | SID597 | 0.039 | 1.541 | Dihydropyrimidinase-related protein 2 | gi|40254595 | O08553 | Dpysl2 | Neuron | E | 22 | 9 | 558 | 62239 | 5.95 |
| **7H** | SID569 | 0.037 | 1.057 | Dihydropyrimidinase-related protein 3 | gi|6681219 | Q62188 | Dpysl3 |  | E | 7 | 3 | 182 | 61897 | 6.04 |
| **7H** | BID267 | 0.028 | 0.942 | dihydropyrimidinase-related protein 4 | gi|34328211 | O35098 | Dpysl4 |  | E | 13 | 6 | 371 | 61922 | 6.51 |
| **7H** | SID426 | 0.024 | 1.079 | Cytoplasmic dynein 1 intermediate chain 1 | gi|3493589 | O88485 | Dync1i1 | Sorting | E | 3 | 1 | 93 | 68422 | 5.13 |
| **7H** | BID1740 | 0.044 | 1.118 | dynein light chain 2 | gi|18087731 | Q9D0M5 | Dynll2 | Cytoskeleton | E | 24 | 1 | 60 | 10343 | 6.81 |
| **7H** | SID15865 | 0.024 | 1.061 | Dynein light chain roadblock-type 1 | gi|21735425 | P62627 | Dynlrb1 | Cytoskeleton | E | 21 | 1 | 52 | 10969 | 6.58 |
| **7H** | BID525 | 0.027 | 1.041 | enolase 1, alpha non-neuron | gi|53734652 | Q5XJG8 | Eno1 | CH Metabolism | E | 19 | 7 | 421 | 50209 | 8.01 |
| **7H** | SID15253 | 0.015 | 0.825 | enolase 2, gamma neuronal | gi|7305027 | P17183 | Eno2 | CH Metabolism | E | 17 | 4 | 301 | 47267 | 4.99 |
| **7H** | SID824 | 0.029 | 1.049 | enolase 2, gamma neuronal | gi|7305027 | P17183 | Eno2 | CH Metabolism | E | 49 | 14 | 995 | 47267 | 4.99 |
| **7H** | SID836 | 0.013 | 0.866 | enolase 2, gamma neuronal | gi|7305027 | P17183 | Eno2 | CH Metabolism | E | 20 | 8 | 439 | 47267 | 4.99 |
| **7H** | BID1469 | 0.006 | 1.067 | Alpha-endosulfine | gi|9624979 | P60840 | Ensa | Endocrine system | E | 33 | 3 | 164 | 13327 | 6.62 |
| **7H** | SID1324 | 0.037 | 1.091 | Endoplasmic reticulum protein ERp29 | gi|19526463 | P57759 | Erp29 | Sorting | E | 25 | 6 | 357 | 28805 | 5.9 |
| **7H** | SID1724 | 0.037 | 0.915 | Fatty acid-binding protein, epidermal | gi|6754450 | Q05816 | Fabp5 | Lipid metabolism | E | 32 | 4 | 236 | 15127 | 6.14 |
| **7H** | BID836 | 0.036 | 0.871 | Fumarylacetoacetate hydrolase domain containing 2A | gi|29366814 | Q8K0V8 | Fahd2a | Metabolism | E | 13 | 3 | 210 | 34654 | 8.42 |
| **7H** | SID720 | 0.046 | 0.972 | FK506-binding protein 4 | gi|6753882 | P30416 | Fkbp4 | Folding | E | 26 | 10 | 557 | 51540 | 5.54 |
| **7H** | BID793 | 0.030 | 0.864 | similar to glyceraldehyde-3-phosphate dehydrogenase | gi|6679937 | P16858 | Gapdh | CH Metabolism | E | 4 | 1 | 55 | 35787 | 8.44 |
| **7H** | SID807 | 0.010 | 0.878 | Guanine deaminase | gi|6753960 | Q9R111 | Gda | Nucleotide metabolism | E | 29 | 11 | 720 | 50981 | 5.36 |
| **7H** | SID788 | 0.013 | 0.859 | guanine deaminase | gi|6753960 | Q9R111 | Gda | Nucleotide metabolism | E | 29 | 11 | 720 | 50981 | 5.36 |
| **7H** | SID808 | 0.000 | 0.701 | guanine deaminase | gi|6753960 | Q9R111 | Gda | Nucleotide metabolism | E | 16 | 8 | 444 | 50981 | 5.36 |
| **7H** | BID1836 | 0.018 | 0.836 | glutamine synthetase | gi|31982332 | P15105 | Glul | AS Metabolism | E | 35 | 12 | 617 | 42092 | 6.64 |
| **7H** | BID1841 | 0.005 | 0.922 | glutamine synthetase | gi|31982332 | P15105 | Glul | AS Metabolism | E | 41 | 16 | 942 | 42092 | 6.64 |
| **7H** | SID1062 | 0.039 | 0.926 | guanine nucleotide-binding protein, beta-1 subunit | gi|6680045 | P62874 | Gnb1 | Signal transduction | E | 25 | 8 | 532 | 37353 | 5.6 |
| **7H** | SID1104 | 0.037 | 0.915 | Guanine nucleotide-binding protein G(I)/G(S)/G(T) subunit beta 2 | gi|984551 | P62880 | Gnb2 | Signal transduction | E | 36 | 9 | 597 | 37309 | 5.6 |
| **7H** | BID722 | 0.040 | 1.037 | mitochondrial aspartate aminotransferase | gi|2690302 | P05202 | Got2 | AS Metabolism | E | 33 | 12 | 769 | 47382 | 9.05 |
| **7H** | BID716 | 0.036 | 1.044 | mitochondrial aspartate aminotransferase | gi|192050 | P05202 | Got2 | AS Metabolism | E | 30 | 14 | 900 | 47723 | 9.05 |
| **7H** | BID723 | 0.041 | 1.059 | mitochondrial aspartate aminotransferase | gi|192050 | P05202 | Got2 | AS Metabolism | E | 28 | 12 | 732 | 47723 | 39211 |
| **7H** | BID819 | 0.044 | 0.827 | Glycerol-3-phosphate dehydrogenase [NAD+], cytoplasmic | gi|387177 | P13707 | Gpd1 | Lipid metabolism | E | 6 | 2 | 97 | 37560 | 6.75 |
| **7H** | BID1163 | 0.015 | 0.964 | glutathione S-transferase, mu type 3 | gi|13592152 | P19639 | Gstm3 | AS Metabolism | E | 9 | 2 | 119 | 25664 | 6.84 |
| **7H** | BID1675 | 0.002 | 0.814 | alpha-globin | gi|193761 | Q61649 | Hba-a1 |  | E | 25 | 1 | 50 | 6212 | 6.82 |
| **7H** | BID880 | 0.021 | 0.854 | heterogeneous nuclear ribonucleoprotein A0 isoform 2 | gi|82950644 | Q9CX86 | Hnrpa0 | Translation | E | 18 | 4 | 253 | 30512 | 9.35 |
| **7H** | BID900 | 0.021 | 1.107 | heterogeneous nuclear ribonucleoprotein A0 isoform 2 | gi|82950644 | Q9CX86 | Hnrpa0 | Translation | E | 31 | 5 | 373 | 30512 | 9.35 |
| **7H** | SID730 | 0.016 | 0.920 | heterogeneous nuclear ribonucleoprotein H2 | gi|9845253 | P70333 | Hnrph2 | Translation | E | 26 | 9 | 558 | 49248 | 5.89 |
| **7H** | SID5681 | 0.047 | 1.073 | heterogeneous nuclear ribonucleoprotein K | gi|13384620 | P61979 | Hnrpk | Transcription | E | 17 | 10 | 498 | 50944 | 5.39 |
| **7H** | SID806 | 0.036 | 0.460 | Homer protein homolog 1 (VASP/Ena-related gene up-regulated during seizure and LTP) (Vesl-1) | gi|38605093 | Q9Z2Y3 | Homer1 | Signal transduction | E | 26 | 8 | 538 | 41388 | 5.39 |
| **7H** | SID480 | 0.033 | 1.104 | 78 kDa glucose-regulated protein [Precursor], Bip | gi|2598562 | P20029 | Hspa5 | Folding | E | 30 | 16 | 1072 | 72378 | 5.07 |
| **7H** | SID680 | 0.036 | 1.079 | heat shock protein 65 | gi|51455 | P63038 | Hspd1 | Folding | E | 41 | 19 | 1434 | 60903 | 5.91 |
| **7H** | SID684 | 0.046 | 1.046 | heat shock protein 65 | gi|51455 | P63038 | Hspd1 | Folding | E | 42 | 21 | 1380 | 60903 | 5.91 |
| **7H** | SID693 | 0.002 | 1.103 | heat shock protein 65 | gi|51452 | P63038 | Hspd1 | Folding | E | 24 | 12 | 755 | 60903 | 5.91 |
| **7H** | BID561 | 0.027 | 1.060 | Isocitrate dehydrogenase [NADP] cytoplasmic (Cytosolic NADP-isocitrate dehydrogenase) (Oxalosuccina | gi|6647554 | O88844 | Idh1 | CH Metabolism | E | 37 | 15 | 899 | 46630 | 6.48 |
| **7H** | SID1022 | 0.033 | 1.071 | Isocitrate dehydrogenase [NAD] subunit alpha, mitochondrial [Precursor] | gi|18250284 | Q9D6R2 | Idh3a | CH Metabolism | E | 19 | 6 | 346 | 39613 | 6.27 |
| **7H** | SID1268 | 0.043 | 0.923 | Inositol monophosphatase | gi|3914098 | O55023 | Impa1 | Signal transduction | E | 20 | 5 | 332 | 30416 | 5.08 |
| **7H** | BID775 | 0.006 | 0.693 | Voltage-gated potassium channel subunit beta-2 | gi|975314 | P62482 | Kcnab2 |  | E | 3 | 1 | 78 | 41154 | 9.19 |
| **7H** | SID1096 | 0.022 | 0.876 | lactate dehydrogenase 2, B chain | gi|6678674 | P16125 | Ldhb | CH Metabolism | E | 23 | 7 | 446 | 36549 | 5.7 |
| **7H** | SID1107 | 0.041 | 0.932 | lactate dehydrogenase 2, B chain | gi|6678674 | P16125 | Ldhb | CH Metabolism | E | 18 | 5 | 277 | 36549 | 5.7 |
| **7H** | SID1186 | 0.049 | 0.962 | Malate dehydrogenase, cytoplasmic | gi|387129 | P14152 | Mdh1 | CH Metabolism | E | 15 | 5 | 279 | 36454 | 6.16 |
| **7H** | BID824 | 0.029 | 1.159 | malate dehydrogenase 2, NAD (mitochondrial) | gi|31982186 | Q8R1P0 | Mdh2 | CH Metabolism | E | 54 | 16 | 958 | 35589 | 8.93 |
| **7H** | SID15643 | 0.014 | 1.263 | 39S ribosomal protein L12, mitochondrial [Precursor] | gi|12837801 | Q9DB15 | Mrpl12 | Translation | E | 30 | 7 | 348 | 21695 | 9.34 |
| **7H** | SID15756 | 0.046 | 1.096 | Myosin light polypeptide 6 | gi|17986258 | Q60605 | Myl6 | Cytoskeleton | E | 31 | 5 | 310 | 16919 | 4.56 |
| **7H** | SID15758 | 0.028 | 0.869 | Myosin light polypeptide 6 | gi|17986258 | Q60605 | Myl6 | Cytoskeleton | E | 31 | 5 | 299 | 16919 | 4.56 |
| **7H** | SID15744 | 0.003 | 1.104 | Myosin light polypeptide 6 | gi|17986258 | Q60605 | Myl6 | Cytoskeleton | E | 31 | 4 | 277 | 16919 | 4.56 |
| **7H** | SID15252 | 0.032 | 0.905 | Nascent polypeptide-associated complex subunit alpha | gi|41350312 | Q60817 | Naca | Transcription | E | 12 | 2 | 163 | 23370 | 4.52 |
| **7H** | SID1122 | 0.021 | 0.967 | Beta-soluble NSF attachment protein | gi|29789104 | P28663 | Napb | Sorting | E | 40 | 10 | 678 | 33536 | 5.32 |
| **7H** | BID394 | 0.033 | 0.903 | Non-POU domain-containing octamer-binding protein | gi|12963531 | Q99K48 | Nono |  | E | 12 | 5 | 266 | 54564 | 8.86 |
| **7H** | BID1141 | 0.019 | 0.868 | Cleavage and polyadenylation specificity factor 5 | gi|13386106 | Q9CQF3 | Nudt21 | Translation | E | 29 | 7 | 374 | 26224 | 8.85 |
| **7H** | SID718 | 0.029 | 0.943 | protein kinase C and casein kinase substrate in neurons 1 | gi|6754974 | Q61644 | Pacsin1 | Cytoskeleton | E | 43 | 19 | 1136 | 50544 | 5.15 |
| **7H** | BID1180 | 0.038 | 0.883 | Protein-L-isoaspartate(D-aspartate) O-methyltransferase | gi|417489 | P23506 | Pcmt1 | Protein modification | E | 9 | 2 | 130 | 24619 | 7.1 |
| **7H** | SID1499 | 0.010 | 1.088 | pyruvate dehydrogenase (lipoamide) beta | gi|12805431 | Q99LW9 | Pdhb | CH Metabolism | E | 3 | 1 | 41 | 34814 | 5.63 |
| **7H** | BID1594 | 0.003 | 1.133 | profilin 1 | gi|6755040 | P62962 | Pfn1 | Cytoskeleton | E | 34 | 4 | 210 | 14948 | 8.46 |
| **7H** | BID604 | 0.027 | 0.904 | Phosphoglycerate kinase 1 | gi|129903 | P09411 | Pgk1 | CH Metabolism | E | 37 | 14 | 888 | 44508 | 7.53 |
| **7H** | SID1345 | 0.037 | 0.844 | 6-phosphogluconolactonase | gi|13384778 | Q9CQ60 | Pgls | CH Metabolism | E | 26 | 5 | 304 | 27237 | 5.55 |
| **7H** | BID171 | 0.049 | 0.822 | Phosphatase and actin regulator 1 | gi|26339202 | Q2M3X8 | Phactr1 | Cytoskeleton | E | 3 | 2 | 130 | 68184 | 6.69 |
| **7H** | SID696 | 0.035 | 0.873 | D-3-phosphoglycerate dehydrogenase | gi|52353955 | Q61753 | Phgdh | AS Metabolism | E | 15 | 6 | 427 | 56549 | 6.12 |
| **7H** | BID1453 | 0.023 | 1.103 | protein (peptidyl-prolyl cis/trans isomerase) NIMA-interacting 1 | gi|12963653 | Q9QUR7 | Pin1 | Cell growth and death | E | 21 | 2 | 165 | 18359 | 8.93 |
| **7H** | SID1050 | 0.006 | 0.782 | Serine/threonine-protein phosphatase PP1-alpha catalytic subunit | gi|13994195 | P62137 | Ppp1ca | Signal transduction | E | 7 | 2 | 106 | 37516 | 5.94 |
| **7H** | SID874 | 0.047 | 0.854 | Protein phosphatase 1 regulatory subunit 7 | gi|12963569 | Q3UM45 | Ppp1r7 | Signal transduction | E | 28 | 9 | 559 | 41266 | 4.85 |
| **7H** | BID1108 | 0.034 | 0.896 | proline synthetase co-transcribed isoform a | gi|16930823 | Q544R1 | Prosc |  | E | 33 | 8 | 474 | 30030 | 8.37 |
| **7H** | BID601 | 0.023 | 0.858 | guanine nucleotide exchange factor for ADP ribosylation factor 6 isoform 1 | gi|83921581 | Q8CEA6 | Psd3 | Signal transduction | E | 15 | 8 | 425 | 58430 | 8.92 |
| **7H** | SID1295 | 0.006 | 0.894 | Proteasome subunit alpha type 3 | gi|3914438 | O70435 | Psma3 | Degradation | E | 4 | 1 | 68 | 28387 | 5.29 |
| **7H** | SID1375 | 0.037 | 0.940 | proteasome (prosome, macropain) subunit, alpha type 5 | gi|7106387 | Q5E987 | Psma5 | Degradation | E | 17 | 3 | 216 | 26394 | 4.74 |
| **7H** | SID734 | 0.008 | 0.942 | 26S proteasome non-ATPase regulatory subunit 4 | gi|6679505 | O35226 | Psmd4 | Degradation | E | 23 | 7 | 455 | 40678 | 4.67 |
| **7H** | SID9163 | 0.022 | 0.801 | prostaglandin E synthase 3 (cytosolic) | gi|9790017 | Q9R0Q7 | Ptges3 | Lipid metabolism | E | 6 | 1 | 62 | 18709 | 4.36 |
| **7H** | SID1513 | 0.021 | 1.283 | prostaglandin E synthase 3 (cytosolic) | gi|26370131 | Q9CRH1 | Ptges3 | Lipid metabolism | E | 40 | 13 | 65 | 16561 | 4.81 |
| **7H** | SID9164 | 0.037 | 0.834 | prostaglandin E synthase 3 (cytosolic) | gi|26370131 | Q9CRH1 | Ptges3 | Lipid metabolism | M | 40 | 7 | 65 | 16561 | 4.81 |
| **7H** | SID964 | 0.007 | 1.084 | Transcriptional activator protein Pur-alpha | gi|34878862 | P42669 | Pura | Cell growth and death | E | 11 | 4 | 205 | 34862 | 6.07 |
| **7H** | SID15225 | 0.006 | 1.142 | Transcriptional activator protein Pur-beta | gi|6755252 | O35295 | Purb | Transcription | E | 12 | 3 | 164 | 33881 | 5.35 |
| **7H** | SID905 | 0.004 | 0.924 | protein 40kD | gi|226005 | P14206 | Rpsa | Translation | E | 20 | 5 | 298 | 32732 | 4.8 |
| **7H** | BID1053 | 0.002 | 0.847 | Ras suppressor protein 1 (Rsu-1) (RSP-1) | gi|548879 | Q01730 | Rsu1 | Signal transduction | E | 15 | 3 | 151 | 31531 | 8.86 |
| **7H** | SID15880 | 0.041 | 0.910 | Protein S100-A13 | gi|6677835 | P97352 | S100a13 | Signal transduction | E | 22 | 2 | 115 | 11151 | 5.89 |
| **7H** | SID15270 | 0.041 | 0.868 | Protein SEC13 homolog | gi|12805321 | Q9D1M0 | Sec13 |  | E | 3 | 1 | 62 | 35501 | 5.15 |
| **7H** | BID624 | 0.049 | 0.957 | Septin-5 | gi|3986409 | Q9Z2Q6 | Sept5 | Cytoskeleton | E | 10 | 3 | 154 | 40215 | 6.1 |
| **7H** | BID516 | 0.046 | 0.831 | Septin-7 | gi|28173550 | O55131 | Sept7 | Cytoskeleton | E | 25 | 11 | 639 | 50617 | 8.73 |
| **7H** | BID523 | 0.021 | 0.789 | Septin-7 | gi|28173550 | O55131 | Sept7 | Cytoskeleton | E | 36 | 14 | 882 | 50617 | 8.73 |
| **7H** | SID740 | 0.005 | 0.919 | Septin-8 | gi|29612695 | Q8CHH9 | Sept8 | Cytoskeleton | E | 11 | 5 | 308 | 52647 | 5.96 |
| **7H** | SID571 | 0.031 | 0.726 | Serine protease inhibitor A3K [Precursor] | gi|54173 | P07759 | Serpina3k |  | E | 3 | 1 | 76 | 46643 | 5.04 |
| **7H** | SID1380 | 0.045 | 0.893 | Synaptosomal-associated protein 25 | gi|6755588 | P60879 | Snap25 | Sorting | E | 50 | 9 | 539 | 23300 | 4.66 |
| **7H** | SID1052 | 0.038 | 0.844 | Serine racemase | gi|26389300 | Q9QZX7 | Srr | AS Metabolism | E | 19 | 4 | 242 | 36340 | 5.68 |
| **7H** | SID15699 | 0.012 | 0.752 | Stathmin | gi|9789995 | P54227 | Stmn1 | Signal transduction | E | 29 | 5 | 234 | 17264 | 5.76 |
| **7H** | SID15259 | 0.037 | 0.915 | Serine-threonine kinase receptor-associated protein | gi|6755682 | Q9Z1Z2 | Strap | Signal transduction | E | 43 | 11 | 707 | 38489 | 4.99 |
| **7H** | BID791 | 0.007 | 1.101 | Succinyl-CoA ligase [GDP-forming] subunit alpha, mitochondrial precursor | gi|9845299 | Q9WUM5 | Suclg1 | CH Metabolism | E | 8 | 2 | 109 | 34953 | 9.45 |
| **7H** | SID15732 | 0.048 | 0.722 | Small ubiquitin-related modifier 3 [Precursor] | gi|9910556 | Q9Z172 | Sumo3 | Degradation | E | 22 | 3 | 144 | 12422 | 5.67 |
| **7H** | SID15315 | 0.013 | 0.911 | Tropomyosin alpha-3 chain | gi|54912 | P21107 | Tpm3 | Cytoskeleton | E | 37 | 11 | 668 | 29003 | 4.75 |
| **7H** | SID516 | 0.033 | 0.877 | Ubiquilin-1 | gi|16307349 | Q8R317 | Ubqln1 | Degradation | E | 10 | 3 | 199 | 4.86 | 61937 |
| **7H** | SID15639 | 0.018 | 0.870 | ubiquitin carboxy-terminal hydrolase L1 | gi|6755929 | Q00981 | Uchl1 | Degradation | E | 17 | 5 | 245 | 24822 | 5.33 |
| **7H** | SID5639 | 0.019 | 1.089 | Transitional endoplasmic reticulum ATPase | gi|400712 | Q01853 | Vcp | Degradation | E | 19 | 12 | 721 | 89252 | 5.14 |
| **7H** | SID1409 | 0.037 | 1.047 | Voltage-dependent anion-selective channel protein 1 | gi|6755963 | Q60932 | Vdac1 | Signal transduction | E | 31 | 6 | 412 | 30737 | 8.62 |
| **15C** | SID15202 | 0.042 | 1.154 | apolipoprotein A-IV precursor - mouse | gi|91885 | Q01488 | Apoa4 | Lipid metabolism | E | 15 | 4 | 274 | 45455 | 5.77 |
| **15C** | SID2679 | 0.001 | 1.673 | Apolipoprotein E | gi|74178217 | P08226 | Apoe | Lipid metabolism | E | 26 | 9 | 515 | 35884 | 5.56 |
| **15C** | SID2064 | 0.047 | 0.957 | ATP synthase subunit beta, mitochondrial [Precursor] | gi|2623222 | P56480 | Atp5b | Energy Metabolism | E | 37 | 15 | 960 | 56632 | 5.24 |
| **15C** | SID775 | 0.025 | 0.941 | ATP synthase subunit beta, mitochondrial [Precursor] | gi|23272966 | P56480 | Atp5b | Energy Metabolism | E | 19 | 7 | 624 | 56632 | 5.24 |
| **15C** | SID792 | 0.021 | 0.900 | brain abundant, membrane attached signal protein 1 | gi|45598372 | Q91XV3 | Basp1 | Neuron | E | 51 | 7 | 494 | 22074 | 4.5 |
| **15C** | SID414 | 0.046 | 1.073 | Myc box-dependent-interacting protein 1 | gi|6753050 | O08539 | Bin1 | Cell growth and death | E | 10 | 4 | 233 | 64430 | 4.95 |
| **15C** | BID1383 | 0.018 | 0.906 | cofilin 1, non-muscle | gi|6680924 | P18760 | Cfl1 | Cytoskeleton | E | 33 | 4 | 270 | 18548 | 8.22 |
| **15C** | SID15778 | 0.002 | 1.052 | coactosin-like 1 | gi|19482160 | Q9CQI6 | Cotl1 | Cytoskeleton | E | 37 | 10 | 99 | 15934 | 5.28 |
| **15C** | BID1138 | 0.018 | 0.898 | es1 protein | gi|20070420 | Q9D172 | D10Jhu81e |  | E | 13 | 4 | 178 | 28073 | 9 |
| **15C** | BID1723 | 0.010 | 1.080 | diazepam binding inhibitor isoform 2 | gi|6681137 | P31786 | Dbi | Lipid metabolism | E | 32 | 2 | 77 | 9994 | 8.78 |
| **15C** | SID522 | 0.027 | 0.934 | Dihydropyrimidinase-related protein 2 | gi|1915913 | O08553 | Dpysl2 | Neuron | E | 57 | 39 | 228 | 62132 | 5.95 |
| **15C** | BID298 | 0.008 | 0.907 | dihydropyrimidinase-related protein 4 | gi|34328211 | O35098 | Dpysl4 |  | E | 13 | 6 | 371 | 61922 | 6.51 |
| **15C** | BID396 | 0.006 | 1.030 | dihydropyrimidinase-related protein 4 | gi|34328211 | O35098 | Dpysl4 |  | E | 13 | 6 | 371 | 61922 | 6.51 |
| **15C** | BID1740 | 0.050 | 0.967 | dynein light chain 2 | gi|18087731 | Q9D0M5 | Dynll2 | Cytoskeleton | E | 24 | 1 | 60 | 10343 | 6.81 |
| **15C** | BID495 | 0.035 | 1.049 | elongation factor Tu | gi|556301 | P10126 | Eef1a1 | Translation | E | 8 | 4 | 207 | 50132 | 9.1 |
| **15C** | SID832 | 0.003 | 1.122 | enolase 2, gamma neuronal | gi|7305027 | P17183 | Eno2 | CH Metabolism | E | 20 | 8 | 439 | 47267 | 4.99 |
| **15C** | SID836 | 0.020 | 1.209 | enolase 2, gamma neuronal | gi|7305027 | P17183 | Eno2 | CH Metabolism | E | 20 | 8 | 439 | 47267 | 4.99 |
| **15C** | SID1324 | 0.030 | 1.069 | Endoplasmic reticulum protein ERp29 | gi|19526463 | P57759 | Erp29 | Sorting | E | 25 | 6 | 357 | 28805 | 5.9 |
| **15C** | SID15849 | 0.034 | 1.554 | fatty acid binding protein 3, muscle and heart | gi|6753810 | P11404 | Fabp3 | Lipid metabolism | E | 45 | 8 | 65 | 14810 | 6.11 |
| **15C** | SID788 | 0.014 | 0.884 | guanine deaminase | gi|6753960 | Q9R111 | Gda | Nucleotide metabolism | E | 29 | 11 | 720 | 50981 | 5.36 |
| **15C** | SID838 | 0.001 | 2.362 | glial fibrillary acidic protein | gi|84000448 | P03995 | Gfap | Cytoskeleton | E | 49 | 25 | 1431 | 49870 | 5.27 |
| **15C** | SID848 | 0.005 | 1.289 | glial fibrillary acidic protein | gi|319914 | P03995 | Gfap | Cytoskeleton | E | 49 | 25 | 1431 | 49870 | 5.27 |
| **15C** | SID1255 | 0.045 | 0.780 | Glod4 protein | gi|12840311 | Q9CPV4 | Glod4 | Metabolism | E | 7 | 2 | 105 | 30765 | 4.8 |
| **15C** | BID715 | 0.042 | 1.044 | mitochondrial aspartate aminotransferase | gi|192050 | P05202 | Got2 | AS Metabolism | E | 19 | 8 | 520 | 47723 | 9.05 |
| **15C** | BID716 | 0.012 | 1.032 | mitochondrial aspartate aminotransferase | gi|192050 | P05202 | Got2 | AS Metabolism | E | 30 | 14 | 900 | 47723 | 9.05 |
| **15C** | BID922 | 0.004 | 0.675 | 3-hydroxyacyl CoA dehydrogenase | gi|1125026 | Q61425 | Hadh | Lipid metabolism | E | 7 | 2 | 103 | 33067 | 7.74 |
| **15C** | BID234 | 0.015 | 0.918 | Hydroxyacyl-Coenzyme A dehydrogenase/3-ketoacyl-Coenzyme A thiolase/enoyl-Coenzyme A hydratase (Trifunctional protein), alpha subunit | gi|33859811 | Q5U5Y5 | Hadha | Lipid metabolism | E | 19 | 12 | 770 | 82618 | 9.24 |
| **15C** | SID380 | 0.025 | 1.034 | heat shock protein 1, beta | gi|40556608 | P11499 | Hsp90ab1 | Folding | E | 44 | 62 | 279 | 83229 | 4.97 |
| **15C** | SID410 | 0.010 | 1.087 | heat shock protein 1, beta | gi|40556608 | P11499 | Hsp90ab1 | Folding | E | 44 | 62 | 279 | 83229 | 4.97 |
| **15C** | SID504 | 0.050 | 1.118 | Heat shock-related 70 kDa protein 2 | gi|109946 | P17156 | Hspa2 |  | E | 44 | 19 | 1134 | 60903 | 5.91 |
| **15C** | SID527 | 0.045 | 1.026 | Heat shock protein 8 | gi|42542422 | Q6NZD0 | Hspa8 | Folding | E | 40 | 20 | 1361 | 70648 | 5.22 |
| **15C** | SID1063 | 0.035 | 0.917 | isocitrate dehydrogenase 3 (NAD+) alpha | gi|18250284 | Q9D6R2 | Idh3a | CH Metabolism | E | 7 | 3 | 177 | 39613 | 6.27 |
| **15C** | SID1088 | 0.013 | 0.920 | lactate dehydrogenase 2, B chain | gi|6678674 | P16125 | Ldhb | CH Metabolism | E | 28 | 11 | 697 | 36549 | 5.7 |
| **15C** | SID15758 | 0.044 | 0.777 | Myosin light polypeptide 6 | gi|17986258 | Q60605 | Myl6 | Cytoskeleton | E | 31 | 5 | 299 | 16919 | 4.56 |
| **15C** | SID15744 | 0.007 | 1.208 | Myosin light polypeptide 6 | gi|17986258 | Q60605 | Myl6 | Cytoskeleton | E | 31 | 4 | 277 | 16919 | 4.56 |
| **15C** | SID482 | 0.048 | 1.092 | NADH dehydrogenase (ubiquinone) Fe-S protein 1 | gi|21704020 | Q91VD9 | Ndufs1 | Energy Metabolism | E | 5 | 3 | 184 | 79698 | 5.51 |
| **15C** | SID1316 | 0.031 | 0.944 | Platelet-activating factor acetylhydrolase IB beta subunit (PAF acetylhydrolase 30 kDa subunit) (PA | gi|3024348 | Q61206 | Pafah1b2 | Lipid metabolism | E | 5 | 2 | 124 | 25476 | 5.78 |
| **15C** | SID1118 | 0.035 | 0.827 | pyruvate dehydrogenase (lipoamide) beta | gi|18152793 | Q9D051 | Pdhb | CH Metabolism | E | 6 | 2 | 124 | 38912 | 6.41 |
| **15C** | SID15624 | 0.027 | 0.768 | Phosphatidylethanolamine-binding protein 1 | gi|1517864 | P70296 | Pebp1 | Lipid metabolism | E | 6 | 1 | 75 | 20847 | 5.19 |
| **15C** | BID197 | 0.037 | 0.945 | 6-phosphofructokinase, muscle type (Phosphofructokinase 1) (Phosphohexokinase) (Phosphofructo-1-kin | gi|13638207 | P47857 | Pfkm | CH Metabolism | E | 18 | 13 | 735 | 85215 | 8.24 |
| **15C** | BID1594 | 0.018 | 1.050 | profilin 1 | gi|6755040 | P62962 | Pfn1 | Cytoskeleton | E | 34 | 4 | 210 | 14948 | 8.46 |
| **15C** | BID1444 | 0.044 | 1.063 | protein (peptidyl-prolyl cis/trans isomerase) NIMA-interacting 1 | gi|12963653 | Q9QUR7 | Pin1 | Cell growth and death | E | 36 | 4 | 273 | 18359 | 8.93 |
| **15C** | SID893 | 0.043 | 0.865 | Protein phosphatase methylesterase 1 | gi|20809834 | Q8BVQ5 | Ppme1 | SecMetabolites | E | 23 | 8 | 465 | 42287 | 5.58 |
| **15C** | SID15634 | 0.025 | 1.044 | Peroxiredoxin-2 | gi|3603241 | Q61171 | Prdx2 | Degradation | M | 37 | 7 | 115 | 21778 | 5.2 |
| **15C** | BID1507 | 0.036 | 1.098 | peroxiredoxin 5 | gi|10129957 | P99029 | Prdx5 | Degradation | E | 48 | 8 | 494 | 17004 | 7.71 |
| **15C** | SID964 | 0.037 | 1.123 | Transcriptional activator protein Pur-alpha | gi|34878862 | P42669 | Pura | Cell growth and death | E | 11 | 4 | 205 | 34862 | 6.07 |
| **15C** | SID791 | 0.011 | 0.873 | Histone-lysine N-methyltransferase, H3 lysine-4 specific SET7 (Histone H3-K4 methyltransferase) (H3 | gi|25091213 | Q8VHL1 | Setd7 | AS Metabolism | M | 34 | 8 | 85 | 40481 | 4.53 |
| **15C** | BID1271 | 0.023 | 0.760 | manganese superoxide dismutase | gi|53450 | P09671 | Sod2 | Degradation | E | 16 | 3 | 202 | 24662 | 8.8 |
| **15C** | SID15266 | 0.007 | 1.117 | Serine-threonine kinase receptor-associated protein | gi|26344646 | Q8C6F6 | Strap | Signal transduction | E | 45 | 36 | 121 | 38418 | 4.99 |
| **15C** | BID278 | 0.012 | 0.905 | Syntaxin-binding protein 1 | gi|6981602 | O08599 | Stxbp1 | Sorting | E | 13 | 8 | 453 | 67526 | 6.49 |
| **15C** | BID285 | 0.029 | 0.920 | Syntaxin-binding protein 1 | gi|6981602 | O08599 | Stxbp1 | Sorting | E | 13 | 8 | 453 | 67526 | 6.49 |
| **15C** | BID292 | 0.021 | 0.905 | Syntaxin-binding protein 1 | gi|6981602 | O08599 | Stxbp1 | Sorting | E | 13 | 8 | 453 | 67526 | 6.49 |
| **15C** | BID201 | 0.019 | 0.871 | synapsin Ib | gi|3599473 | O88935 | SynI | Neuron | E | 9 | 4 | 273 | 69992 | 9.84 |
| **15C** | BID1168 | 0.035 | 0.959 | triosephosphate isomerase | gi|1864018 | P17751 | Tpi1 | CH Metabolism | E | 40 | 7 | 394 | 22492 | 5.62 |
| **15C** | SID15276 | 0.015 | 1.292 | ubiquitin-conjugating enzyme E2R 2 | gi|13385778 | Q6ZWZ2 | Ube2r2 | Degradation | E | 22 | 12 | 56 | 27149 | 4.26 |
| **15C** | BID1889 | 0.015 | 0.950 | UBX domain-containing protein 1 | gi|13277354 | Q99PL6 | Ubxd1 |  | E | 13 | 4 | 258 | 49765 | 8.7 |
| **15C** | SID856 | 0.020 | 1.045 | Ubiquinol-cytochrome-c reductase complex core protein 1, mitochondrial [Precursor] | gi|14548301 | Q9CZ13 | Uqcrc1 | Energy Metabolism | M | 53 | 33 | 183 | 48000 | 9.26 |
| **15C** | SID5639 | 0.006 | 1.068 | Transitional endoplasmic reticulum ATPase | gi|400712 | Q01853 | Vcp | Degradation | E | 19 | 12 | 721 | 89252 | 5.14 |
| **15C** | SID843 | 0.002 | 1.674 | Transitional endoplasmic reticulum ATPase | gi|400712 | Q01853 | Vcp | Degradation | E | 29 | 82 | 104 | 89252 | 5.14 |
| **15C** | SID844 | 0.005 | 1.430 | Transitional endoplasmic reticulum ATPase | gi|400712 | Q01853 | Vcp | Degradation | E | 29 | 82 | 104 | 89252 | 5.14 |
| **15H** | BID1875 | 0.027 | 0.761 | 4-aminobutyrate aminotransferase | gi|37202121 | P61922 | Abat | AS Metabolism | E | 22 | 9 | 586 | 50205 | 8.79 |
| **15H** | SID927 | 0.034 | 0.897 | Actin, cytoplasmic 1 | gi|49868 | P60710 | Actb | Cytoskeleton | E | 13 | 4 | 232 | 39161 | 5.78 |
| **15H** | SID920 | 0.006 | 0.923 | Actin, cytoplasmic 2 | gi|809561 | P63260 | Actg1 | Cytoskeleton | E | 32 | 10 | 522 | 40992 | 5.56 |
| **15H** | BID693 | 0.030 | 1.136 | aldolase 1, A isoform | gi|6671539 | Q5FWB7 | Aldoa | CH Metabolism | E | 49 | 15 | 1021 | 39331 | 8.31 |
| **15H** | SID2679 | 0.003 | 1.225 | Apolipoprotein E | gi|74178217 | P08226 | Apoe | Lipid metabolism | E | 26 | 9 | 515 | 35884 | 5.56 |
| **15H** | SID2062 | 0.010 | 0.859 | ATP synthase subunit beta, mitochondrial [Precursor] | gi|23272966 | P56480 | Atp5b | Energy Metabolism | E | 17 | 6 | 337 | 56632 | 5.24 |
| **15H** | SID4795 | 0.006 | 1.186 | calbindin 2 | gi|34098931 | Q8CCS7 | Calb2 | Signal transduction | E | 14 | 3 | 155 | 28105 | 4.92 |
| **15H** | BID400 | 0.040 | 0.956 | T-complex protein 1 subunit eta | gi|549060 | P80313 | Cct7 | Folding | E | 49 | 21 | 1350 | 59614 | 7.95 |
| **15H** | SID15778 | 0.015 | 0.944 | coactosin-like 1 | gi|19482160 | Q9CQI6 | Cotl1 | Cytoskeleton | E | 37 | 10 | 99 | 15934 | 5.28 |
| **15H** | BID1138 | 0.025 | 0.726 | es1 protein | gi|20070420 | Q9D172 | D10Jhu81e |  | E | 13 | 4 | 178 | 28073 | 9 |
| **15H** | BID1723 | 0.028 | 1.130 | diazepam binding inhibitor isoform 2 | gi|6681137 | P31786 | Dbi | Lipid metabolism | E | 32 | 2 | 77 | 9994 | 8.78 |
| **15H** | SID1027 | 0.005 | 0.852 | NG,NG-dimethylarginine dimethylaminohydrolase 1 | gi|38371755 | Q9CWS0 | Ddah1 | Signal transduction | E | 41 | 12 | 602 | 31361 | 5.64 |
| **15H** | SID5680 | 0.016 | 1.050 | Dihydrolipoamide S-acetyltransferase | gi|20071885 | Q8R339 | Dlat | CH Metabolism | E | 17 | 7 | 476 | 59047 | 5.71 |
| **15H** | BID298 | 0.035 | 0.886 | dihydropyrimidinase-related protein 4 | gi|34328211 | O35098 | Dpysl4 |  | E | 13 | 6 | 371 | 61922 | 6.51 |
| **15H** | BID496 | 0.039 | 1.143 | enolase 1, alpha non-neuron | gi|53734652 | Q5FW97 | Eno1 | CH Metabolism | E | 19 | 7 | 421 | 50209 | 8.01 |
| **15H** | SID15269 | 0.027 | 1.575 | enolase 2, gamma neuronal | gi|7305027 | P17183 | Eno2 | CH Metabolism | E | 17 | 4 | 301 | 47267 | 4.99 |
| **15H** | SID15823 | 0.015 | 0.923 | Fatty acid-binding protein, heart | gi|387397 | Q91W23 | Fabp3 | Lipid metabolism | E | 35 | 4 | 205 | 14810 | 6.11 |
| **15H** | SID838 | 0.004 | 1.517 | glial fibrillary acidic protein | gi|14193690 | P03995 | Gfap | Cytoskeleton | E | 49 | 25 | 1431 | 49870 | 5.27 |
| **15H** | SID15240 | 0.019 | 0.795 | guanine nucleotide-binding protein, beta-1 subunit | gi|6680045 | P62874 | Gnb1 | Signal transduction | E | 9 | 3 | 175 | 37353 | 5.6 |
| **15H** | SID1104 | 0.038 | 0.958 | Guanine nucleotide-binding protein G(I)/G(S)/G(T) subunit beta 2 | gi|984551 | P62880 | Gnb2 | Signal transduction | E | 36 | 9 | 597 | 37309 | 5.60 |
| **15H** | BID1132 | 0.026 | 0.855 | glutathione S-transferase, mu 1 | gi|6754084 | Q58ET5 | Gstm1 | AS Metabolism | E | 55 | 13 | 724 | 25953 | 7.71 |
| **15H** | BID922 | 0.029 | 0.453 | 3-hydroxyacyl CoA dehydrogenase | gi|1125026 | Q61425 | Hadh | Lipid metabolism | E | 7 | 2 | 103 | 33067 | 7.74 |
| **15H** | SID380 | 0.042 | 1.101 | heat shock protein 1, beta | gi|40556608 | P11499 | Hsp90ab1 | Folding | E | 30 | 16 | 1072 | 72378 | 5.07 |
| **15H** | BID561 | 0.049 | 1.036 | Isocitrate dehydrogenase [NADP] cytoplasmic (Cytosolic NADP-isocitrate dehydrogenase) (Oxalosuccina | gi|6647554 | O88844 | Idh1 | CH Metabolism | E | 37 | 15 | 899 | 46630 | 6.48 |
| **15H** | SID1268 | 0.032 | 0.921 | Inositol monophosphatase | gi|3914098 | O55023 | Impa1 | Signal transduction | E | 20 | 5 | 332 | 30416 | 5.08 |
| **15H** | SID1146 | 0.011 | 0.753 | lactate dehydrogenase 2, B chain | gi|6678674 | P16125 | Ldhb | CH Metabolism | E | 28 | 11 | 697 | 36549 | 5.7 |
| **15H** | SID15218 | 0.006 | 1.043 | nascent polypeptide-associated complex alpha polypeptide [Mus musculus] | gi|41350312 | Q60817 | Naca | Transcription | E | 12 | 2 | 163 | 23370 | 4.52 |
| **15H** | SID15243 | 0.019 | 0.816 | Gamma-soluble NSF attachment protein | gi|26355647 | Q9CWZ7 | Napg | Sorting | E | 34 | 11 | 667 | 34710 | 5.31 |
| **15H** | BID171 | 0.048 | 0.581 | Phosphatase and actin regulator 1 | gi|26339202 | Q2M3X8 | Phactr1 | Cytoskeleton | E | 3 | 2 | 130 | 68184 | 6.69 |
| **15H** | BID1444 | 0.023 | 1.138 | protein (peptidyl-prolyl cis/trans isomerase) NIMA-interacting 1 | gi|12963653 | Q9QUR7 | Pin1 | Cell growth and death | E | 36 | 4 | 273 | 18359 | 8.93 |
| **15H** | BID350 | 0.041 | 0.929 | pyruvate kinase M | gi|31981562 | P52480 | Pkm2 | CH Metabolism | E | 44 | 19 | 1215 | 57808 | 7.18 |
| **15H** | BID644 | 0.025 | 0.873 | Transcriptional activator protein Pur-alpha | gi|6679573 | P42669 | Pura | Cell growth and death | E | 11 | 4 | 205 | 34862 | 6.07 |
| **15H** | SID15796 | 0.004 | 0.813 | Parvalbumin alpha | gi|53819 | P32848 | Pvalb | Signal transduction | E | 52 | 5 | 279 | 11937 | 5.02 |
| **15H** | SID763 | 0.034 | 0.936 | reticulocalbin 2 | gi|74146742 | Q3TE95 | Rcn2 |  | E | 36 | 9 | 639 | 41014 | 4.46 |
| **15H** | BID674 | 0.045 | 0.915 | Neuronal-specific septin-3 | GI:13124538 | Q9Z1S5 | Sept3 | Cytoskeleton | E | 8 | 4 | 197 | 52756 | 7.45 |
| **15H** | BID678 | 0.028 | 0.919 | Neuronal-specific septin-3 | gi|13124538 | Q9Z1S5 | Sept3 | Cytoskeleton | E | 4 | 2 | 99 | 52756 | 7.45 |
| **15H** | BID631 | 0.022 | 0.917 | Septin-5 | gi|83305642 | Q9Z2Q6 | Sept5 | Cytoskeleton | E | 26 | 9 | 474 | 42721 | 6.21 |
| **15H** | BID540 | 0.025 | 0.876 | Septin-7 | gi|9789726 | O55131 | Sept7 | Cytoskeleton | E | 10 | 4 | 251 | 50518 | 8.73 |
| **15H** | BID516 | 0.011 | 0.829 | Septin-7 | gi|28173550 | O55131 | Sept7 | Cytoskeleton | E | 25 | 11 | 639 | 50617 | 8.73 |
| **15H** | BID523 | 0.007 | 0.833 | Septin-7 | gi|28173550 | O55131 | Sept7 | Cytoskeleton | E | 36 | 14 | 882 | 50617 | 8.73 |
| **15H** | SID791 | 0.006 | 0.868 | Histone-lysine N-methyltransferase, H3 lysine-4 specific SET7 (Histone H3-K4 methyltransferase) (H3 | gi|25091213 | Q8VHL1 | Setd7 | AS Metabolism | M | 34 | 8 | 85 | 40481 | 4.53 |
| **15H** | BID278 | 0.047 | 0.911 | Syntaxin-binding protein 1 | gi|6981602 | O08599 | Stxbp1 | Sorting | E | 13 | 8 | 453 | 67526 | 6.49 |
| **15H** | SID939 | 0.024 | 1.116 | Succinyl-CoA ligase [GDP-forming] beta-chain, mitochondrial precursor (Succinyl-CoA synthetase, bet | gi|85681897 | Q9Z2I8 | Suclg2 | CH Metabolism | E | 36 | 14 | 129 | 46811 | 6.58 |
| **15H** | BID1186 | 0.013 | 0.904 | triosephosphate isomerase | gi|1864018 | Q64513 | Tpi1 | CH Metabolism | E | 40 | 7 | 419 | 5.62 |  |
| **15H** | BID1185 | 0.042 | 0.921 | triosephosphate isomerase | gi|1864018 | Q64513 | Tpi1 | CH Metabolism | E | 26 | 4 | 271 | 22492 | 5.62 |
| **15H** | SID15315 | 0.007 | 0.919 | Tropomyosin alpha-3 chain | gi|54912 | P21107 | Tpm3 | Cytoskeleton | E | 37 | 11 | 668 | 29003 | 4.75 |
| **15H** | SID15279 | 0.023 | 0.821 | ubiquitin-conjugating enzyme E2R 2 | gi|13385778 | Q6ZWZ2 | Ube2r2 | Degradation | E | 22 | 12 | 56 | 27149 | 4.26 |
| **15H** | SID515 | 0.050 | 1.062 | Ubiquilin-1 | gi|16307349 | Q8R317 | Ubqln1 | Degradation | E | 10 | 3 | 199 | 4.86 | 61937 |
| **15H** | SID843 | 0.006 | 1.277 | Transitional endoplasmic reticulum ATPase | gi|400712 | Q01853 | Vcp | Degradation | E | 29 | 82 | 104 | 89252 | 5.14 |
| **15H** | SID844 | 0.010 | 1.244 | Transitional endoplasmic reticulum ATPase | gi|400712 | Q01853 | Vcp | Degradation | E | 29 | 82 | 104 | 89252 | 5.14 |
| **15H** | BID1001 | 0.004 | 1.137 | Voltage-dependent anion channel 3 | gi|59807690 | Q5EBQ0 | Vdac3 | Signal transduction | E | 31 | 7 | 472 | 30864 | 8.96 |
|  |  |  |  |  |  |  |  |  |  |  |  |  |  |  |
| a) E: LC-MS/MS; M: MALDI-TOF-MS. | | | |  |  |  |  |  |  |  |  |  |  |  |
| b) Identification was repeated when only 1 peptide was obtained. | | | | |  |  |  |  |  |  |  |  |  |  |
